# Floristic and structural assessment of Australian rangeland vegetation with standardized plot-based surveys

**DOI:** 10.1371/journal.pone.0202073

**Published:** 2018-09-07

**Authors:** Zdravko Baruch, Stefan Caddy-Retalic, Greg R. Guerin, Ben Sparrow, Emrys Leitch, Andrew Tokmakoff, Andrew J. Lowe

**Affiliations:** 1 Terrestrial Ecosystem Research Network, University of Adelaide Node, North Terrace, Adelaide, South Australia, Australia; 2 School of Biological Sciences, University of Adelaide, North Terrace, Adelaide, South Australia, Australia; 3 School of Life and Environmental Sciences, University of Sydney, Sydney, New South Wales, Australia; College of Agricultural Sciences, UNITED STATES

## Abstract

We describe and correlate environmental, floristic and structural vegetation traits of a large portion of Australian rangelands. We analysed 351 one hectare vegetation plots surveyed by Australia’s Terrestrial Ecosystem Research Network (TERN) using the AusPlots Rangelands standardized method. The AusPlots Rangelands method involves surveying 1010 one meter-spaced point-intercepts (IPs) per plot. At each IP, species were scored, categorised by growth-form, converted to percentage cover as the input for the plot _x_ species matrix. Vegetation structure is depicted by growth-form configuration and relative importance. The floristic and structural distance matrices were correlated with the Mantel test. Canonical correspondence analysis (CCA) related floristic composition to environmental variables sourced from WorldClim, the Atlas of Living Australia and TERN’s Soil and Landscape Grid. Differences between clusters were tested with ANOVA while principal component analysis (PCA) ordered the plots within the environmental space. Our plot _x_ species matrix required segmentation due to sparsity and high β-diversity. Based on the ordination of plots latitude within environmental space, the matrix was segmented into three “superclusters”: the winter rain and temperate Mediterranean, the monsoonal rain savannas and the arid deserts. Further classification, with the UPGMA linkage method, generated two, four and five clusters, respectively. All groupings are described by species richness, diversity indices and growth form conformation. Several floristic disjunctions were apparent and their possible causes are discussed. For all superclusters, the correspondence between the floristic and the structural or growth form matrices was statistically significant. CCA ordination clearly demarcated all groupings. Aridity, rainfall, temperature, seasonality, soil nitrogen and pH are significant correlates to the ordination of superclusters and clusters. At present, our results are influenced by incomplete sampling. As more sites are surveyed, this pioneer analysis will be updated and refined providing tools for the effective management of Australian rangelands.

## Introduction

**Fig 1:**
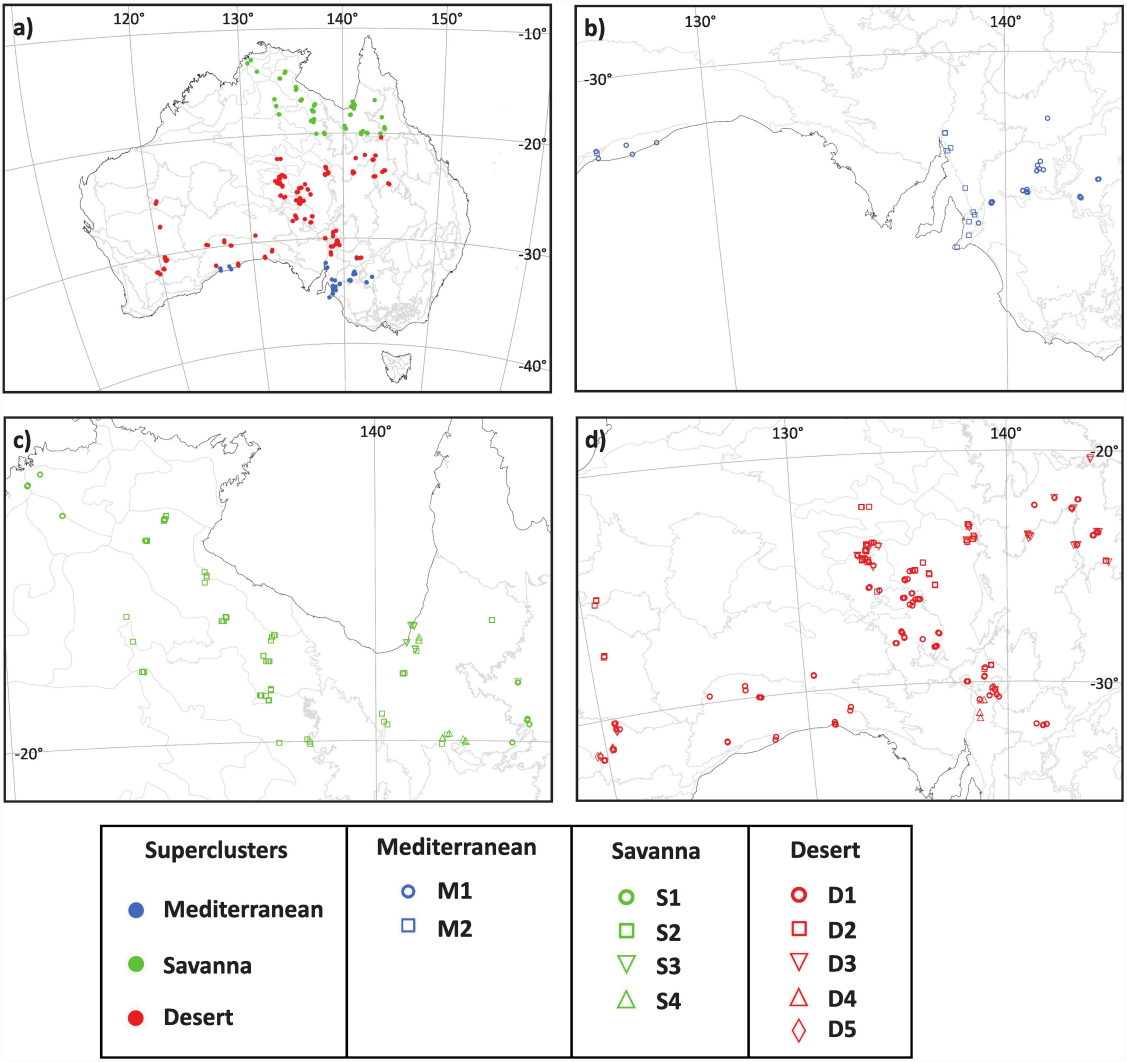
Geographical location of survey plots. (a) All sampled AusPlots grouped into superclusters. (b) Plots from clusters M1- M2 within the Mediterranean supercluster. (c) Plots from clusters S1–S4 within the savanna supercluster. (d) Plots from clusters D1- D5 within the desert supercluster. Letterings in the map refer to approximate position of places cited in the text. Flinders Lofty Block (FLB); Gibson Desert (GB); Great Australian Bight (GAB); Great Victoria Desert (GVD); Gulf of Carpentaria (GC); Longreach (L); Mitchell Grass Downs (MGD); North Eastern South Australia (NESA). Figure is for reference only as it not possible to discriminate plots due to the small scale of the maps. All *AusPlots* are fully represented in S1 Table.

In Australia, rangelands are defined as land supporting low-intensity, extensive or nomadic livestock grazing. Rangelands extend over 81% of the continent, embracing the northern monsoonal savannas and the southern temperate lands that bound the central deserts [1, 2] (Fig 1a). Rangelands are home to a large and threatened part of Australian biodiversity including endemic species, refugia and hotspots [1, 3–6] and a substantial pool of stored carbon [1, 7]. They also contribute significantly to Australia’s economy through pastoralism, mining and tourism [1] and are home to many Aboriginal communities.

Australian rangelands encompass diverse geological and old substrates, with mostly infertile soils that receive highly variable rainfall influenced by El Niño/La Niña cycles and by inter-decadal Pacific oscillations [8–11]. This combination of abiotic traits, modulated by fire and human intervention, shapes the vastly heterogeneous rangeland landscapes and vegetation types, which are challenging to describe and comprehend. Consequently, no integrated, floristic and structural quantitative treatment of vegetation has been published for the arid, Australian rangelands [2, 12, 13]. This shortcoming has been attributed to biophysical heterogeneity, geographical vastness, distance from population centres, the division of the region into several administrative entities and financial constraints [8, 10]. At a regional scale there are studies exploring floristic-environmental relationships [2, 13–20]. However, these scattered reports are difficult to compare directly due to results from diverse sampling methods and numerical analyses [2, 13].

The Terrestrial Ecosystem Research Network (TERN), overcomes the incompatibility of previous vegetation data sets by devising and implementing a standardized and nationally accepted vegetation survey and data processing method [21, 22]. As a result, for a large section of the rangelands, we are now able to describe and quantify environmental, floristic and structural diversity. Although there are smaller monitoring plot networks distributed across macro-ecological gradients in Australia [23], none has the scale in neither area nor biophysical contrasts as those covered by the AusPlots network. Worldwide, it is comparable to trans-continental endeavours such as the Kalahari Transect, the African Savannas on the Long Term Program, the US National Ecosystem Assessment and the North East China Transect [24–27].

This study provides the first integrated floristic and structural description of rangeland vegetation using data collected using the AusPlots Rangelands methodology. Also, we propose an exploratory vegetation classification scheme based on quantitative data and objective multivariate analysis complemented with descriptions of environmental, floristic and structural diversity. We also correlate the floristic composition and cover of surveyed plots to their growth-form importance and to significant environmental variables to understand the abiotic context for variation in rangeland vegetation. Where possible, the ecological implications of our results are compared to previous regional studies but we do not seek to replicate nor repeat the broad-scale descriptions of Australian rangeland vegetation well covered elsewhere [28–30].

Our results provide partial answers to several of the big ecological questions, related to the trends in vegetation structure and biodiversity under future climatic changes that inhibit effective environmental management in Australia [31]. TERN is continuing to expand the AusPlots network through new surveys and data processing activities. Therefore, analyses and outcomes with substantially enlarged data sets will be updated and refined periodically.

## Methods

### Data collection

This study includes data from one hectare rangeland plots surveyed between 2011 and 2014, with a few plots bordering the Mediterranean zone in the south but excluding the cool temperate highlands of Victoria and New South Wales (Fig 1a; S1 Table). The sampling design and survey method employed across the plot network are exhaustively treated in the AusPlots Rangelands Survey Protocol Manual [22] as well as the method justification [32], data systems [21] and preliminary analysis [4]. In brief, site selection was supported by ecological (e.g. stratification across representative bioregions and sampling ‘best on offer’ habitats), administrative, and logistic considerations (e.g. feasibility of access). At each site, a plot with a 100 x 100 m grid was established enclosing 5 N-S and 5 E-W 100 m long transects, providing a total of 1010 one meter-spaced intercept points (IPs). For each IP, the species present was scored, identified and categorized by growth-form. Herbarium vouchers were taken for each species and sent to major herbaria for formal identification [22, 32]. All sites were sampled under permits issued by State and Territory authorities and with individual permission from private landholders as follows: NSW- Office of Environment and Heritage and NSW—National Parks and Wildlife Service (Western LLS, Murray LLS, Riverina LLS); NT- Parks and Wildlife Commission Northern Territory (multiple locations); QLD—Department of Environment and Heritage Protection (multiple locations); SA—Department of Environment, Water and Natural Resources (whole State except Wilderness Protection Areas); VIC—Department of Environment and Primary Industries (Murray-Sunset NP, Alpine NP); WA—Department of Parks and Wildlife Biodiversity, Conservation and Attractions (whole state).

### The floristic and structural datasets

Species IPs per plot, converted to cover percentage (Cover Percentage = [IP/1010]*100), were entered into the plot _x_ species matrix. Obvious outlier plots, whose floristic distance (see below) was more than two standard deviations from the mean [33], were deleted. The resulting data set comprised 351 plots (S1 Table and S1, S2 and S3 Files). The coefficient of variation of row (species cover in plots) totals was 64%, which is moderate and does not require further transformation [33]. Vegetation structure is established on species growth-form described by the Australian Soil and Land Survey Field Handbook [34] (S2 Table). For each cluster (arising from our classification scheme, see below), the importance of individual growth-forms was obtained by adding the relative number of species and the relative number of IPs (max. importance = 200). The importance of each growth-form generated structural profiles (or spectra) for description and comparison between clusters. Also, for each cluster and for relevant growth-forms, the most important species were established by adding their relative IPs and their relative frequency (number of presences) in plots. Again, the maximum species importance value is 200. Photographs of the representative vegetated landscapes are shown for visual aid (S4 File).

#### Clustering and ordination

For floristically based analyses, we selected the Sørensen ecological distance (mathematically equivalent to the Bray-Curtis coefficient) [33] as the most appropriate for our vegetation matrix [33, 35]. An initial assessment revealed that our large dataset required to be segmented before subsequent clustering with the group average (UPGMA) linkage method. The relevance of the initial segmentation and the resulting clusters was appraised by their relative heterogeneity evaluated with the Whittaker’s β-diversity index (total group species richness / mean plot species richness [36]. One-way ANOVAs were employed to test differences between clusters of several compositional indexes such as their β-diversity, species richness, the Shannon diversity index (H’), evenness (E) and by the number of IPs occupied by standing vegetation.

To assess the consistency within and the relationships between the initial major assemblages and between the derived clusters, we ordered the plots with canonical correspondence analysis (CCA) [33, 35, 36]. This method, where the ordination axes are constrained by linear functions of environmental variables, relates floristic composition to environmental variables listed in S1 Table. To test for the association between floristic and structural (growth-form) traits of our proposed classification schemes, we correlated both importance distance matrices with the Mantel test [37]. The standardized statistic (r) was randomized 1000 times to test for statistical significance.

### The environment

Climatic and edaphic variables (S1 Table) outline the major environmental differences across the rangelands and support our joint environment-floristics analysis. Climate data come from the WorldClim grid with 30” resolution [38]. The aridity index is the ratio of mean annual rainfall to pan or potential evaporation (ranges from 0 to 1; high to low aridity), was sourced from the Atlas of Living Australia [39, 40]. Soil texture, nutrient content, and water holding capacity for the top soil layer and landscape variables were obtained from the TERN’s Soil and Landscape Grid of Australia dataset at 30” resolution [41]. The totality of IPs covered by vegetation was employed as proxy for habitat suitability for plant growth. Original values of environmental variables were equalized by dividing them by their maximum value (all range between 0 and 1). Relationships among rescaled environment variables for plots was conveyed by principal component analysis (PCA) that orders the plots within multivariate environmental space. Also, differences between means of plots environmental variables within the major assemblages were statistically tested with ANOVA. All multivariate tasks were performed with PC-Ord V6.0 [42] and univariate statistical analysis with SYSTAT v10.0 [43].

## Results

**Fig 2:**
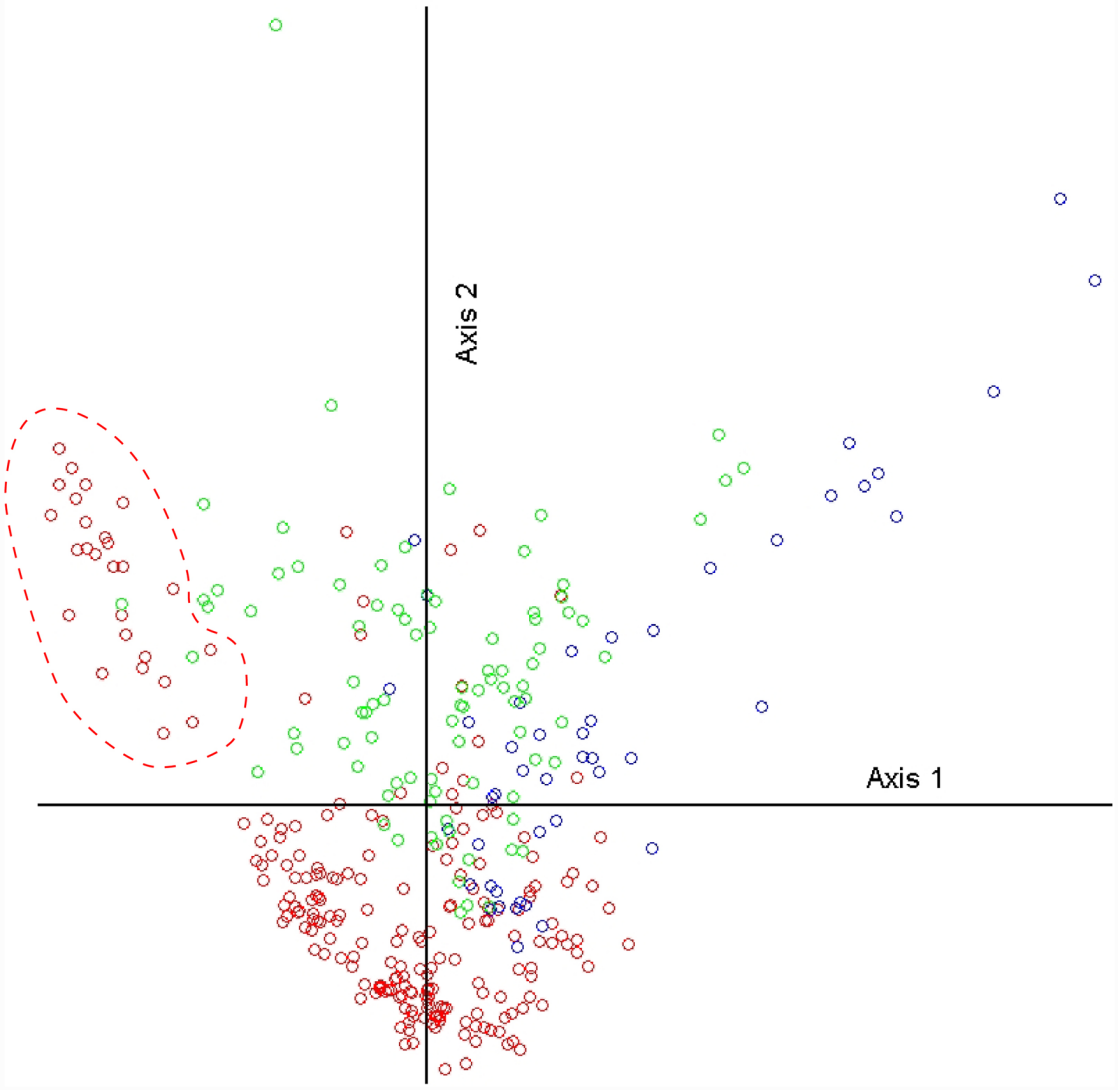
First two axes of the PCA ordination of sampled plots latitude within the environmental space. Plots from latitude -13°S to -18°S correspond to the savanna supercluster (green circles). Plots from latitude -19°S to -31°S correspond to the desert supercluster (red circles). Plots from latitude -32°S to -34°S correspond to the Mediterranean supercluster (blue circles). Variance explained was 52.9%. Desert plots at the upper left side of Axis 1 (encircled) are from the Mitchell Grass Downs (see below). Mediterranean plots spread at the upper right side are from the Flinders Lofty Block (see below).

The plot _x_ species matrix was sparse with 55.5% of species occurring in only one plot (singletons). Matrix size, sparsity and high species turnover (β-diversity = 84.8) prevented ecologically sound classification and ordination schemes. Deleting singleton species (those in only one plot) from analysis, did not decrease β-diversity substantially nor improve the interpretation of results. Consequently, we stratified the dataset according to the PCA ordination of plots’ latitude within the environmental space. Three latitudinally based plot groupings could be distinguished within the resulting ordination continuum: from -13°S to -18°S, from -19°S to -31°S and from -32°S to -34°S (Fig 2). This latitudinal sequence is approximately parallel to that of the Australian Bioclimatic Zones [44]. North to South, our groupings are: (1) Savannas, with a summer rain monsoonal climate; (2) Deserts, with arid or semiarid climate and; (3) Mediterranean, within a region with temperate climate and winter rains (Fig 1a and S1 Table). These groupings or “superclusters”, with substantially diminished β-diversity, were suitable for further detailed ecological analysis.

Axis 1 of the PCA captures most of the climatic differences among superclusters with the coolest and least arid Mediterranean plots at one end of this axis. At opposite ends of Axis 2 are desert and savanna plots that differ in soil fertility and texture variables (Fig 2 and Table 1). However, a group of Mitchell Grass Downs plots (bounded plots, Fig 2) included in the desert supercluster appear more related to savanna environment. The Mediterranean plots, with low aridity and temperature, high soil nitrogen and carbon content and high vegetated IPs, but low soil bulk density (Table 1), are widely spread along one end of Axis 1 probably due to the inclusion of a group of non-strictly rangeland plots from the Flinders-Lofty Block (Fig 2).

**Table 1:** Pearson correlation coefficients of environmental variables plot position along Axes 1 and 2 of the PCA ordination.

ENVIRONMENTAL VARIABLES	PCA AXIS 1		PCA AXIS 2	
	r	r^2^	r	r^2^
**ARIDITY INDEX**	**-0.667**	0.445	-0.267	0.071
RAIN SEASONALITY	0.294	0.086	-0.298	0.089
MAP (mm)	-0.264	0.07	-0.330	0.109
**MAT (°C)**	**0.671**	0.45	-0.157	0.025
**PHOSPHORUS (%)**	0.101	0.01	**-0.663**	0.439
**NITROGEN (%)**	**-0.818**	0.67	-0.407	0.166
**CEC (mEq / 100g)**	0.411	0.169	**-0.675**	0.456
**CARBON (%)**	**-0.863**	0.745	-0.197	0.039
pH	0.016	0	-0.438	0.192
**AWC (%)**	**0.691**	0.478	-0.297	0.088
**SAND (%)**	-0.246	0.06	**0.872**	0.760
**BULKDENS. (g / cm**^**3**^**)**	**0.598**	0.358	**0.570**	0.325
**CLAY (%)**	0.266	0.071	**-0.878**	0.770
**VEGETATED SUBSTRATE (# IPs)**	**0.638**	0.407	-0.076	0.006

Highlighted in bold are variables with regression coefficients > 0.5. Variance explained by both axes is 52.9%. Aridity Index: represented in an inverse scale (high values indicate low aridity); Rain Seasonality: Coefficient of variation. MAP: Mean annual precipitation; MAT: Mean annual temperature; Nitrogen, Phosphorus and Carbon: Mass fraction of total in the soil by weight; CEC: Effective cation exchange capacity; AWC: Available water capacity; Clay & Sand percent in soil; Bare substrate: Number of PIs uncovered by vegetation.

The univariate environmental analysis of superclusters supports the PCA ordination: The Mediterranean plots are the most temperate and least arid (due to relatively low evaporation rate); those from the savannas are the rainiest and most seasonally predictable while the desert plots are the most arid (Table 2 and S1 Table). Soil fertility, as denoted by nitrogen content and cation exchange capacity is highest in the Mediterranean plots that also had the highest soil pH and carbon content whereas soil phosphorus content was similar among superclusters (Table 2 and S1 Table). In the Mediterranean plots soil water holding capacity was the highest and sand content was the lowest. Clay content and soil bulk density were highest in the savannas (Table 2 and S1 Table). The proportion of vegetated ground was lowest in the desert plots (Table 2).

**Table 2:** Mean and standard error of main abiotic variables in the three superclusters.

ENVIRONM. VARIABLE	DESERT	MEDITERRANEAN	SAVANNA	F	P
Aridity Index	0.09 ± 0.01b	0.35 ± 0.02 a	0.28 ± 0.01a	F_(2,348)_ = 107.9	<0.001
Rain Seasonal.	43.43 ± 1.03b	27.47 ± 2.23 c	112.15 ±1.59a	F_(2,348)_ = 767.2	<0.001
MAP (mm)	250.4 ± 10.4c	400.9 ± 22.6b	724.4 ± 16.2a	F_(2,348)_ = 300.8	<0.001
MAT (°C)	20.90 ± 0.13b	16.27 ± 0.29c	26.00 ± 0.20a	F_(2,348)_ = 409.1	<0.001
Phosphorus (%)	0.024 ± 0.001a	0.023 ± 0.002a	0.023 ± 0.001a	F_(2,348)_ = 0.107	0.899
Nitrogen (%)	0.041 ± 0.001c	0.094 ± 0.002a	0.056 ± 0.002b	F_(2,348)_ = 197.03	<0.001
CEC (meq/100g)	14.21 ± 0.49a	12.37 ± 1.06ab	10.63 ± 0.764b	F_(2,348)_ = 7.98	<0.001
Carbon (%)	0.75 ± 0.02c	1.53 ± 0.05a	0.98 ± 0.03b	F_(2,348)_ = 103.53	<0.001
pH (CaCl_2_)	6.08 ± 0.03b	6.49 ± 0.07a	5.46 ± 0.05c	F_(2,348)_ = 67.73	<0.001
AWC (%)	14.99 ± 0.09b	13.20 ± 0.21c	15.72 ± 0.15a	F_(2,348)_ = 46.30	<0.001
Sand (%)	69.66 ± 0.80a	72.90 ± 1.73a	65.96± 1.23b	F_(2,348)_ = 5.88	0.003
Clay (%)	18.50 ± 0.58ab	16.42 ± 1.26b	20.61 ± 0.90a	F_(2,348)_ = 3.86	0.022
Bulk Density (g/cm^3^)	1.45 ±0.003a	1.39 ± 0.007b	1.45 ± 0.005a	F_(2,348)_ = 33.23	<0.001
Vegetated Substrate (# IPs)	150.5±8.3b	246.4 ± 18.1a	226.0 ± 12.9a	F_(2,348)_ = 19.10	<0.001

Statistical differences were tested with one-way ANOVA showing F and P values. Different letters indicate statistical significant differences between means. The complete plot dataset is displayed in S1 Table. Soil values refer to the 0–5 cm depth. Variable units as in Table 1. Aridity Index: represented in an inverse scale (high values indicate low aridity); Rain Seasonality: Coefficient of variation. MAP: Mean annual precipitation; MAT: Mean annual temperature; Nitrogen, Phosphorus and Carbon: Mass fraction of total in the soil by weight; CEC: Effective cation exchange capacity; AWC: Available water capacity; Clay & Sand percent in soil; Vegetated substrate: Number of IPs covered by vegetation.

### Floristic and structural traits

**Fig 3:**
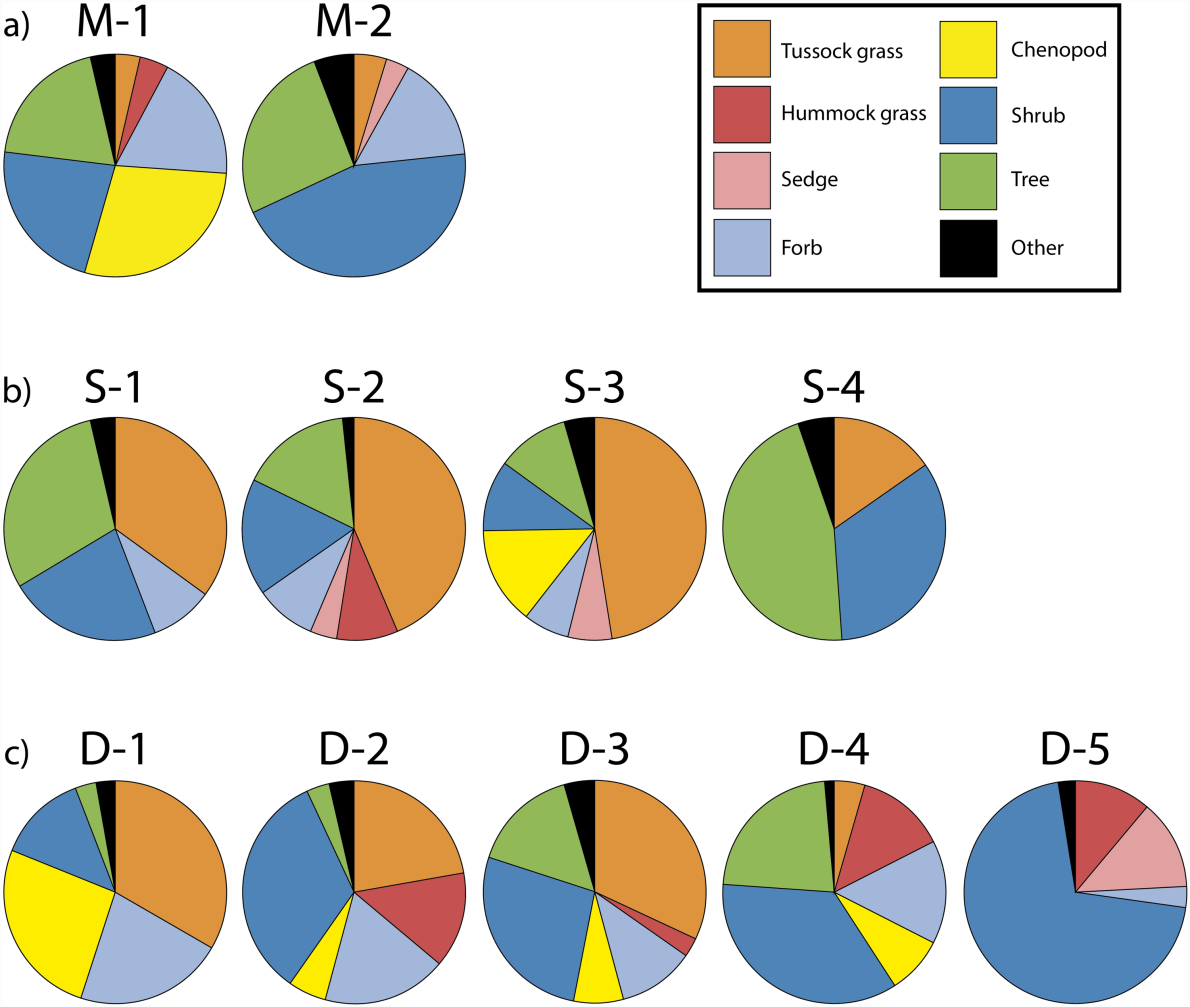
Growth form spectra. (a) Clusters M1 and M2 within the Mediterranean supercluster. (b) Clusters S1 to S4 within the savanna supercluster. (c) Clusters D1 to D5 within the desert supercluster. Area of the chart slices represent the proportional contribution of the importance of each growth form. Growth forms with IVIs less than 5% are grouped as “Other”. Shrub mallee and tree mallee growth forms are integrated into shrub and tree growth forms, respectively. Images showing the physiognomy of the most representative vegetation type are displayed in S4 File.

In terms of number of sampled plots, land area covered, species richness and β-diversity, floristic heterogeneity and structural complexity graded from relatively high in the deserts to relatively low in the Mediterranean supercluster (Table 3a). However, considering mean plot attributes, species richness was highest in the savannas and plot IPs were lowest in the deserts but other diversity indices did not differ among superclusters (Table 3a). Further clustering of the Mediterranean sites produced two distinct and relatively homogeneous vegetation types (Fig 1b). Although total species richness and β-diversity were similar, mean species richness, α-diversity and IPs per plot were significantly higher in cluster M2 (Table 3b). The high importance of chenopods (*Enchylaena spp*., *Sclerolaena spp*., *Maireana spp*.) and the presence of hummock grasses (*Triodia spp*.) in M1 is prominent (Fig 3a; S3 Table and S4 File) as well as the disjunction between eastern and western mallees (Fig 1b). Cluster M2 is woodier, with a clear dominance of open woodland and mallee eucalypts and shrubs with a characteristic signature provided by *Xanthorrhoea spp*. (Fig 3a; S3 Table and S4 File).

**Table 3:** Number of surveyed plots and floristic traits for (a) superclusters and (b-d) clusters within the three superclusters.

	*Plot #s*	*Species Richness (S)*	*β-Diversity*	MEAN TRAITS/PLOT			
				*Species Richness (S)*	*α-diversity (H)*	*Evenness (E)*	*IPs #*
(a) SUPERCLUSTERS							
MEDITERRANEAN	46	377	18.1	18.7^b^	1.78	0.61	246.4^a^
SAVANNA	90	618	26.3	21.6^a^	1.91	0.63	226.0^a^
DESERT	215	1014	49.8	18.9^b^	1.86	0.65	150.5^b^
F_**(2,365)**_	-	-	-	3.42	0.93	1.85	19.10
*P*	-	-	-	***0*.*03***	*0*.*39*	0.15	***<0*.*001***
CLUSTERS							
(b)MEDITERRANEAN							
M-1	32	214	4.5	16.1^b^	1.65^b^	0.59	191.2
M-2	14	199	3.7	25.0^a^	2.07^a^	0.65	372.5
F_**(1–44)**_	-	-	-	22.8	7.54	1.86	16.35
*P*	-	-	-	***<0*.*001***	***0*.*009***	*0*.*17*	***<0*.*001***
(c) SAVANNA							
S-1	11	194	6.3	27.5^a^	2.20	0.67	233.2
S-2	61	421	19.1	22.4^a^	1.92	0.63	231.0
S-3	10	73	5.5	12.2^b^	1.58	0.68	197.9
S-4	8	75	3.1	19.3^ab^	1.86	0.62	213.1
F_**(3–86)**_	-	-	-	6.25	2.05	0.77	0.25
*P*	-	-	-	***0*.*001***	*0*.*113*	*0*.*511*	*0*.*856*
(d) DESERT							
D-1	105	527	28.8	18.6^b^	1.94^ab^	0.68^c^	105.1^c^
D-2	54	374	20.0	18.8^b^	1.79^b^	0.61^b^	198.2^b^
D-3	35	192	13.5	14.2^c^	1.59^c^	0.62^ab^	143.2^c^
D-4	10	119	5.3	20.0^b^	1.86^ab^	0.63^ab^	224.0^b^
D-5	11	157	3.4	36.5^a^	2.36^a^	0.66^abc^	305.8^a^
F_**(4,210)**_	-	-	-	19.22	5.83	3.11	16.41
*P*	-	-	-	***<0*.*001***	***<0*.*001***	***0*.*016***	***<0*.*001***

For floristic traits and number of vegetated IPs, statistical significance was tested with one-way ANOVAs and the Bonferroni post-hoc test. Different superscript letters represent significant statistical differences.

The savanna supercluster was considerably larger and more complex (Fig 1c). Here, four clusters can be distinguished floristically and structurally (Table 3c and Fig 3b). Clusters S1 and S2 were the most species rich with S2 displaying the highest β-diversity. However, mean α-diversity and evenness were similar among clusters (Table 3c). Clusters S1 and S2 also had the highest mean species richness and IP cover per plot due to the stratified nature of its woody component (mostly *Eucalyptus spp*.) (Fig 3c; S4 Table and S4 File). Cluster S1 also shows an important floristic disjunction associated to the Mitchell Grass Downs area mentioned above. The interaction between tussock grasses and trees defines the physiognomy of the clusters, which grades from the densely wooded S4, strongly dominated by *Melaleuca citrolens* (Myrtaceae) to shared tree-grass dominance to largely treeless landscapes in S3 (Fig 3b; S4 Table and S4 File). Although tussock grasses prevail, clusters S2 and S3 show more structural diversity and differ by the relative importance of hummock grasses and chenopods (Fig 3b and S4 File).

The desert supercluster was the largest and consequently the most heterogeneous and complex (Fig 1d). Five clusters could be distinguished with significant differences in all floristic traits at the mean plot level (Table 3d). Cluster D5 had the highest mean number of species per plot and α-diversity although it was not the most species rich overall (Table 3d). Cluster D5 also had the highest proportion of vegetation cover, mostly by shrubs and sedges (Table 3d and Fig 3c). Tussock and hummock grasses (mostly *Triodia* spp.) share dominance with shrubs and trees and define the physiognomy of the clusters with chenopods being important in D1 (Fig 3c; S5 Table and S4 File). Invasive buffel grass (*Cenchrus ciliaris*) dominates the herbaceous stratum in cluster D3 while mulga (*Acacia aneura* complex) predominates among shrubs (S5 Table and S4 File). Cluster D2 displays an important east-west disjunction. For all superclusters, the correspondence between the floristic (Sørensen dissimilarity) and the structural or growth form (Euclidean distance) matrices was statistically significant (Table 4) which supports our proposed supercluster classification scheme.

**Table 4:** Mantel test between floristic and growth form distance matrices.

Supercluster	Standardized Mantel Statistic (r)	p
Savanna	0.126	0.038
Desert	0.355	0.001
Mediterranean	0.212	0.001

Standardized Mantel statistic (r), randomized 1000 times and the resulting p-value of the association between floristic (Sørensen) distance among plots and the growth form (Euclidean) distance among plots.

### Constrained ordination

**Fig 4:**
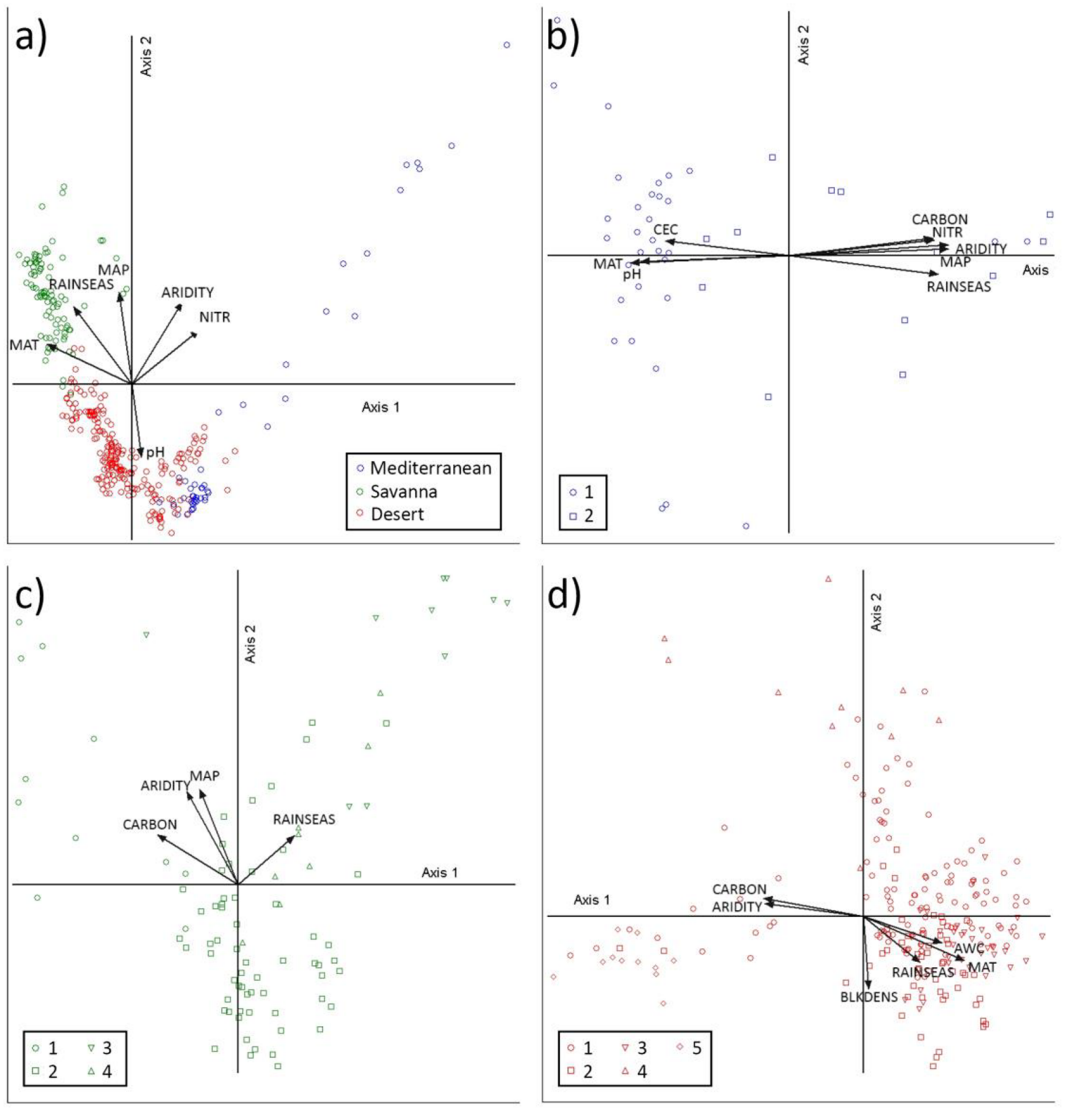
Biplots of the first two axes of the CCA ordination. (a) All plots segregated by supercluster. Variance explained by Axes 1 and 2 = 0.9% and 0.9%. (b) Mediterranean supercluster showing clusters M1 and M2. Variance explained by Axes 1 and 2 = 4.2% and 3.8%. (c) Savanna supercluster displaying clusters S1 to S4. Variance explained by Axes 1 and 2 = 3.0% and 2.8%. (d) Desert supercluster segregated by clusters D1 to D5. Variance explained by Axes 1 and 2 = 1.5% and 1.4%. Arrows represent the most important environmental variables correlated with the plot ordination. Length of arrow is related to their correlation coefficient with ordination axes and is shown in S6 Table.

The CCA ordination shows an appreciable demarcation between superclusters and, except for the Mediterranean supercluster, they exhibit relatively compact grouping (Fig 4a). High MAT, MAP and rainfall seasonality define the savannas, while low aridity but high soil nitrogen and carbon content are correlated with Mediterranean plots (Fig 4a and S6 Table). High aridity and low soil N content plus alkaline soils define the desert supercluster (Fig 4a and S6 Table). The soil physical properties and the proportion of vegetation covered soil played a relatively minor role in this ordination scheme (S6 Table). The two Mediterranean clusters are clearly defined by floristic and environmental contrasts where plots from cluster M1 are found under the most arid and warm climate on relatively unfertile, acidic and clayey soils (Fig 4b and S6 Table). The ordination of the savannas is more complex, showing a central cluster, S2 (that embraces most of S4), and the satellite clusters S1 and S3 (Fig 4c). Aridity is the main environmental variable that splits the drier and hotter cluster S2 from the rest. Soil fertility and soil texture had only minor roles in this ordination scheme (Fig 4c and S6 Table). Clusters S3 and S4 appear to be floristic variants of S2 (Fig 4c) as described above. The ordination of the desert supercluster generated the most complex CCA biplot (Fig 4d). Here, aridity, MAT and rainfall seasonality join soil carbon and available water content and bulk density as the main environmental correlates to the ordination (Fig 4d and S6 Table). Clusters D1, D2 and D3 constitute the nucleus of the ordination with D4 and D5 becoming satellites (Fig 4d). The dispersion of plots from cluster D2 is related to the disjunction described above.

## Discussion

A large and sparse dataset with high species turnover or β-diversity prevented initial attempts to obtain ecologically sound classification and ordination outcomes. Consequently, we ordinated plots latitude within the environmental space to segment our data set to obtain three coherent major groups or “superclusters”. This environmentally based segmentation, is close to that of the Australian Agroclimatic Zones [44] for which reason we consider it adequate. Further clustering was necessary to obtain ecologically interpretable groups, which were tested and supported by the coherence and agreement between their respective environment, floristic composition and structure. The resulting number of derived clusters was linked to the species richness and β-diversity of individual superclusters.

Currently, our results are influenced by sparse and still incomplete sampling over large areas and the uneven number of plots in each supercluster. However, there are now >500 plots being analysed that will fill gaps in sampling density and distribution. Therefore, our proposed classificatory scheme and floristic-environmental correlations are subject to refinement through further analysis and interpretation. Nevertheless, this paper provides a meaningful and valuable contribution to our understanding of Australian vegetation community distributions and the significance of standardized survey methods. Also, our results will help to determine where further survey effort is needed [32].

### Floristic and structural diversity

While many vegetation studies report floristic composition and vegetation structure, very few associate these results numerically [45]. The significant correlation between our floristic and structural (growth form) importance-based distance matrices supports our proposed initial dataset supercluster segmentation. Additional surveying and insertion of new plots within the classificatory scheme proposed here, will further test its adequacy. Since biodiversity comparisons are contingent on area sampled and survey density, the desert supercluster was the most speciose. At the plot level however, species richness and diversity differences are less well defined but there are remarkable structural differences in terms of the relative importance of plant growth-forms.

The floristic ordination of the Mediterranean plots was the most scattered due to the prominent spatial disjunction discussed above and the inclusion of plots from Flinders Lofty Block, which is considered to be a floristic refugium [4]. The two Mediterranean clusters differ significantly in mean species richness, α-diversity and plot cover, the proportion of shrubs and the presence and importance of chenopods and hummock grasses. The prominent disjunction in the mallee type vegetation in cluster M1, floristically splitting eastern and western sites, is likely both an artefact of high spatial isolation between sampling locations and a result of broader east-west biogeographic influences. Historical marine transgressions into the Nullarbor Plain related to sea level rise in the Great Australian Blight, and ensuing calcium enrichment of soils, generated significant barriers to vegetation in concert with high aridity [46].

Within the savannas, cluster S2 was the most sampled and consequently the most species rich. However, the highest mean plot species richness was found in the much smaller cluster S1 that displays an important floristically based disjunction which is related to the Mitchell Grass Downs. Cluster S1 appears to be a transition or borderline area between desert and savanna. Floristically, the desert-savanna floristic transition is possibly caused by shifting the dominance of *Eucalyptus* species from *E*. *tetrodonta* and *E*. *tectifica* in the Northern Territory to *E*. *crebra* and *E*. *similis* in Queensland, although many of the grass species are the same, with a preponderance of *Astrebla* spp. The appropriate interpretation of the environmental and floristic ordinations of plots from cluster S1 and the Mitchell Grass Downs is uncertain and open to analysis and discussion [47]. The sites comprising cluster S3 are on the extensive floodplains of the Gulf of Carpentaria with highly saline soils, low α-diversity and strong dominance of *Sporobulus virginicus* (salt couch grass). The plots of the other small and highly wooded cluster S4 display a very strong dominance by *Melaleuca citrolens* (Myrtaceae) and *Lysiphyllum cunninghammi* (Fabaceae) which are distinctive of poorly drained and seasonally flooded soils [48].

The great number of sampled sites and the environmental heterogeneity of the desert supercluster, discussed above, are direct causes of its high β-diversity. Much of inland Australia is covered by tussock and hummock grasslands and scattered woodlands. However, large expanses such as the northern Great Victoria and Gibson deserts, north-eastern South Australia and the area stretching from south of Longreach to the New South Wales border are still unsampled. They will likely be future target areas, in part due to the results reported here. The prominent disjunction within cluster D2 is consequence of this sampling gap. The potential effect of the invasive buffel grass in reducing species richness and diversity was evident in cluster D3, where buffel reached the highest importance value while mean species richness and diversity were the lowest among the desert clusters. The small cluster D5 merits special attention for the relatively high proportion of sedges (*Lepidobulus preissianus* and *Lepidosperma sanguinolentum*) within a thick mallee shrub and for displaying high species richness. The ecological importance of these dryland sedges is discussed by [49] and the high species richness and diversity of this cluster is due to its closeness to one of the Australian biodiversity hotspots [50].

Although not the purpose of this study, the comparison between our floristically based classification scheme and that of the Interim Bioregionalization of Australia (IBRA) [51] is unavoidable. Fifty-two IBRA bioregions have been recognized in the rangelands [28]. Our dataset covers 38 bioregions, which are closely associated with our clusters. The 38 surveyed bioregions were grouped into our 11 clusters (2 Mediterranean, 4 savannas and 5 deserts). Our largest cluster (D1 with 105 plots) covers 24 bioregions and the smallest cluster (S4 with 8 plots) corresponds to only one bioregion. Although they are not strictly comparable, our floristically based classification scheme provides an alternative, potentially corroborative framework for bioregional mapping of the rangelands.

### Vegetation—Environment relationships

The vast environmental heterogeneity of the rangelands upholds the floristic and structural complexity of its vegetation. Ecological theory predicts that environmental heterogeneity should be positively correlated to floristic diversity under comparable sampling efforts. We did not find this correlation due to unequal cluster sampling. However, for each supercluster, the dispersion of plots in environmental and floristic ordination space, is relatively alike and reasonably supports the environment-diversity prediction. For example, the wide dispersion of the plots within the Mediterranean and desert superclusters was correlated with relatively large climatic and edaphic gradients while the relative uniformity of the savanna landscape climate is associated to a more compact plot ordination.

Our uni- and multivariate analyses show that aridity is the major environmental variable splitting savannas, deserts and Mediterranean biomes and offer quantitative support to previous descriptions [8, 9, 52–55]. However, floristic-environment correlations are subject to the idiosyncrasies of each supercluster. Thus, although aridity was a prominent predictor of composition in all of them, air temperature was an important variable in desert and Mediterranean superclusters but not in the uniformly warm savannas. The effects of rainfall amount and seasonality interact with temperature to distinguish the climate of savannas with summer monsoonal rains from the Mediterranean region, seasonally temperate and with winter rains. The deserts are uniformly arid or semi-arid where temperature fluctuations are more drastic on a daily basis. In consequence, vegetation is more constrained by drought in the deserts and less in the savannas whereas temperature is more limiting in the Mediterranean temperate regions.

At the scale of our analysis, soils appear to be more fertile in the Mediterranean supercluster as consequence of higher total nitrogen, cation exchange capacity and carbon content. Soil phosphorus is much less relevant and did not influence our supercluster ordination. The general low phosphorus availability in the continent is a recognized limitation [9, 56]. Within each supercluster though, even minor variances in soil nutrient content were important in defining relationships among clusters. Other traits, such as soil pH, demarcate two Mediterranean clusters whereas salinity, poor drainage and seasonal flooding delineate two savanna clusters. At present, soil data were interpolated with the associated limitations but future work on analysing the collected soil samples will refine our results.

Although not considered here, past fires influence vegetation and their subsequent effect likely confound our analysis. Caused by differences in vegetation cover and structure, it is established that savannas are prone to more frequent but less intense fires than the wooded Mediterranean sites and that deserts are least affected due to their sparse vegetation cover [9, 14, 57, 58]. The legacy effects of grazing on rangeland vegetation remains contentious [59, 60] but would be more pronounced in areas of low productivity (arid to semi-arid) due to higher sensitivity to disturbances. The prominent invasion of buffel grass in some areas of the desert has been related to decreased vegetation diversity by outcompeting native species or through the fire-invasion feedback [60–62]. Alternatively, buffel grass predominance could be attributed to preferential invasion into low diversity or disturbed sites.

## Conclusions

- We present an integrated and comparative environmental, floristic and structural description of rangeland vegetation based on standardised and quantitative vegetation surveys that encompasses most of Australian rangeland jurisdictions.
- Our results offer a tentative classification scheme that is novel, ecologically sound and coherent in terms of floristic composition and structural attributes. Furthermore, our floristically based classificatory framework, conveyed as clusters within superclusters, is supported by environmental and structural growth-form analyses.
- Differences in area and in surveying intensity, between and within our proposed groupings, the hidden effects of paleo-climatic events, fire, grazing and invasion by non-native plants may all influence comparisons or account for some of the unexplained variance in vegetation attributes.
- As more sites are surveyed with the same methodology, an upgraded rangeland vegetation classification scheme and more refined floristic-environment relationships will be accessible.

## Supporting information

S1 Table AusPlots listing. Segregated by superclusters and clusters Includes geographical coordinates, environmental variables, species richness and diversity indices.
(XLSX)

S2 Table List of species with respective growth forms surveyed for this study. Identification and taxonomy follows the Australian Plant Name Index (http://www.anbg.gov.au/apni/).
(DOCX)

S3 Table Index of importance value (IVI) for the most important species within each growth form in the Mediterranean supercluster. (XLSX)

S4 Table Index of importance value (IVI) for the most important species within each growth form in the savanna supercluster. (XLSX)

S5 Table Index of importance value (IVI) for the most important species within each growth form in the desert supercluster. (XLSX)

S6 Table Pearson correlation coefficients between Axes 1 and 2 of the CCA ordination scores and principal environmental variables. The entire data set and for each supercluster. Correlation coefficients > 0.700 are highlighted.
(DOCX)

S1 File Plot x species matrix for the Mediterranean supercluster. Numbers in cells represent intercept points (IP). Species name key in S2 Table.
(XLSX)

S2 File Plot x species matrix for the savanna supercluster. Numbers in cells represent intercept points (IP). Species name key in S2 Table.
(XLSX)

S3 File Plot x species matrix for the desert supercluster. Numbers in cells represent intercept points (IP). Species name key in S2 Table.
(XLSX)

S4 File Photographs of the most representative vegetation landscape of each cluster. Mediterranean supercluster (clusters M1-M2); Savanna supercluster (clusters S1-S4); Desert supercluster (clusters D1-D5). Plot provenance is also displayed.
(ZIP)
