# Supplementary material for: Floristic and structural assessment of Australian rangeland vegetation with standardized plot-based surveys
Source: PLoS One. 2018 Sep 7;13(9):e0202073. doi: 10.1371/journal.pone.0202073 (PMC6128463; doi:10.1371/journal.pone.0202073)
Supplement: S2 Table — Identification and taxonomy follows the Australian Plant Name Index (http://www.anbg.gov.au/apni/). (DOCX) [file pone.0202073.s002.docx]

|  | S2 Table - Species & Growth Forms |  |
| --- | --- | --- |
|  | SPECIES | GROWTH FORM |
|  |  |  |
| 1 | Abutilon fraseri | Forb |
| 2 | Abutilon halophilum | Forb |
| 3 | Abutilon hannii | Forb |
| 4 | Abutilon leucopetalum | Forb |
| 5 | Abutilon otocarpum | Forb |
| 6 | Abutilon oxycarpum | Forb |
| 7 | Abutilon sp. | Forb |
| 8 | Acacia aneura | Shrub |
| 9 | Acacia acuminata | Shrub |
| 10 | Acacia adoxa | Shrub |
| 11 | Acacia alleniana | Shrub |
| 12 | Acacia anceps | Shrub |
| 13 | Acacia ancistrocarpa | Shrub |
| 14 | Acacia aptaneura | Shrub |
| 15 | Acacia araneosa | Shrub |
| 16 | Acacia asperulacea | Shrub |
| 17 | Acacia ayersiana | Shrub |
| 18 | Acacia beauverdiana | Shrub |
| 19 | Acacia bivenosa | Shrub |
| 20 | Acacia burkittii | Shrub |
| 21 | Acacia caesaneura | Shrub |
| 22 | Acacia cambagei | Shrub |
| 23 | Acacia catenulata | Shrub |
| 24 | Acacia cochlearis | Shrub |
| 25 | Acacia colei var. colei | Shrub |
| 26 | Acacia colletioides | Shrub |
| 27 | Acacia consanguinea | Shrub |
| 28 | Acacia continua | Shrub |
| 29 | Acacia contriva | Shrub |
| 30 | Acacia coolgardiensis | Shrub |
| 31 | Acacia cupularis | Shrub |
| 32 | Acacia cyclops | Shrub |
| 33 | Acacia cylindrica | Shrub |
| 34 | Acacia desertorum var. nudipes | Shrub |
| 35 | Acacia dictyophleba | Shrub |
| 36 | Acacia difficilis | Shrub |
| 37 | Acacia dimidiata | Shrub |
| 38 | Acacia duriuscula | Shrub |
| 39 | Acacia effusifolia | Shrub |
| 40 | Acacia enervia subsp. enervia | Shrub |
| 41 | Acacia erinacea | Shrub |
| 42 | Acacia estrophiolata | Shrub |
| 43 | Acacia euthycarpa | Shrub |
| 44 | Acacia excelsa subsp. excelsa | Tree |
| 45 | Acacia galioides | Shrub |
| 46 | Acacia georginae | Shrub |
| 47 | Acacia gibbosa | Shrub |
| 48 | Acacia gonocarpa | Shrub |
| 49 | Acacia hammondii | Shrub |
| 50 | Acacia harpophylla | Tree |
| 51 | Acacia havilandiorum | Shrub |
| 52 | Acacia hemignosta | Shrub |
| 53 | Acacia hemiteles | Shrub |
| 54 | Acacia hemsleyi | Shrub |
| 55 | Acacia hilliana | Shrub |
| 56 | Acacia holosericea | Shrub |
| 57 | Acacia inaequiloba | Shrub |
| 58 | Acacia incurvaneura | Shrub |
| 59 | Acacia iteaphylla | Shrub |
| 60 | Acacia jamesiana | Shrub |
| 61 | Acacia jennerae | Shrub |
| 62 | Acacia julifera subsp. julifera | Shrub |
| 63 | Acacia kempeana | Shrub |
| 64 | Acacia lamprocarpa | Shrub |
| 65 | Acacia lasiocalyx | Shrub |
| 66 | Acacia ligulata | Shrub |
| 67 | Acacia limbata | Shrub |
| 68 | Acacia longispinea | Shrub |
| 69 | Acacia lutescens | Shrub |
| 70 | Acacia lycopodiifolia | Shrub |
| 71 | Acacia lysiphloia | Shrub |
| 72 | Acacia maitlandii | Shrub |
| 73 | Acacia melleodora | Shrub |
| 74 | Acacia merrallii | Shrub |
| 75 | Acacia mimula | Shrub |
| 76 | Acacia monticola | Shrub |
| 77 | Acacia murrayana | Shrub |
| 78 | Acacia myrtifolia | Shrub |
| 79 | Acacia neurocarpa | Shrub |
| 80 | Acacia nyssophylla | Shrub |
| 81 | Acacia orthotricha | Shrub |
| 82 | Acacia oswaldii | Shrub |
| 83 | Acacia pachyacra | Shrub |
| 84 | Acacia papyrocarpa | Shrub |
| 85 | Acacia paradoxa | Shrub |
| 86 | Acacia paraneura | Shrub |
| 87 | Acacia phlebocarpa | Shrub |
| 88 | Acacia platycarpa | Shrub |
| 89 | Acacia plectocarpa subsp. tanumbirinensis | Shrub |
| 90 | Acacia prainii | Shrub |
| 91 | Acacia pycnantha | Shrub |
| 92 | Acacia resinimarginea | Shrub |
| 93 | Acacia rhodophloia | Shrub |
| 94 | Acacia rivalis | Shrub |
| 95 | Acacia rothii | Shrub |
| 96 | Acacia sericophylla | Shrub |
| 97 | Acacia shirleyi | Tree |
| 98 | Acacia sibina | Shrub |
| 99 | Acacia sibirica | Shrub |
| 100 | Acacia sp. | Shrub |
| 101 | Acacia stenophylla | Shrub |
| 102 | Acacia stereophylla subsp. stereophylla | Shrub |
| 103 | Acacia stipuligera | Shrub |
| 104 | Acacia subternata | Shrub |
| 105 | Acacia tenuissima | Shrub |
| 106 | Acacia tephrina | Tree |
| 107 | Acacia tetragonophylla | Shrub |
| 108 | Acacia thomsonii | Shrub |
| 109 | Acacia torulosa | Shrub |
| 110 | Acacia uncinella | Shrub |
| 111 | Acacia victoriae | Shrub |
| 112 | Acacia victoriae subsp. victoriae | Shrub |
| 113 | Acacia victoriae subsp. arida | Shrub |
| 114 | Acacia wanyu | Shrub |
| 115 | Acacia wattsiana | Shrub |
| 116 | Acacia wickhamii var. viscidula | Shrub |
| 117 | Acacia yorkrakinensis subsp. acrita | Shrub |
| 118 | Achyranthes aspera | Forb |
| 119 | Acrotriche fasciculiflora | Shrub |
| 120 | Acrotriche affinis | Shrub |
| 121 | Acrotriche depressa | Shrub |
| 122 | Acrotriche fasciculiflora | Shrub |
| 123 | Acrotriche serrulata | Shrub |
| 124 | Actinobole uliginosa | Forb |
| 125 | Adriana tomentosa var. hookeri | Forb |
| 126 | Aerva javanica | Forb |
| 127 | Aeschynomene indica | Forb |
| 128 | Ajuga iva | Forb |
| 129 | Albizia lebbeck | Tree |
| 130 | Alectryon oleifolius | Shrub |
| 131 | Alectryon oleifolius subsp. canescens | Shrub |
| 132 | Allocasuarina campestris | Shrub |
| 133 | Allocasuarina corniculata | Shrub |
| 134 | Allocasuarina decaisneana | Tree |
| 135 | Allocasuarina muelleriana | Shrub |
| 136 | Allocasuarina muelleriana subsp. muelleriana | Shrub |
| 137 | Allocasuarina spinosissima | Shrub |
| 138 | Allocasuarina striata | Tree |
| 139 | Allocasuarina verticillata | Shrub |
| 140 | Allopterigeron filifolius | Forb |
| 141 | Alloteropsis semialata | Tussock grass |
| 142 | Alphitonia sp. | Tree |
| 143 | Alphitonia excelsa | Tree |
| 144 | Alternanthera angustifolia | Forb |
| 145 | Alternanthera denticulata | Forb |
| 146 | Alternanthera nana | Forb |
| 147 | Alternanthera nodiflora | Forb |
| 148 | Aluta maisonneuvei | Forb |
| 149 | Aluta maisonneuvei subsp. maisonneuvei | Shrub |
| 150 | Alyogyne pinoniana | Shrub |
| 151 | Alyxia buxifolia | Shrub |
| 152 | Amaranthus mitchellii | Forb |
| 153 | Ampelocissus frutescens | Shrub |
| 154 | Amphibromus nervosus | Tussock grass |
| 155 | Amphipogon caricinus var. caricinus | Shrub |
| 156 | Amyema maidenii | Epiphyte |
| 157 | Amyema miquelii | Epiphyte |
| 158 | Amyema miraculosa subsp. boormanii | Epiphyte |
| 159 | Amyema quandang | Epiphyte |
| 160 | Amyema villiflora subsp. tomentilla | Epiphyte |
| 161 | Anagallis arvensis | Forb |
| 162 | Androcalva loxophylla | Shrub |
| 163 | Angianthus conocephalus | Shrub |
| 164 | Angianthus tomentosus | Forb |
| 165 | Anisomeles inodora | Forb |
| 166 | Annual tussock grass | Tussock grass |
| 167 | Antidesma ghaesembilla | Shrub |
| 168 | Aphyllodium biarticulatum | Shrub |
| 169 | Apophyllum anomalum | Shrub |
| 170 | Archidendropsis basaltica | Tree |
| 171 | Aristida anthoxanthoides | Tussock grass |
| 172 | Aristida arida | Tussock grass |
| 173 | Aristida calycina | Tussock grass |
| 174 | Aristida contorta | Tussock grass |
| 175 | Aristida dominii | Tussock grass |
| 176 | Aristida holathera | Tussock grass |
| 177 | Aristida hygrometrica | Tussock grass |
| 178 | Aristida inaequiglumis | Tussock grass |
| 179 | Aristida ingrata | Tussock grass |
| 180 | Aristida jerichoensis | Tussock grass |
| 181 | Aristida latifolia | Tussock grass |
| 182 | Aristida nitidula | Tussock grass |
| 183 | Aristida obscura | Tussock grass |
| 184 | Aristida perniciosa | Tussock grass |
| 185 | Aristida pruinosa | Tussock grass |
| 186 | Aristida sp. | Tussock grass |
| 187 | Aristida utilis | Tussock grass |
| 188 | Arundinella nepalensis | Tussock grass |
| 189 | Arundinella setosa | Tussock grass |
| 190 | Asparagus asparagoides | Forb |
| 191 | Asphodelus fistulosus | Forb |
| 192 | Asteraceae sp. | Forb |
| 193 | Asteromyrtus symphyocarpa | Shrub |
| 194 | Astrebla elymoides | Tussock grass |
| 195 | Astrebla lappacea | Tussock grass |
| 196 | Astrebla pectinata | Tussock grass |
| 197 | Astrebla sp. | Tussock grass |
| 198 | Astrebla squarrosa | Tussock grass |
| 199 | Astroloma conostephioides | Shrub |
| 200 | Astroloma humifusum | Shrub |
| 201 | Atalaya hemiglauca | Shrub |
| 202 | Atalaya variifolia | Shrub |
| 203 | Atriplex acutibractea subsp. acutibractea | Shrub |
| 204 | Atriplex angulata | Chenopod |
| 205 | Atriplex cinerea | Chenopod |
| 206 | Atriplex elachophylla | Chenopod |
| 207 | Atriplex holocarpa | Chenopod |
| 208 | Atriplex incrassata | Chenopod |
| 209 | Atriplex infrequens | Chenopod |
| 210 | Atriplex leptocarpa | Chenopod |
| 211 | Atriplex limbata | Chenopod |
| 212 | Atriplex lindleyi lindleyi | Chenopod |
| 213 | Atriplex muelleri | Chenopod |
| 214 | Atriplex nana | Chenopod |
| 215 | Atriplex nummularia | Chenopod |
| 216 | Atriplex nummularia subsp. nummularia | Chenopod |
| 217 | Atriplex nummularia subsp. spathulata | Chenopod |
| 218 | Atriplex paludosa subsp. cordata | Chenopod |
| 219 | Atriplex rhagodioides | Chenopod |
| 220 | Atriplex sp. | Chenopod |
| 221 | Atriplex spongiosa | Chenopod |
| 222 | Atriplex stipitata | Chenopod |
| 223 | Atriplex turbinata | Chenopod |
| 224 | Atriplex velutinella | Chenopod |
| 225 | Atriplex vesicaria | Chenopod |
| 226 | Atriplex vesicaria subsp. calcicola | Chenopod |
| 227 | Atriplex vesicaria subsp. vesicaria | Chenopod |
| 228 | Austrostipa acrociliata | Tussock grass |
| 229 | Austrostipa drummondii | Tussock grass |
| 230 | Austrostipa elegantissima | Tussock grass |
| 231 | Austrostipa eremophila | Tussock grass |
| 232 | Austrostipa hemipogon | Tussock grass |
| 233 | Austrostipa lanata | Tussock grass |
| 234 | Austrostipa mollis | Tussock grass |
| 235 | Austrostipa nitida | Tussock grass |
| 236 | Austrostipa nodosa | Tussock grass |
| 237 | Austrostipa nullanulla | Tussock grass |
| 238 | Austrostipa plumigera | Tussock grass |
| 239 | Austrostipa puberula | Tussock grass |
| 240 | Austrostipa scabra | Tussock grass |
| 241 | Austrostipa scabra subsp. scabra | Tussock grass |
| 242 | Austrostipa scabra subsp. falcata | Tussock grass |
| 243 | Austrostipa sp. | Tussock grass |
| 244 | Austrostipa trichophylla | Tussock grass |
| 245 | Avena barbata | Tussock grass |
| 246 | Azadirachta indica | Shrub |
| 247 | Babingtonia behrii | Shrub |
| 248 | Baeckea crispiflora | Shrub |
| 249 | Baeckea elderiana | Shrub |
| 250 | Baeckea muricata | Shrub |
| 251 | Balaustion pulcherrimum | Forb |
| 252 | Banksia elderiana | Shrub |
| 253 | Banksia marginata | Shrub |
| 254 | Basilicum polystachyon | Forb |
| 255 | Batis argillicola | Forb |
| 256 | Bauhinia cunninghamii | Shrub |
| 257 | Bauhinia cunninghamii subsp. gilva | Shrub |
| 258 | Beaufortia interstans | Shrub |
| 259 | Bergia henshallii | Shrub |
| 260 | Bergia trimera | Forb |
| 261 | Beyeria opaca | Shrub |
| 262 | Beyeria sulcata var. sulcata | Forb |
| 263 | Bidens bipinnata | Forb |
| 264 | Billardiera uniflora | Forb |
| 265 | Boerhavia coccinea | Forb |
| 266 | Boerhavia dominii | Forb |
| 267 | Boerhavia paludosa | Forb |
| 268 | Boerhavia pubescens | Forb |
| 269 | Boerhavia repleta | Forb |
| 270 | Boerhavia schomburgkiana | Forb |
| 271 | Boerhavia sp. | Forb |
| 272 | Bonamia breviflora | Forb |
| 273 | Bonamia erecta | Shrub |
| 274 | Bonamia media | Forb |
| 275 | Boronia coerulescens | Shrub |
| 276 | Boronia coerulescens subsp. spinescens | Shrub |
| 277 | Boronia lanceolata | Shrub |
| 278 | Boronia ternata var. ternate | Shrub |
| 279 | Borya constricta | Sedge |
| 280 | Bossiaea bossiaeoides | Shrub |
| 281 | Bossiaea prostrata | Shrub |
| 282 | Bothriochloa bladhii | Tussock grass |
| 283 | Bothriochloa ewartiana | Tussock grass |
| 284 | Bothriochloa macra | Tussock grass |
| 285 | Bothriochloa pertusa | Tussock grass |
| 286 | Brachyachne convergens | Tussock grass |
| 287 | Brachyachne tenella | Tussock grass |
| 288 | Brachychiton diversifolius | Shrub |
| 289 | Brachychiton diversifolius subsp. diversifolius | Shrub |
| 290 | Brachychiton megaphyllus | Tree |
| 291 | Brachychiton populneus subsp. trilobus | Tree |
| 292 | Brachyscome ciliaris | Forb |
| 293 | Brachyscome iberidifolia | Forb |
| 294 | Brachyscome lineariloba | Forb |
| 295 | Brachyscome sp. | Forb |
| 296 | Brassica juncea | Forb |
| 297 | Brassica sp. | Forb |
| 298 | Brassica tournefortii | Forb |
| 299 | Brassicaceae sp. | Forb |
| 300 | Breynia oblongifolia | Shrub |
| 301 | Briza maxima | Tussock grass |
| 302 | Bromus diandrus | Tussock grass |
| 303 | Brunonia australis | Forb |
| 304 | Buchanania obovata | Shrub |
| 305 | Bulbine alata | Forb |
| 306 | Bulbine bulbosa | Forb |
| 307 | Bulbostylis barbata | Sedge |
| 308 | Burchardia umbellata | Forb |
| 309 | Bursaria spinosa subsp. lasiophylla | Forb |
| 310 | Bursaria spinosa subsp. spinosa | Shrub |
| 311 | Caesia calliantha | Forb |
| 312 | Calandrinia porifera | Forb |
| 313 | Calandrinia balonensis | Forb |
| 314 | Calandrinia eremaea | Forb |
| 315 | Calandrinia uniflora | Forb |
| 316 | Callistemon phoeniceus | Shrub |
| 317 | Callitris columellaris | Tree |
| 318 | Callitris glaucophylla | Tree |
| 319 | Callitris gracilis | Tree |
| 320 | Callitris intratropica | Shrub |
| 321 | Callitris preissii | Shrub |
| 322 | Callitris rhomboidea | Tree |
| 323 | Callitris verrucosa | Tree |
| 324 | Calocephalus platycephalus | Forb |
| 325 | Calocephalus sp. | Forb |
| 326 | Calothamnus gilesii | Shrub |
| 327 | Calotis breviradiata | Shrub |
| 328 | Calotis cymbacantha | Forb |
| 329 | Calotis erinacea var. erinacea | Forb |
| 330 | Calotis hispidula | Forb |
| 331 | Calotis multicaulis | Forb |
| 332 | Calotis plumulifera | Forb |
| 333 | Calytrix birdii | Shrub |
| 334 | Calytrix breviseta subsp. stipulosa | Shrub |
| 335 | Calytrix brownii | Shrub |
| 336 | Calytrix carinata | Shrub |
| 337 | Calytrix creswellii | Shrub |
| 338 | Calytrix exstipulata | Shrub |
| 339 | Calytrix tetragona | Shrub |
| 340 | Capillipedium parviflorum | Tussock grass |
| 341 | Capillipedium sp. | Tussock grass |
| 342 | Capparis lasiantha | Vine |
| 343 | Capparis umbonata | Shrub |
| 344 | Carissa lanceolata | Shrub |
| 345 | Carpobrotus virescens | Shrub |
| 346 | Carrichtera annua | Forb |
| 347 | Cartonema spicatum | Forb |
| 348 | Cassinia complanata | Shrub |
| 349 | Cassinia laevis | Shrub |
| 350 | Cassytha capillaris | Vine |
| 351 | Cassytha filiformis | Vine |
| 352 | Cassytha glabella | Vine |
| 353 | Cassytha melantha | Vine |
| 354 | Cassytha peninsularis | Vine |
| 355 | Cassytha pubescens | Vine |
| 356 | Casuarina pauper | Tree |
| 357 | Casuarina stricta | Shrub |
| 358 | Cathormion umbellatum | Shrub |
| 359 | Cathormion umbellatum subsp. moniliforme | Shrub |
| 360 | Celtis philippensis | Tree |
| 361 | Cenchrus ciliaris | Tussock grass |
| 362 | Centaurea melitensis | Forb |
| 363 | Centipeda thespidioides | Forb |
| 364 | Centrolepis strigosa subsp. rupestris | Forb |
| 365 | Cephalipterum drummondii | Forb |
| 366 | Chamaecrista absus | Forb |
| 367 | Chamaecrista exigua var. exigua | Forb |
| 368 | Chamaecrista symonii | Forb |
| 369 | Chamaescilla corymbosa var. corymbosa | Forb |
| 370 | Chamaesyce drummondii | Forb |
| 371 | Chamaexeros fimbriata | Forb |
| 372 | Chamelaucium ciliatum s. lat. | Shrub |
| 373 | Chamelaucium pauciflorum subsp. pauciflorum | Shrub |
| 374 | Cheilanthes austrotenuifolia | Fern |
| 375 | Cheilanthes distans | Fern |
| 376 | Cheilanthes lasiophylla | Fern |
| 377 | Cheilanthes sieberi | Fern |
| 378 | Cheilanthes sieberi subsp. pseudovellea | Fern |
| 379 | Cheilanthes sieberi subsp. sieberi | Fern |
| 380 | Chenopodiaceae sp. | Chenopod |
| 381 | Chenopodium auricomum | Chenopod |
| 382 | Chenopodium cristatum | Chenopod |
| 383 | Chenopodium curvispicatum | Chenopod |
| 384 | Chenopodium desertorum | Chenopod |
| 385 | Chenopodium desertorum subsp. desertorum | Chenopod |
| 386 | Chenopodium desertorum subsp. microphyllum | Chenopod |
| 387 | Chenopodium desertorum subsp. anidiophyllum | Chenopod |
| 388 | Chenopodium gaudichaudianum | Chenopod |
| 389 | Chenopodium nitrariaceum | Chenopod |
| 390 | Chloris pectinata | Tussock grass |
| 391 | Chloris pumilio | Tussock grass |
| 392 | Chloris sp. | Tussock grass |
| 393 | Chloris truncata | Tussock grass |
| 394 | Chloris virgata | Tussock grass |
| 395 | Chrysitrix distigmatosa | Sedge |
| 396 | Chrysocephalum apiculatum | Forb |
| 397 | Chrysocephalum eremaeum | Shrub |
| 398 | Chrysocephalum pterochaetum | Shrub |
| 399 | Chrysocephalum puteale | Shrub |
| 400 | Chrysocephalum semicalvum | Forb |
| 401 | Chrysocephalum semipapposum | Forb |
| 402 | Chrysopogon elongatus | Tussock grass |
| 403 | Chrysopogon fallax | Tussock grass |
| 404 | Chrysopogon latifolius | Tussock grass |
| 405 | Chrysopogon pallidus | Tussock grass |
| 406 | Chrysopogon setifolius | Tussock grass |
| 407 | Chrysopogon sp. | Tussock grass |
| 408 | Chthonocephalus pseudevax | Forb |
| 409 | Citrullus lanatus | Vine |
| 410 | Cleistochloa subjuncea | Vine |
| 411 | Clematis microphylla | Vine |
| 412 | Cleome viscosa | Forb |
| 413 | Clerodendrum floribundum | Shrub |
| 414 | Clerodendrum tatei | Forb |
| 415 | Cochlospermum fraseri | Shrub |
| 416 | Cochlospermum gregorii | Tree |
| 417 | Codonocarpus pyramidalis | Tree |
| 418 | Coelospermum reticulatum | Shrub |
| 419 | Comesperma scoparium | Shrub |
| 420 | Commelina ensifolia | Forb |
| 421 | Conospermum stoechadis subsp. stoechadis | Shrub |
| 422 | Convolvulus angustissimus subsp. angustissimus | Forb |
| 423 | Convolvulus clementii | Forb |
| 424 | Convolvulus eyreanus | Forb |
| 425 | Convolvulus recurvatus subsp. nullarborensis | Forb |
| 426 | Convolvulus remotus | Forb |
| 427 | Convolvulus sp. | Forb |
| 428 | Conyza bonariensis | Forb |
| 429 | Corchorus olitorius | Forb |
| 430 | Corchorus sericeus | Forb |
| 431 | Corchorus sidoides | Forb |
| 432 | Corchorus sidoides subsp. vermicularis | Forb |
| 433 | Corchorus tridens | Forb |
| 434 | Correa aemula | Shrub |
| 435 | Corymbia aspera | Tree |
| 436 | Corymbia bella | Tree |
| 437 | Corymbia brachycarpa | Tree |
| 438 | Corymbia candida | Tree |
| 439 | Corymbia confertiflora | Tree |
| 440 | Corymbia dallachiana | Tree |
| 441 | Corymbia dichromophloia | Tree |
| 442 | Corymbia drysdalensis | Tree |
| 443 | Corymbia ferruginea | Tree |
| 444 | Corymbia ferruginea subsp. ferruginea | Tree |
| 445 | Corymbia flavescens | Tree |
| 446 | Corymbia grandifolia | Tree |
| 447 | Corymbia pocillum | Tree |
| 448 | Corymbia polycarpa | Shrub |
| 449 | Corymbia ptychocarpa subsp. ptychocarpa | Tree |
| 450 | Corymbia setosa | Tree |
| 451 | Corymbia setosa subsp. pedicellaris | Tree |
| 452 | Corymbia sp. | Tree |
| 453 | Corymbia terminalis | Tree |
| 454 | Corynotheca micrantha var. divaricata | Forb |
| 455 | Corynotheca sp. | Forb |
| 456 | Cotula cotuloides | Forb |
| 457 | Crassula colligata var. colligata | Forb |
| 458 | Crassula colorata var. acuminata | Forb |
| 459 | Crassula extrorsa | Forb |
| 460 | Cratystylis conocephala | Shrub |
| 461 | Cressa australis | Forb |
| 462 | Crotalaria cunninghamii subsp. sturtii | Forb |
| 463 | Crotalaria eremaea | Forb |
| 464 | Crotalaria eremaea subsp. eremaea | Forb |
| 465 | Crotalaria eremaea subsp. strehlowii | Forb |
| 466 | Crotalaria medicaginea var. neglecta | Forb |
| 467 | Crotalaria montana var. exserta | Forb |
| 468 | Crotalaria novae-hollandiae | Forb |
| 469 | Crotalaria novae-hollandiae subsp. novae-hollandiae | Forb |
| 470 | Crotalaria smithiana | Forb |
| 471 | Croton arnhemicus | Tree |
| 472 | Cryptandra apetala var. anomala | Shrub |
| 473 | Cryptandra minutifolia subsp. brevistyla | Shrub |
| 474 | Cryptandra polyclada subsp. aequabilis | Shrub |
| 475 | Cryptandra tomentosa | Shrub |
| 476 | Cryptandra wilsonii | Shrub |
| 477 | Cryptostegia grandiflora | Vine |
| 478 | Cucumis argenteus | Vine |
| 479 | Cucumis melo | Vine |
| 480 | Cullen australasicum | Forb |
| 481 | Cullen cinereum | Forb |
| 482 | Cullen discolor | Forb |
| 483 | Cullen graveolens | Forb |
| 484 | Cullen patens | Forb |
| 485 | Cupaniopsis anacardioides | Tree |
| 486 | Cuscuta planiflora | Vine |
| 487 | Cuscuta victoriana | Vine |
| 488 | Cyanthillium cinereum | Forb |
| 489 | Cycas armstrongii | Shrub |
| 490 | Cymbopogon obtectus | Tussock grass |
| 491 | Cymbopogon ambiguus | Tussock grass |
| 492 | Cymbopogon bombycinus | Tussock grass |
| 493 | Cynanchum carnosum | Vine |
| 494 | Cynanchum floribundum | Vine |
| 495 | Cynodon dactylon | Tussock grass |
| 496 | Cyperaceae sp. | Tussock grass |
| 497 | Cyperus alterniflorus f. Oodnadatta | Sedge |
| 498 | Cyperus betchei | Sedge |
| 499 | Cyperus bifax | Sedge |
| 500 | Cyperus conicus var. conicus | Sedge |
| 501 | Cyperus decompositus | Sedge |
| 502 | Cyperus fulvus | Sedge |
| 503 | Cyperus gilesii | Sedge |
| 504 | Cyperus holoschoenus | Sedge |
| 505 | Cyperus iria | Sedge |
| 506 | Cyperus nervulosus | Sedge |
| 507 | Cyperus pulchellus | Sedge |
| 508 | Cyperus sp. | Sedge |
| 509 | Cyperus victoriensis | Sedge |
| 510 | Dactyloctenium radulans | Tussock grass |
| 511 | Dactylis sp. | Tussock grass |
| 512 | Dampiera cinerea | Shrub |
| 513 | Dampiera dysantha | Shrub |
| 514 | Dampiera lavandulacea | Shrub |
| 515 | Dampiera sp. | Shrub |
| 516 | Dampiera stenostachya | Shrub |
| 517 | Danthonia sp. | Tussock grass |
| 518 | Daucus glochidiatus | Forb |
| 519 | Daviesia argillacea | Shrub |
| 520 | Daviesia leptophylla | Shrub |
| 521 | Daviesia sarissa subsp. redacta | Shrub |
| 522 | Daviesia ulicifolia incarnata | Forb |
| 523 | Denhamia cunninghamii | Shrub |
| 524 | Denhamia obscura | Shrub |
| 525 | Denhamia oleaster | Tree |
| 526 | Desmodium brachypodum | Vine |
| 527 | Desmodium campylocaulon | Forb |
| 528 | Desmodium muelleri | Vine |
| 529 | Desmodium pullenii | Vine |
| 530 | Desmodium sp. | Vine |
| 531 | Dianella revoluta | Forb |
| 532 | Dianella sp. | Forb |
| 533 | Dichanthium annulatum | Tussock grass |
| 534 | Dichanthium aristatum | Tussock grass |
| 535 | Dichanthium fecundum | Tussock grass |
| 536 | Dichanthium sericeum | Tussock grass |
| 537 | Dichanthium sericeum subsp. humilis | Tussock grass |
| 538 | Dichanthium sericeum subsp. polystachyum | Tussock grass |
| 539 | Dichanthium sericeum subsp. sericeum | Tussock grass |
| 540 | Dichelachne crinita | Tussock grass |
| 541 | Dichelachne micrantha | Tussock grass |
| 542 | Dichrostachys spicata | Shrub |
| 543 | Dicrastylis costelloi | Shrub |
| 544 | Dicrastylis lewellinii | Forb |
| 545 | Dicrastylis sp. | Forb |
| 546 | Digitaria ammophila | Tussock grass |
| 547 | Digitaria brownii | Tussock grass |
| 548 | Digitaria coenicola | Tussock grass |
| 549 | Digitaria divaricatissima | Tussock grass |
| 550 | Digitaria longiflora | Tussock grass |
| 551 | Digitaria papposa | Tussock grass |
| 552 | Digitaria sp. | Tussock grass |
| 553 | Dillwynia hispida | Forb |
| 554 | Dimeria ornithopoda | Tussock grass |
| 555 | Diospyros humilis | Tree |
| 556 | Diplachne fusca | Tussock grass |
| 557 | Diplatia grandibractea | Epiphyte |
| 558 | Dipteracanthus australasicus subsp. australasicus | Forb |
| 559 | Disphyma crassifolium subsp. clavellatum | Forb |
| 560 | Dissocarpus biflorus | Chenopod |
| 561 | Dissocarpus fontinalis | Chenopod |
| 562 | Dissocarpus paradoxa | Chenopod |
| 563 | Dissocarpus sp. | Chenopod |
| 564 | Dodonaea adenophora | Shrub |
| 565 | Dodonaea amblyophylla | Shrub |
| 566 | Dodonaea barklyensis | Shrub |
| 567 | Dodonaea baueri | Shrub |
| 568 | Dodonaea bursariifolia | Shrub |
| 569 | Dodonaea dodecandra | Shrub |
| 570 | Dodonaea hispidula var. phylloptera | Shrub |
| 571 | Dodonaea lobulata | Shrub |
| 572 | Dodonaea microzyga var. microzyga | Shrub |
| 573 | Dodonaea oxyptera | Shrub |
| 574 | Dodonaea physocarpa | Shrub |
| 575 | Dodonaea sp. | Shrub |
| 576 | Dodonaea stenozyga | Shrub |
| 577 | Dodonaea viscosa subsp. cuneata | Shrub |
| 578 | Dodonaea viscosa subsp. angustissima | Shrub |
| 579 | Dodonaea viscosa subsp. spatulata | Shrub |
| 580 | Dolichandrone heterophylla | Tree |
| 581 | Drosera andersoniana | Forb |
| 582 | Drosera auriculata | Forb |
| 583 | Drosera derbyensis | Forb |
| 584 | Drosera indica | Forb |
| 585 | Drosera lanata | Forb |
| 586 | Drosera macrantha subsp. planchonii | Forb |
| 587 | Drosera moorei | Forb |
| 588 | Drosera peltata | Forb |
| 589 | Drosera petiolaris | Forb |
| 590 | Drosera whittakeri | Forb |
| 591 | Drummondita hassellii | Shrub |
| 592 | Duma coccoloboides | Shrub |
| 593 | Duma florulenta | Shrub |
| 594 | Dysphania cristata | Shrub |
| 595 | Dysphania melanocarpa | Shrub |
| 596 | Ecdeiocolea monostachya | Sedge |
| 597 | Echinochloa colona | Sedge |
| 598 | Echium plantagineum | Forb |
| 599 | Ectrosia gulliveri | Tussock grass |
| 600 | Ectrosia scabrida | Tussock grass |
| 601 | Ehretia saligna | Shrub |
| 602 | Ehrharta longiflora | Shrub |
| 603 | Einadia nutans | Chenopod |
| 604 | Einadia nutans subsp. nutans | Chenopod |
| 605 | Einadia nutans subsp. eremaea | Chenopod |
| 606 | Eleocharis acuta | Sedge |
| 607 | Eleocharis caespitosissima | Sedge |
| 608 | Eleocharis pallens | Sedge |
| 609 | Emilia sonchifolia var. sonchifolia | Forb |
| 610 | Enchylaena lanata | Chenopod |
| 611 | Enchylaena sp. | Chenopod |
| 612 | Enchylaena tomentosa | Chenopod |
| 613 | Enchylaena tomentosa subsp. tomentosa | Chenopod |
| 614 | Enekbatus cryptandroides | Shrub |
| 615 | Enekbatus eremaeus | Shrub |
| 616 | Enneapogon avenaceus | Tussock grass |
| 617 | Enneapogon caerulescens | Tussock grass |
| 618 | Enneapogon clelandii | Tussock grass |
| 619 | Enneapogon cylindricus | Tussock grass |
| 620 | Enneapogon gracilis | Tussock grass |
| 621 | Enneapogon lindleyanus | Tussock grass |
| 622 | Enneapogon nigricans | Tussock grass |
| 623 | Enneapogon polyphyllus | Tussock grass |
| 624 | Enneapogon purpurascens | Tussock grass |
| 625 | Enneapogon robustissimus | Tussock grass |
| 626 | Enteropogon minutus | Tussock grass |
| 627 | Enteropogon acicularis | Tussock grass |
| 628 | Enteropogon ramosus | Tussock grass |
| 629 | Eragrostis basedowii | Tussock grass |
| 630 | Eragrostis cilianensis | Tussock grass |
| 631 | Eragrostis cilianensis minor | Tussock grass |
| 632 | Eragrostis concinna | Tussock grass |
| 633 | Eragrostis cumingii | Tussock grass |
| 634 | Eragrostis curvula | Tussock grass |
| 635 | Eragrostis dielsii | Tussock grass |
| 636 | Eragrostis elongata | Tussock grass |
| 637 | Eragrostis eriopoda | Tussock grass |
| 638 | Eragrostis falcata | Tussock grass |
| 639 | Eragrostis fallax | Tussock grass |
| 640 | Eragrostis lacunaria | Tussock grass |
| 641 | Eragrostis laniflora | Tussock grass |
| 642 | Eragrostis leptocarpa | Tussock grass |
| 643 | Eragrostis parviflora | Tussock grass |
| 644 | Eragrostis schultzii | Tussock grass |
| 645 | Eragrostis setacea | Tussock grass |
| 646 | Eragrostis setifolia | Tussock grass |
| 647 | Eragrostis sp. | Tussock grass |
| 648 | Eragrostis speciosa | Tussock grass |
| 649 | Eragrostis stagnalis | Tussock grass |
| 650 | Eragrostis tenellula | Tussock grass |
| 651 | Eragrostis xerophila | Tussock grass |
| 652 | Eremophea spinosa | Chenopod |
| 653 | Eremophila forrestii | Shrub |
| 654 | Eremophila forrestii subsp. forrestii | Shrub |
| 655 | Eremophila alternifolia | Shrub |
| 656 | Eremophila bignoniiflora | Shrub |
| 657 | Eremophila bowmanii subsp. bowmanii | Shrub |
| 658 | Eremophila caperata | Shrub |
| 659 | Eremophila cordatisepala | Shrub |
| 660 | Eremophila decipiens subsp. decipiens | Shrub |
| 661 | Eremophila dempsteri | Shrub |
| 662 | Eremophila deserti | Shrub |
| 663 | Eremophila drummondii | Shrub |
| 664 | Eremophila duttonii | Shrub |
| 665 | Eremophila freelingii | Shrub |
| 666 | Eremophila gilesii | Shrub |
| 667 | Eremophila gilesii subsp. gilesii | Shrub |
| 668 | Eremophila glabra | Shrub |
| 669 | Eremophila glabra subsp. glabra | Shrub |
| 670 | Eremophila goodwinii | Shrub |
| 671 | Eremophila goodwinii subsp. capitata | Shrub |
| 672 | Eremophila granitica | Shrub |
| 673 | Eremophila hygrophana | Shrub |
| 674 | Eremophila interstans subsp. interstans | Shrub |
| 675 | Eremophila ionantha | Shrub |
| 676 | Eremophila latrobei | Shrub |
| 677 | Eremophila latrobei subsp. glabra | Shrub |
| 678 | Eremophila longifolia | Shrub |
| 679 | Eremophila macdonnellii | Shrub |
| 680 | Eremophila maculata | Shrub |
| 681 | Eremophila maculata subsp. maculata | Shrub |
| 682 | Eremophila mitchellii | Shrub |
| 683 | Eremophila obovata | Shrub |
| 684 | Eremophila obovata subsp. obovata | Shrub |
| 685 | Eremophila oppositifolia | Shrub |
| 686 | Eremophila oppositifolia subsp. angustifolia | Shrub |
| 687 | Eremophila platythamnos subsp. platythamnos | Shrub |
| 688 | Eremophila saligna | Shrub |
| 689 | Eremophila scoparia | Shrub |
| 690 | Eremophila sp. | Shrub |
| 691 | Eremophila sturtii | Shrub |
| 692 | Eremophila willsii | Shrub |
| 693 | Eremophila willsii subsp. integrifolia | Shrub |
| 694 | Eriachne aristidea | Tussock grass |
| 695 | Eriachne armitii | Tussock grass |
| 696 | Eriachne avenacea | Tussock grass |
| 697 | Eriachne benthamii | Tussock grass |
| 698 | Eriachne ciliata | Tussock grass |
| 699 | Eriachne glauca | Tussock grass |
| 700 | Eriachne helmsii | Tussock grass |
| 701 | Eriachne melicacea | Tussock grass |
| 702 | Eriachne mucronata | Tussock grass |
| 703 | Eriachne nervosa | Tussock grass |
| 704 | Eriachne obtusa | Tussock grass |
| 705 | Eriachne ovata | Tussock grass |
| 706 | Eriachne pulchella subsp. pulchella | Tussock grass |
| 707 | Eriachne triodioides | Tussock grass |
| 708 | Eriochiton sclerolaenoides | Chenopod |
| 709 | Eriochloa procera | Tussock grass |
| 710 | Erodium aureum | Forb |
| 711 | Erodium brachycarpum | Forb |
| 712 | Erodium carolinianum | Forb |
| 713 | Erodium cicutarium | Forb |
| 714 | Erodium crinitum | Forb |
| 715 | Erodium cygnorum | Forb |
| 716 | Erymophyllum ramosum subsp. ramosum | Forb |
| 717 | Erythrina vespertilio | Forb |
| 718 | Erythrophleum chlorostachys | Tree |
| 719 | Eucalyptus baxteri | Tree |
| 720 | Eucalyptus brachycalyx | Tree |
| 721 | Eucalyptus brachycorys | Tree |
| 722 | Eucalyptus camaldulensis | Tree |
| 723 | Eucalyptus camaldulensis subsp. acuta | Tree |
| 724 | Eucalyptus camaldulensis subsp. arida | Tree |
| 725 | Eucalyptus camaldulensis subsp. camaldulensis | Tree |
| 726 | Eucalyptus camaldulensis subsp. obtusa | Tree |
| 727 | Eucalyptus celastroides subsp. celastroides | Tree Mallee |
| 728 | Eucalyptus ceratocorys | Shrub Mallee |
| 729 | Eucalyptus chlorophylla | Tree |
| 730 | Eucalyptus cladocalyx | Tree |
| 731 | Eucalyptus clelandii | Tree Mallee |
| 732 | Eucalyptus coolabah | Tree |
| 733 | Eucalyptus cosmophylla | Tree Mallee |
| 734 | Eucalyptus crebra | Tree |
| 735 | Eucalyptus cyanophylla -- Eucalyptus dumosa intergrade | Tree Mallee |
| 736 | Eucalyptus cylindrocarpa | Tree Mallee |
| 737 | Eucalyptus dumosa | Tree Mallee |
| 738 | Eucalyptus dumosa subsp. dumosa | Tree Mallee |
| 739 | Eucalyptus fasciculosa | Tree Mallee |
| 740 | Eucalyptus flindersii | Tree Mallee |
| 741 | Eucalyptus gamophylla | Tree Mallee |
| 742 | Eucalyptus gillii | Tree Mallee |
| 743 | Eucalyptus gongylocarpa | Tree |
| 744 | Eucalyptus goniocalyx subsp. goniocalyx | Tree Mallee |
| 745 | Eucalyptus gracilis | Tree Mallee |
| 746 | Eucalyptus horistes | Shrub Mallee |
| 747 | Eucalyptus incrassata | Shrub Mallee |
| 748 | Eucalyptus intertexta | Tree |
| 749 | Eucalyptus kingsmillii subsp. kingsmillii | Tree Mallee |
| 750 | Eucalyptus largiflorens | Tree Mallee |
| 751 | Eucalyptus leptophylla | Tree Mallee |
| 752 | Eucalyptus leptopoda subsp. leptopoda | Shrub Mallee |
| 753 | Eucalyptus leptopoda subsp. subluta | Shrub Mallee |
| 754 | Eucalyptus leucophloia | Tree Mallee |
| 755 | Eucalyptus leucophloia subsp. euroa | Tree Mallee |
| 756 | Eucalyptus longicornis | Tree |
| 757 | Eucalyptus longissima | Tree |
| 758 | Eucalyptus loxophleba subsp. lissophloia | Tree Mallee |
| 759 | Eucalyptus macrorhyncha | Tree |
| 760 | Eucalyptus melanophloia - E. whitei intergrade | Tree |
| 761 | Eucalyptus melanophloia subsp. melanophloia | Tree |
| 762 | Eucalyptus microneura | Tree |
| 763 | Eucalyptus microtheca | Tree |
| 764 | Eucalyptus miniata | Tree |
| 765 | Eucalyptus moderata | Tree Mallee |
| 766 | Eucalyptus obliqua | Tree |
| 767 | Eucalyptus odorata | Tree Mallee |
| 768 | Eucalyptus oldfieldii | Shrub Mallee |
| 769 | Eucalyptus oleosa | Tree Mallee |
| 770 | Eucalyptus oleosa subsp. oleosa | Tree Mallee |
| 771 | Eucalyptus oleosa subsp. ampliata | Tree Mallee |
| 772 | Eucalyptus pachyphylla | Shrub Mallee |
| 773 | Eucalyptus patellaris | Tree |
| 774 | Eucalyptus petraea | Tree Mallee |
| 775 | Eucalyptus phoenicea | Tree |
| 776 | Eucalyptus platycorys | Shrub Mallee |
| 777 | Eucalyptus polybractea | Tree Mallee |
| 778 | Eucalyptus populnea | Tree |
| 779 | Eucalyptus porosa | Tree Mallee |
| 780 | Eucalyptus pruinosa | Tree |
| 781 | Eucalyptus pruinosa subsp. pruinosa | Tree |
| 782 | Eucalyptus rigidula | Shrub Mallee |
| 783 | Eucalyptus salicola | Tree |
| 784 | Eucalyptus salmonophloia | Tree |
| 785 | Eucalyptus salubris | Tree |
| 786 | Eucalyptus similis | Tree |
| 787 | Eucalyptus socialis | Tree Mallee |
| 788 | Eucalyptus socialis subsp. socialis | Tree Mallee |
| 789 | Eucalyptus sp. | Tree Mallee |
| 790 | Eucalyptus tectifica | Tree |
| 791 | Eucalyptus tenera | Tree Mallee |
| 792 | Eucalyptus tetrodonta | Tree |
| 793 | Eucalyptus transcontinentalis | Tree |
| 794 | Eucalyptus urna | Tree |
| 795 | Eucalyptus yalatensis | Tree |
| 796 | Eucalyptus yilgarnensis | Tree Mallee |
| 797 | Euchiton sp. | Forb |
| 798 | Eulalia aurea | Tussock grass |
| 799 | Eulalia sp. | Tussock grass |
| 800 | Euphorbia australis | Forb |
| 801 | Euphorbia biconvexa | Forb |
| 802 | Euphorbia centralis | Forb |
| 803 | Euphorbia coghlanii | Forb |
| 804 | Euphorbia dallachyana | Forb |
| 805 | Euphorbia drummondii | Forb |
| 806 | Euphorbia ferdinandi var. ferdinandi | Forb |
| 807 | Euphorbia multifaria | Forb |
| 808 | Euphorbia multifarious | Forb |
| 809 | Euphorbia porcata | Forb |
| 810 | Euphorbia schultzii | Forb |
| 811 | Euphorbia sp. | Forb |
| 812 | Euphorbia tannensis subsp. eremophila | Forb |
| 813 | Euphorbia tannensis var. finlaysonii | Forb |
| 814 | Euphorbia wheeleri | Forb |
| 815 | Euphorbiaceae sp. | Forb |
| 816 | Euryomyrtus leptospermoides | Shrub |
| 817 | Euryomyrtus maidenii | Shrub |
| 818 | Eutaxia microphylla | Shrub |
| 819 | Eutaxia neurocalyx subsp. nacta | Shrub |
| 820 | Evolvulus alsinoides | Forb |
| 821 | Excoecaria parvifolia | Tree |
| 822 | Exocarpos aphylla | Tree |
| 823 | Exocarpos cupressiformis | Shrub |
| 824 | Exocarpos latifolia | Shrub |
| 825 | Exocarpos sparteus | Shrub |
| 826 | Ficus opposita | Shrub |
| 827 | Fimbristylis ammobia | Sedge |
| 828 | Fimbristylis bisumbellata | Sedge |
| 829 | Fimbristylis caespitosa | Sedge |
| 830 | Fimbristylis cinnamometorum | Sedge |
| 831 | Fimbristylis depauperata | Sedge |
| 832 | Fimbristylis dichotoma | Sedge |
| 833 | Fimbristylis littoralis | Sedge |
| 834 | Fimbristylis macrantha | Sedge |
| 835 | Fimbristylis microcarya | Sedge |
| 836 | Fimbristylis odontocarpa | Sedge |
| 837 | Fimbristylis oxystachya | Sedge |
| 838 | Fimbristylis pachyptera | Sedge |
| 839 | Fimbristylis pterigosperma | Sedge |
| 840 | Fimbristylis simplex | Sedge |
| 841 | Fimbristylis sp. | Sedge |
| 842 | Fimbristylis squarrulosa | Sedge |
| 843 | Fimbristylis subaristata | Sedge |
| 844 | Fimbristylis tetragona | Sedge |
| 845 | Fimbristylis xyridis | Sedge |
| 846 | Flemingia parviflora | Forb |
| 847 | Flemingia pauciflora | Forb |
| 848 | Flindersia maculosa | Tree |
| 849 | Flueggea virosa | Shrub |
| 850 | Flueggea virosa subsp. melanthesoides | Shrub |
| 851 | Frankenia cinerea | Shrub |
| 852 | Frankenia desertorum | Shrub |
| 853 | Frankenia eremophila | Shrub |
| 854 | Frankenia serpyllifolia | Forb |
| 855 | Frankenia sessilis | Forb |
| 856 | Frankenia sp. | Forb |
| 857 | Fuirena ciliaris | Sedge |
| 858 | Fuirena incrassata | Sedge |
| 859 | Galactia tenuifolia | Vine |
| 860 | Galactia sp. | Vine |
| 861 | Galium murale | Forb |
| 862 | Gardenia megasperma | Shrub |
| 863 | Gardenia vilhelmii | Shrub |
| 864 | Geijera linearifolia | Shrub |
| 865 | Glinus lotoides | Forb |
| 866 | Glischrocaryon aureum | Shrub |
| 867 | Glischrocaryon behrii | Forb |
| 868 | Glycine canescens | Vine |
| 869 | Glycine falcata | Vine |
| 870 | Glycine rubiginosa | Forb |
| 871 | Glycine sp. | Forb |
| 872 | Gnephosis arachnoidea | Forb |
| 873 | Gnephosis eriocarpa | Forb |
| 874 | Gnephosis tenuissima | Forb |
| 875 | Gomphrena breviflora | Forb |
| 876 | Gomphrena canescens | Forb |
| 877 | Gomphrena celosioides | Forb |
| 878 | Gomphrena lanata | Forb |
| 879 | Gonocarpus chinensis | Forb |
| 880 | Gonocarpus elatus | Forb |
| 881 | Gonocarpus leptothecus | Forb |
| 882 | Gonocarpus mezianus | Forb |
| 883 | Gonocarpus tetragynus | Forb |
| 884 | Goodenia albiflora | Forb |
| 885 | Goodenia armitiana | Forb |
| 886 | Goodenia blackiana | Forb |
| 887 | Goodenia cycloptera | Forb |
| 888 | Goodenia fascicularis | Forb |
| 889 | Goodenia geniculata | Forb |
| 890 | Goodenia glabra | Forb |
| 891 | Goodenia havilandii | Forb |
| 892 | Goodenia hispida | Forb |
| 893 | Goodenia janamba | Forb |
| 894 | Goodenia lunata | Forb |
| 895 | Goodenia pinnatifida | Forb |
| 896 | Goodenia sp. | Forb |
| 897 | Goodenia strangfordii | Forb |
| 898 | Goodenia triodiophila | Forb |
| 899 | Goodenia vernicosa | Forb |
| 900 | Goodenia vilmoriniae | Forb |
| 901 | Graminea sp. | Tussock grass |
| 902 | Grevillea acuaria | Shrub |
| 903 | Grevillea albiflora | Shrub |
| 904 | Grevillea aspera | Shrub |
| 905 | Grevillea cagiana | Shrub |
| 906 | Grevillea ceratocarpa | Shrub |
| 907 | Grevillea decurrens/ heliosperma | Shrub |
| 908 | Grevillea didymobotrya subsp. didymobotrya | Shrub |
| 909 | Grevillea dryandri subsp. dryandri | Shrub |
| 910 | Grevillea eremophila | Shrub |
| 911 | Grevillea eriostachya | Shrub |
| 912 | Grevillea excelsior | Shrub |
| 913 | Grevillea glauca | Tree |
| 914 | Grevillea haplantha subsp. haplantha | Shrub |
| 915 | Grevillea heliosperma | Tree |
| 916 | Grevillea hookeriana subsp. apiciloba | Shrub |
| 917 | Grevillea huegelii | Shrub |
| 918 | Grevillea juncifolia subsp. juncifolia | Shrub |
| 919 | Grevillea juncifolia subsp. temulenta | Shrub |
| 920 | Grevillea lavandulacea subsp. lavandulacea | Shrub |
| 921 | Grevillea mimosoides | Shrub |
| 922 | Grevillea nematophylla subsp. supraplana | Shrub |
| 923 | Grevillea oncogyne | Shrub |
| 924 | Grevillea paradoxa | Shrub |
| 925 | Grevillea parallela | Shrub |
| 926 | Grevillea pluricaulis | Shrub |
| 927 | Grevillea pteridifolia | Shrub |
| 928 | Grevillea pterosperma | Shrub |
| 929 | Grevillea refracta | Shrub |
| 930 | Grevillea sarissa subsp. anfractifolia | Shrub |
| 931 | Grevillea sp. | Shrub |
| 932 | Grevillea stenobotrya | Shrub |
| 933 | Grevillea striata | Tree |
| 934 | Grevillea teretifolia | Shrub |
| 935 | Grevillea wickhamii | Shrub |
| 936 | Grewia retusifolia | Shrub |
| 937 | Gunniopsis calcarea | Shrub |
| 938 | Gunniopsis kochii | Forb |
| 939 | Gunniopsis quadrifida | Shrub |
| 940 | Gymnanthera oblonga | Shrub |
| 941 | Gyrocarpus americanus | Shrub |
| 942 | Haemodorum brevicaule | Forb |
| 943 | Haemodorum coccineum | Forb |
| 944 | Hakea arborescens | Shrub |
| 945 | Hakea carinata | Shrub |
| 946 | Hakea chordophylla | Shrub |
| 947 | Hakea divaricata | Tree |
| 948 | Hakea erecta | Shrub |
| 949 | Hakea eyreana | Shrub |
| 950 | Hakea francisiana | Shrub |
| 951 | Hakea leucoptera | Shrub |
| 952 | Hakea lorea | Shrub |
| 953 | Hakea macrocarpa | Shrub |
| 954 | Hakea minima | Shrub |
| 955 | Hakea rostrata | Shrub |
| 956 | Hakea rugosa | Shrub |
| 957 | Hakea scoparia subsp. scoparia | Shrub |
| 958 | Hakea sp. | Shrub |
| 959 | Halgania andromedifolia | Shrub |
| 960 | Halgania cyanea | Forb |
| 961 | Halgania erecta | Shrub |
| 962 | Halgania integerrima | Shrub |
| 963 | Haloragis aspera | Forb |
| 964 | Haloragis glauca forma sclopetifera | Forb |
| 965 | Haloragis sp. | Forb |
| 966 | Harmsiodoxa puberula | Forb |
| 967 | Helichrysum scorpioides | Forb |
| 968 | Helicteres cana | Forb |
| 969 | Helicteres tenuipila | Forb |
| 970 | Heliotropium asperrimum | Forb |
| 971 | Heliotropium conocarpum | Forb |
| 972 | Heliotropium consimile | Forb |
| 973 | Heliotropium cunninghamii | Forb |
| 974 | Heliotropium leptaleum | Forb |
| 975 | Heliotropium moorei | Forb |
| 976 | Heliotropium plumosum | Forb |
| 977 | Heliotropium sp. | Forb |
| 978 | Heliotropium tenuifolium | Forb |
| 979 | Helipterum sp. | Forb |
| 980 | Hemichroa diandra | Forb |
| 981 | Herissantia crispa | Forb |
| 982 | Heteropogon contortus | Tussock grass |
| 983 | Heteropogon triticeus | Tussock grass |
| 984 | Hibbertia brevipedunculata | Forb |
| 985 | Hibbertia crinita | Shrub |
| 986 | Hibbertia dilatata | Shrub |
| 987 | Hibbertia eatoniae | Shrub |
| 988 | Hibbertia exutiacies | Shrub |
| 989 | Hibbertia juncea | Shrub |
| 990 | Hibbertia lepidota | Shrub |
| 991 | Hibbertia riparia | Shrub |
| 992 | Hibbertia rostellata | Shrub |
| 993 | Hibbertia tasmanica | Shrub |
| 994 | Hibbertia virgata | Forb |
| 995 | Hibbertia lepidota | Forb |
| 996 | Hibiscus brachysiphonius | Forb |
| 997 | Hibiscus burtonii | Forb |
| 998 | Hibiscus krichauffianus | Forb |
| 999 | Hibiscus meraukensis | Forb |
| 1000 | Hibiscus sturtii | Forb |
| 1001 | Hibiscus verdcourtii | Forb |
| 1002 | Homalocalyx grandiflorus | Shrub |
| 1003 | Homalocalyx pulcherrimus | Shrub |
| 1004 | Homalocalyx thryptomenoides | Shrub |
| 1005 | Hovea parvicalyx | Shrub |
| 1006 | Hyalochlamys globifera | Forb |
| 1007 | Hyalosperma glutinosum subsp. glutinosum | Forb |
| 1008 | Hybanthus aurantiacus | Forb |
| 1009 | Hybanthus floribundus | Shrub |
| 1010 | Hybanthus monopetalus | Forb |
| 1011 | Hydrocotyle diantha | Forb |
| 1012 | Hygrophila angustifolia | Forb |
| 1013 | Hypochaeris glabra | Forb |
| 1014 | Hyptis suaveolens | Forb |
| 1015 | Indigastrum parviflorum | Forb |
| 1016 | Indigofera colutea | Forb |
| 1017 | Indigofera cornuligera | Shrub |
| 1018 | Indigofera cornuligera subsp. flindersensis | Shrub |
| 1019 | Indigofera ewartiana | Forb |
| 1020 | Indigofera georgei | Shrub |
| 1021 | Indigofera hirsuta | Forb |
| 1022 | Indigofera linifolia | Forb |
| 1023 | Indigofera linnaei | Forb |
| 1024 | Indigofera longibractea | Forb |
| 1025 | Indigofera polygaloides | Forb |
| 1026 | Indigofera pratensis | Forb |
| 1027 | Indigofera psammophila | Forb |
| 1028 | Ipomoea coptica | Forb |
| 1029 | Ipomoea lonchophylla | Forb |
| 1030 | Ipomoea muelleri | Vine |
| 1031 | Ipomoea plebeia | Vine |
| 1032 | Ipomoea polymorpha | Forb |
| 1033 | Ischaemum australe var. arundinaceum | Tussock grass |
| 1034 | Iseilema convexum | Tussock grass |
| 1035 | Iseilema fragile | Tussock grass |
| 1036 | Iseilema macratherum | Tussock grass |
| 1037 | Iseilema membranaceum | Tussock grass |
| 1038 | Iseilema vaginiflorum | Tussock grass |
| 1039 | Isopogon ceratophyllus | Shrub |
| 1040 | Ixiochlamys cuneifolia | Forb |
| 1041 | Ixodia achillaeoides subsp. alata | Shrub |
| 1042 | Jacksonia aculeata | Shrub |
| 1043 | Jacksonia nematoclada | Shrub |
| 1044 | Jacksonia odontoclada | Shrub |
| 1045 | Juncus aridicola | Rush |
| 1046 | Juncus subsecundus | Rush |
| 1047 | Kennedia prorepens | Vine |
| 1048 | Kennedia prostrata | Vine |
| 1049 | Keraudrenia velutina subsp. elliptica | Shrub |
| 1050 | Keraudrenia velutina subsp. velutina | Shrub |
| 1051 | Lawrencia repens | Chenopod |
| 1052 | Lawrencia squamata | Chenopod |
| 1053 | Lechenaultia divaricata | Shrub |
| 1054 | Leiocarpa leptolepis | Forb |
| 1055 | Leiocarpa semicalva subsp. semicalva | Forb |
| 1056 | Leiocarpa sp. | Forb |
| 1057 | Leiocarpa websteri | Forb |
| 1058 | Lepidium muelleri-ferdinandi | Forb |
| 1059 | Lepidium oxytrichum | Forb |
| 1060 | Lepidium papillosum | Forb |
| 1061 | Lepidium phlebopetalum | Shrub |
| 1062 | Lepidobolus preissianus subsp. volubilis | Sedge |
| 1063 | Lepidosperma aff. lyonsii | Sedge |
| 1064 | Lepidosperma carphoides | Sedge |
| 1065 | Lepidosperma rigidulum | Sedge |
| 1066 | Lepidosperma sanguinolentum | Sedge |
| 1067 | Lepidosperma semiteres | Sedge |
| 1068 | Lepidosperma sp. | Sedge |
| 1069 | Lepidosperma viscidum | Sedge |
| 1070 | Leptochloa digitata | Tussock grass |
| 1071 | Leptochloa neesii | Tussock grass |
| 1072 | Leptomeria preissiana | Shrub |
| 1073 | Leptosema aculeatum | Shrub |
| 1074 | Leptosema chambersii | Shrub |
| 1075 | Leptospermum continentale | Shrub |
| 1076 | Leptospermum fastigiatum | Shrub |
| 1077 | Leptospermum myrsinoides | Shrub |
| 1078 | Leptospermum spinescens | Shrub |
| 1079 | Leucochrysum stipitatum | Forb |
| 1080 | Leucopogon concurvus | Shrub |
| 1081 | Leucopogon hamulosus | Shrub |
| 1082 | Leucopogon lanceolatus | Shrub |
| 1083 | Leucopogon sp. | Shrub |
| 1084 | Levenhookia leptantha | Forb |
| 1085 | Limonium lobatum | Forb |
| 1086 | Lipocarpha microcephala | Sedge |
| 1087 | Livistona humilis | Tree |
| 1088 | Lolium rigidum | Tussock grass |
| 1089 | Lomandra collina | Forb |
| 1090 | Lomandra densiflora | Forb |
| 1091 | Lomandra effusa | Forb |
| 1092 | Lomandra fibrata | Forb |
| 1093 | Lomandra micrantha subsp. tuberculata | Forb |
| 1094 | Lomandra multiflora subsp. dura | Forb |
| 1095 | Lomandra multiflora subsp. multiflora | Forb |
| 1096 | Lotus cruentus | Forb |
| 1097 | Lumnitzera racemosa | Shrub |
| 1098 | Lycium australe | Shrub |
| 1099 | Lycium ferocissimum | Shrub |
| 1100 | Lysiana casuarinae | Epiphyte |
| 1101 | Lysiana exocarpi | Epiphyte |
| 1102 | Lysiana exocarpi subsp. exocarpi | Epiphyte |
| 1103 | Lysiana linearifolia | Epiphyte |
| 1104 | Lysiana spathulata | Epiphyte |
| 1105 | Lysiana subfalcata | Epiphyte |
| 1106 | Lysimachia arvensis | Forb |
| 1107 | Lysiphyllum carronii | Tree |
| 1108 | Lysiphyllum cunninghamii | Tree |
| 1109 | Lysiphyllum gilvum | Tree |
| 1110 | Macropteranthes kekwickii | Tree |
| 1111 | Maireana pentatropis | Chenopod |
| 1112 | Maireana aff. planifolia (narrow morphotype - P. Wilson) | Chenopod |
| 1113 | Maireana aphylla | Chenopod |
| 1114 | Maireana astrotricha | Chenopod |
| 1115 | Maireana brevifolia | Chenopod |
| 1116 | Maireana ciliata | Chenopod |
| 1117 | Maireana coronata | Chenopod |
| 1118 | Maireana dichoptera | Chenopod |
| 1119 | Maireana eriantha | Chenopod |
| 1120 | Maireana erioclada | Chenopod |
| 1121 | Maireana georgei | Chenopod |
| 1122 | Maireana georgei x Enchylaena tomentosa | Chenopod |
| 1123 | Maireana integra | Chenopod |
| 1124 | Maireana lanosa | Chenopod |
| 1125 | Maireana oppositifolia | Chenopod |
| 1126 | Maireana pentatropis | Chenopod |
| 1127 | Maireana pyramidata | Chenopod |
| 1128 | Maireana radiata | Chenopod |
| 1129 | Maireana rohrlachii | Chenopod |
| 1130 | Maireana scleroptera | Chenopod |
| 1131 | Maireana sedifolia | Chenopod |
| 1132 | Maireana sp. | Chenopod |
| 1133 | Maireana spongiocarpa | Chenopod |
| 1134 | Maireana thesioides | Chenopod |
| 1135 | Maireana tomentosa subsp. tomentosa | Chenopod |
| 1136 | Maireana trichoptera | Chenopod |
| 1137 | Maireana triptera | Chenopod |
| 1138 | Maireana turbinata | Chenopod |
| 1139 | Maireana villosa | Chenopod |
| 1140 | Malacocera tricornis | Chenopod |
| 1141 | Malva preissiana | Forb |
| 1142 | Malva weinmanniana | Forb |
| 1143 | Malvastrum americanum | Forb |
| 1144 | Marianthus bicolor | Shrub |
| 1145 | Marrubium vulgare | Forb |
| 1146 | Marsdenia australis | Vine |
| 1147 | Marsdenia viridiflora | Vine |
| 1148 | Marsdenia viridiflora subsp. tropica | Vine |
| 1149 | Marsilea drummondii | Fern |
| 1150 | Marsilea exarata | Fern |
| 1151 | Marsilea hirsuta | Fern |
| 1152 | Marsilea sp. | Fern |
| 1153 | Maytenus cunninghamii | Shrub |
| 1154 | Medicago minima | Forb |
| 1155 | Medicago polymorpha | Forb |
| 1156 | Melaleuca acuminata subsp. acuminata | Shrub |
| 1157 | Melaleuca argentea | Shrub |
| 1158 | Melaleuca bracteata | Shrub |
| 1159 | Melaleuca calyptroides | Shrub |
| 1160 | Melaleuca citrolens | Tree |
| 1161 | Melaleuca cordata | Shrub |
| 1162 | Melaleuca glomerata | Shrub |
| 1163 | Melaleuca hamata | Shrub |
| 1164 | Melaleuca lanceolata | Shrub |
| 1165 | Melaleuca lateriflora | Shrub |
| 1166 | Melaleuca laxiflora | Shrub |
| 1167 | Melaleuca nervosa | Shrub |
| 1168 | Melaleuca pauperiflora subsp. fastigiata | Shrub |
| 1169 | Melaleuca protrusa | Shrub |
| 1170 | Melaleuca quadrifaria | Shrub |
| 1171 | Melaleuca scalena | Shrub |
| 1172 | Melaleuca sp. | Shrub |
| 1173 | Melaleuca stenostachya | Shrub |
| 1174 | Melaleuca symphyocarpa | Shrub |
| 1175 | Melaleuca trichostachya | Shrub |
| 1176 | Melaleuca uncinata | Shrub |
| 1177 | Melaleuca viridiflora | Tree |
| 1178 | Melhania oblongifolia | Forb |
| 1179 | Melinis repens | Tussock grass |
| 1180 | Melochia corchorifolia | Forb |
| 1181 | Mesembryanthemum nodiflorum | Forb |
| 1182 | Mesomelaena preissii | Sedge |
| 1183 | Micrantheum demissum | Shrub |
| 1184 | Microcybe multiflora subsp. multiflora | Shrub |
| 1185 | Microlaena stipoides var. stipoides | Shrub |
| 1186 | Micromyrtus monotaxis | Shrub |
| 1187 | Micromyrtus erichsenii | Shrub |
| 1188 | Micromyrtus flaviflora | Shrub |
| 1189 | Microstachys chamaelea | Forb |
| 1190 | Microtis sp. | Forb |
| 1191 | Millotia perpusilla | Forb |
| 1192 | Minuria cunninghamii | Shrub |
| 1193 | Minuria integerrima | Forb |
| 1194 | Minuria leptophylla | Forb |
| 1195 | Minuria rigida | Forb |
| 1196 | Mirbelia microphylla | Shrub |
| 1197 | Mitrasacme connata | Shrub |
| 1198 | Mnesithea formosa | Tussock grass |
| 1199 | Mnesithea rottboellioides | Tussock grass |
| 1200 | Monachather paradoxa | Tussock grass |
| 1201 | Monocot sp. | Shrub |
| 1202 | Muehlenbeckia florulenta | Shrub |
| 1203 | Murdannia graminea | Forb |
| 1204 | Myoporum acuminatum | Shrub |
| 1205 | Myoporum insulare | Shrub |
| 1206 | Myoporum montanum | Shrub |
| 1207 | Myoporum platycarpum | Shrub |
| 1208 | Myoporum platycarpum subsp. platycarpum | Tree |
| 1209 | Myriocephalus pygmaeus | Forb |
| 1210 | Neobassia proceriflora | Chenopod |
| 1211 | Neptunia dimorphantha | Forb |
| 1212 | Neptunia gracilis forma glandulosa | Forb |
| 1213 | Neptunia monosperma | Forb |
| 1214 | Neurachne alopecuroidea | Tussock grass |
| 1215 | Neurachne munroi | Tussock grass |
| 1216 | Newcastelia cephalantha | Tussock grass |
| 1217 | Newcastelia spodiotricha | Tussock grass |
| 1218 | Nicotiana goodspeedii | Shrub |
| 1219 | Nicotiana simulans | Forb |
| 1220 | Nicotiana velutina | Forb |
| 1221 | Nitraria billardierei | Shrub |
| 1222 | Notoleptopus decaisnei | Shrub |
| 1223 | Nymphaea carpentariae | Forb |
| 1224 | Ocimum tenuiflorum | Forb |
| 1225 | Oldenlandia mitrasacmoides subsp. mitrasacmoides | Forb |
| 1226 | Oldenlandia mitrasacmoides subsp. nigricans | Forb |
| 1227 | Oldenlandia pterospora | Forb |
| 1228 | Olearia dampieri subsp. Eremicola (Diels & Pritzel s.n. PERTH 00449628) | Forb |
| 1229 | Olearia decurrens | Forb |
| 1230 | Olearia exiguifolia | Shrub |
| 1231 | Olearia floribunda | Shrub |
| 1232 | Olearia grandiflora | Forb |
| 1233 | Olearia muelleri | Shrub |
| 1234 | Olearia passerinoides subsp. passerinoides | Shrub |
| 1235 | Olearia pimelioides | Shrub |
| 1236 | Olearia ramulosa | Shrub |
| 1237 | Olearia subspicata | Shrub |
| 1238 | Olearia teretifolia | Shrub |
| 1239 | Opercularia turpis | Forb |
| 1240 | Ophioglossum lusitanicum | Fern |
| 1241 | Ophiuros exaltatus | Tussock grass |
| 1242 | Oryza australiensis | Tussock grass |
| 1243 | Osteocarpum dipterocarpum | Chenopod |
| 1244 | Owenia acidula | Shrub |
| 1245 | Owenia vernicosa | Tree |
| 1246 | Oxalis perennans | Forb |
| 1247 | Oxychloris scariosa | Tussock grass |
| 1248 | Ozothamnus retusus | Shrub |
| 1249 | Pandanus spiralis | Shrub |
| 1250 | Panicum decompositum | Tussock grass |
| 1251 | Panicum effusum | Tussock grass |
| 1252 | Panicum laevinode | Tussock grass |
| 1253 | Panicum larcomianum | Tussock grass |
| 1254 | Panicum mindanaense | Tussock grass |
| 1255 | Panicum queenslandicum | Tussock grass |
| 1256 | Panicum seminudum var. cairnsianum | Tussock grass |
| 1257 | Panicum simile | Tussock grass |
| 1258 | Panicum sp. | Tussock grass |
| 1259 | Panicum trichoides | Tussock grass |
| 1260 | Paractaenum novae-hollandiae subsp. reversum | Tussock grass |
| 1261 | Paraneurachne muelleri | Tussock grass |
| 1262 | Parentucellia latifolia | Forb |
| 1263 | Parkinsonia aculeata | Shrub |
| 1264 | Paspalidium clementii | Tussock grass |
| 1265 | Paspalidium constrictum | Tussock grass |
| 1266 | Paspalidium distans | Tussock grass |
| 1267 | Paspalidium gracile var. Gracile | Tussock grass |
| 1268 | Paspalidium jubiflorum | Tussock grass |
| 1269 | Paspalidium rarum | Tussock grass |
| 1270 | Paspalidium reflexum | Tussock grass |
| 1271 | Paspalidium retiglume | Tussock grass |
| 1272 | Passiflora foetida | Vine |
| 1273 | Patersonia rudis subsp. velutina | Vine |
| 1274 | Pentameris airoides | Tussock grass |
| 1275 | Pentameris airoides subsp. airoides | Tussock grass |
| 1276 | Pentameris pallida | Tussock grass |
| 1277 | Pentaschistis pallida | Tussock grass |
| 1278 | Peplidium aithocheilum | Tussock grass |
| 1279 | Perotis rara | Tussock grass |
| 1280 | Persoonia coriacea | Shrub |
| 1281 | Persoonia falcata | Shrub |
| 1282 | Petalostigma banksii | Shrub |
| 1283 | Petalostigma pubescens | Tree |
| 1284 | Petalostigma quadriloculare | Shrub |
| 1285 | Petalostylis cassioides | Shrub |
| 1286 | Petrophile stricta | Shrub |
| 1287 | Phebalium laevigatum | Shrub |
| 1288 | Phebalium lepidotum | Shrub |
| 1289 | Pheidochloa gracilis | Tussock grass |
| 1290 | Phellinus sp. | Fungus |
| 1291 | Philotheca angustifolia subsp. angustifolia | Shrub |
| 1292 | Phyllanthus carpentariae | Forb |
| 1293 | Phyllanthus collinus | Forb |
| 1294 | Phyllanthus lacunarius | Forb |
| 1295 | Phyllanthus maderaspatensis | Forb |
| 1296 | Phyllanthus virgatus | Forb |
| 1297 | Pimelea aeruginosa | Shrub |
| 1298 | Pimelea angustifolia | Shrub |
| 1299 | Pimelea humilis | Shrub |
| 1300 | Pimelea linifolia | Shrub |
| 1301 | Pimelea microcephala subsp. microcephala | Shrub |
| 1302 | Pimelea simplex subsp. continua | Forb |
| 1303 | Pimelea stricta | Shrub |
| 1304 | Pimelea trichostachya | Shrub |
| 1305 | Pittosporum angustifolium | Shrub |
| 1306 | Pityrodia lepidota | Shrub |
| 1307 | Planchonia careya | Tree |
| 1308 | Plantago bellardii | Forb |
| 1309 | Plantago drummondii | Forb |
| 1310 | Plantago gaudichaudii | Forb |
| 1311 | Plantago sp. | Forb |
| 1312 | Platylobium obtusangulum | Forb |
| 1313 | Platysace trachymenioides | Shrub |
| 1314 | Pluchea dunlopii | Shrub |
| 1315 | Pluchea rubelliflora | Shrub |
| 1316 | Pluchea sp. | Forb |
| 1317 | Poa tenera | Tussock grass |
| 1318 | Poaceae sp. | Hummock grass |
| 1319 | Podolepis capillaris | Forb |
| 1320 | Podolepis lessonii | Forb |
| 1321 | Podolepis rugata | Forb |
| 1322 | Podolepis sp. | Forb |
| 1323 | Pogonolepis muelleriana | Forb |
| 1324 | Polycalymma stuartii | Forb |
| 1325 | Polycarpaea corymbosa | Forb |
| 1326 | Polygala bifoliata | Forb |
| 1327 | Polygala dependens | Forb |
| 1328 | Polygala pterocarpa | Forb |
| 1329 | Polymeria longifolia | Forb |
| 1330 | Polymeria sp. | Forb |
| 1331 | Pomaderris forrestiana | Forb |
| 1332 | Portulaca bicolor | Forb |
| 1333 | Portulaca digyna | Forb |
| 1334 | Portulaca filifolia | Forb |
| 1335 | Portulaca intraterranea | Forb |
| 1336 | Portulaca oleracea | Forb |
| 1337 | Portulaca oligosperma | Forb |
| 1338 | Portulaca pilosa | Forb |
| 1339 | Portulaca sp. | Forb |
| 1340 | Pouteria sericea | Shrub |
| 1341 | Prostanthera behriana | Shrub |
| 1342 | Prostanthera grylloana | Shrub |
| 1343 | Prostanthera semiteres subsp. semiteres | Shrub |
| 1344 | Prostanthera serpyllifolia subsp. microphylla | Forb |
| 1345 | Prostanthera striatiflora | Shrub |
| 1346 | Prostanthera wilkieana | Shrub |
| 1347 | Psammomoya choretroides | Shrub |
| 1348 | Pseudopogonatherum contortum | Tussock grass |
| 1349 | Pseudopogonatherum sp. | Tussock grass |
| 1350 | Pseudoraphis spinescens | Tussock grass |
| 1351 | Psydrax attenuata | Tree |
| 1352 | Psydrax latifolia | Shrub |
| 1353 | Psydrax oleifolia | Shrub |
| 1354 | Pteridium esculentum | Fern |
| 1355 | Pterocaulon serrulatum | Forb |
| 1356 | Pterocaulon sphacelatum | Forb |
| 1357 | Ptilotus clementii | Shrub |
| 1358 | Ptilotus erubescens | Shrub |
| 1359 | Ptilotus fusiformis | Forb |
| 1360 | Ptilotus holosericeus | Forb |
| 1361 | Ptilotus latifolius | Forb |
| 1362 | Ptilotus nobilis | Forb |
| 1363 | Ptilotus nobilis subsp. nobilis | Forb |
| 1364 | Ptilotus obovatus | Forb |
| 1365 | Ptilotus polystachyus | Forb |
| 1366 | Ptilotus sessilifolius | Forb |
| 1367 | Ptilotus spicatus | Forb |
| 1368 | Ptilotus whitei | Forb |
| 1369 | Pultenaea acerosa | Shrub |
| 1370 | Pultenaea daphnoides | Shrub |
| 1371 | Pultenaea involucrata | Shrub |
| 1372 | Pultenaea kraehenbuehlii | Shrub |
| 1373 | Pultenaea largiflorens | Forb |
| 1374 | Pultenaea prostrata | Forb |
| 1375 | Pultenaea sp. | Shrub |
| 1376 | Pycnosorus pleiocephalus | Shrub |
| 1377 | Pyrorchis nigricans | Forb |
| 1378 | Rapistrum rugosum | Forb |
| 1379 | Rhagodia crassifolia | Chenopod |
| 1380 | Rhagodia drummondii | Chenopod |
| 1381 | Rhagodia eremaea | Chenopod |
| 1382 | Rhagodia parabolica | Chenopod |
| 1383 | Rhagodia paradoxa | Chenopod |
| 1384 | Rhagodia preissii subsp. preissii | Chenopod |
| 1385 | Rhagodia sp. | Chenopod |
| 1386 | Rhagodia spinescens | Chenopod |
| 1387 | Rhagodia ulicina | Chenopod |
| 1388 | Rhodanthe corymbiflora | Forb |
| 1389 | Rhodanthe floribunda | Forb |
| 1390 | Rhodanthe haigii | Forb |
| 1391 | Rhodanthe microglossa | Forb |
| 1392 | Rhodanthe moschata | Forb |
| 1393 | Rhodanthe pygmaea | Forb |
| 1394 | Rhodanthe rubella | Forb |
| 1395 | Rhodanthe tietkensii | Forb |
| 1396 | Rhodanthe uniflora | Forb |
| 1397 | Rhyncharrhena linearis | Vine |
| 1398 | Rhynchosia minima | Vine |
| 1399 | Rhynchospora exserta | Sedge |
| 1400 | Rhynchospora longisetis | Sedge |
| 1401 | Rhynchospora sp. (Croydon S.L.Everist 5384) | Sedge |
| 1402 | Rostellularia adscendens var. pogonanthera | Forb |
| 1403 | Rostraria cristata | Tussock grass |
| 1404 | Rostraria pumila | Tussock grass |
| 1405 | Rubus anglocandicans | Forb |
| 1406 | Rulingia loxophylla | Forb |
| 1407 | Rutidosis helichrysoides subsp. helichrysoides | Forb |
| 1408 | Rytidosperma caespitosum | Tussock grass |
| 1409 | Rytidosperma erianthum | Tussock grass |
| 1410 | Rytidosperma geniculatum | Tussock grass |
| 1411 | Rytidosperma setaceum | Tussock grass |
| 1412 | Sacciolepis myosuroides | Tussock grass |
| 1413 | Salsola australis | Chenopod |
| 1414 | Salsola sp. | Chenopod |
| 1415 | Salsola tragus | Chenopod |
| 1416 | Salsola tragus subsp. tragus | Chenopod |
| 1417 | Salvia verbenaca | Forb |
| 1418 | Santalum acuminatum | Shrub |
| 1419 | Santalum lanceolatum | Shrub |
| 1420 | Santalum spicatum | Shrub |
| 1421 | Sarcostemma viminale | Shrub |
| 1422 | Sarcostemma viminale subsp. australis | Shrub |
| 1423 | Sarcostemma viminale subsp. brunonianum | Vine |
| 1424 | Sarcozona praecox | Forb |
| 1425 | Sarga plumosum | Tussock grass |
| 1426 | Sarga sp. | Tussock grass |
| 1427 | Sarga timorense | Tussock grass |
| 1428 | Sauropus glaucus | Forb |
| 1429 | Sauropus hubbardii | Forb |
| 1430 | Sauropus trachyspermus | Shrub |
| 1431 | Scaevola albida | Forb |
| 1432 | Scaevola amblyanthera var. centralis | Forb |
| 1433 | Scaevola crassifolia | Forb |
| 1434 | Scaevola depauperata | Forb |
| 1435 | Scaevola humilis | Forb |
| 1436 | Scaevola parvibarbata | Forb |
| 1437 | Scaevola parvifolia | Shrub |
| 1438 | Scaevola parvifolia subsp. parvifolia | Forb |
| 1439 | Scaevola parvifolia subsp. pilbarae | Forb |
| 1440 | Scaevola restiacea | Forb |
| 1441 | Scaevola spinescens | Forb |
| 1442 | Schizachyrium fragile | Tussock grass |
| 1443 | Schizachyrium pachyarthron | Tussock grass |
| 1444 | Schizachyrium pseudeulalia | Tussock grass |
| 1445 | Schoenia ayersii | Forb |
| 1446 | Schoenus hexandrus | Sedge |
| 1447 | Schoenus subaphyllus | Sedge |
| 1448 | Scleranthus pungens | Forb |
| 1449 | Scleria brownii | Sedge |
| 1450 | Scleria rugosa | Sedge |
| 1451 | Scleria sphacelata | Sedge |
| 1452 | Sclerolaena diacantha | Chenopod |
| 1453 | Sclerolaena articulata | Chenopod |
| 1454 | Sclerolaena bicornis | Chenopod |
| 1455 | Sclerolaena brachyptera | Chenopod |
| 1456 | Sclerolaena brevifolia | Chenopod |
| 1457 | Sclerolaena calcarata | Chenopod |
| 1458 | Sclerolaena constricta | Chenopod |
| 1459 | Sclerolaena convexula | Chenopod |
| 1460 | Sclerolaena cornishiana | Chenopod |
| 1461 | Sclerolaena costata | Chenopod |
| 1462 | Sclerolaena cuneata | Chenopod |
| 1463 | Sclerolaena decurrens | Chenopod |
| 1464 | Sclerolaena deserticola | Chenopod |
| 1465 | Sclerolaena diacantha | Chenopod |
| 1466 | Sclerolaena divaricata | Chenopod |
| 1467 | Sclerolaena drummondii | Chenopod |
| 1468 | Sclerolaena eriacantha | Chenopod |
| 1469 | Sclerolaena fontinalis | Chenopod |
| 1470 | Sclerolaena fusiformis | Chenopod |
| 1471 | Sclerolaena glabra | Chenopod |
| 1472 | Sclerolaena holtiana | Chenopod |
| 1473 | Sclerolaena intricata | Chenopod |
| 1474 | Sclerolaena johnsonii | Chenopod |
| 1475 | Sclerolaena lanicuspis | Chenopod |
| 1476 | Sclerolaena limbata | Chenopod |
| 1477 | Sclerolaena longicuspis | Chenopod |
| 1478 | Sclerolaena obliquicuspis | Chenopod |
| 1479 | Sclerolaena parallelicuspis | Chenopod |
| 1480 | Sclerolaena patenticuspis | Chenopod |
| 1481 | Sclerolaena sp. | Chenopod |
| 1482 | Sclerolaena stelligera | Chenopod |
| 1483 | Sclerolaena tetracuspis | Chenopod |
| 1484 | Sclerolaena tricuspis | Chenopod |
| 1485 | Sclerolaena uniflora | Chenopod |
| 1486 | Sclerolaena ventricosa | Chenopod |
| 1487 | Sebastiania chamaelea | Forb |
| 1488 | Sehima nervosum | Tussock grass |
| 1489 | Senecio anethifolius subsp. brevibracteolatus | Forb |
| 1490 | Senecio cunninghamii | Forb |
| 1491 | Senecio gregorii | Forb |
| 1492 | Senecio lacustrinus | Forb |
| 1493 | Senecio magnificus | Forb |
| 1494 | Senecio quadridentatus | Forb |
| 1495 | Senecio runcinifolius | Forb |
| 1496 | Senecio sp. | Forb |
| 1497 | Senecio spanomerus | Forb |
| 1498 | Senna artemisioides | Shrub |
| 1499 | Senna artemisioides subsp. petiolaris | Shrub |
| 1500 | Senna artemisioides subsp. filifolia | Shrub |
| 1501 | Senna artemisioides subsp. x sturtii | Shrub |
| 1502 | Senna artemisioides subsp. artemisioides | Shrub |
| 1503 | Senna artemisioides subsp. quadrifolia | Shrub |
| 1504 | Senna artemisioides subsp. alicia | Shrub |
| 1505 | Senna artemisioides subsp. helmsii | Shrub |
| 1506 | Senna artemisioides subsp. indeterminate | Shrub |
| 1507 | Senna artemisioides subsp. oligophylla | Shrub |
| 1508 | Senna artemisioides subsp. zygophylla | Shrub |
| 1509 | Senna cardiosperma | Shrub |
| 1510 | Senna notabilis | Shrub |
| 1511 | Senna phyllodinea | Shrub |
| 1512 | Senna pleurocarpa | Shrub |
| 1513 | Senna sp. | Shrub |
| 1514 | Senna stowardii | Shrub |
| 1515 | Sesbania cannabina | Forb |
| 1516 | Sesbania sp. | Forb |
| 1517 | Sesuvium portulacastrum | Forb |
| 1518 | Setaria apiculata | Tussock grass |
| 1519 | Setaria constricta | Tussock grass |
| 1520 | Setaria surgens | Tussock grass |
| 1521 | Shrub | Shrub |
| 1522 | Sida intricata | Shrub |
| 1523 | Sida ammophila | Forb |
| 1524 | Sida argillacea | Forb |
| 1525 | Sida brachypoda | Forb |
| 1526 | Sida calyxhymenia | Forb |
| 1527 | Sida cordifolia | Forb |
| 1528 | Sida cunninghamii | Forb |
| 1529 | Sida ectogama | Forb |
| 1530 | Sida fibulifera | Forb |
| 1531 | Sida filiformis | Forb |
| 1532 | Sida goniocarpa | Forb |
| 1533 | Sida intricata | Forb |
| 1534 | Sida petrophila | Shrub |
| 1535 | Sida platycalyx | Forb |
| 1536 | Sida rohlenae | Forb |
| 1537 | Sida scabra | Forb |
| 1538 | Sida sp. | Forb |
| 1539 | Sida spenceriana | Forb |
| 1540 | Sida spinosa | Forb |
| 1541 | Sida spodochroma | Forb |
| 1542 | Sida trichopoda | Forb |
| 1543 | Sida virgata | Forb |
| 1544 | Sisymbrium erysimoides | Forb |
| 1545 | Solanum centrale | Shrub |
| 1546 | Solanum chenopodinum | Shrub |
| 1547 | Solanum cleistogamum | Forb |
| 1548 | Solanum ellipticum | Forb |
| 1549 | Solanum esuriale | Forb |
| 1550 | Solanum lasiophyllum | Forb |
| 1551 | Solanum petrophilum | Shrub |
| 1552 | Solanum pugiunculiferum | Forb |
| 1553 | Solanum quadriloculatum | Forb |
| 1554 | Solanum sp. | Forb |
| 1555 | Solanum sturtianum | Forb |
| 1556 | Sonchus oleraceus | Forb |
| 1557 | Sorghum sp. | Tussock grass |
| 1558 | Sorghum plumosum | Tussock grass |
| 1559 | Sorghum timorense | Tussock grass |
| 1560 | Spartothamnella teucriiflora | Shrub |
| 1561 | Spergularia diandra | Forb |
| 1562 | Spermacoce brachystema | Forb |
| 1563 | Spermacoce dolichosperma | Forb |
| 1564 | Spermacoce erectiloba | Forb |
| 1565 | Spermacoce fabiformis | Forb |
| 1566 | Spermacoce leptoloba | Forb |
| 1567 | Spermacoce occultiseta | Forb |
| 1568 | Spermacoce sp. (Normanton R.Pullen 8839) | Forb |
| 1569 | Sporobolus actinocladus | Tussock grass |
| 1570 | Sporobolus australasicus | Tussock grass |
| 1571 | Sporobolus caroli | Tussock grass |
| 1572 | Sporobolus mitchellii | Tussock grass |
| 1573 | Sporobolus sp. | Tussock grass |
| 1574 | Sporobolus virginicus | Tussock grass |
| 1575 | Spyridium parvifolium | Shrub |
| 1576 | Spyridium phlebophyllum | Shrub |
| 1577 | Spyridium thymifolium | Shrub |
| 1578 | Stackhousia intermedia | Forb |
| 1579 | Stackhousia muricata | Forb |
| 1580 | Stemodia florulenta | Forb |
| 1581 | Stemodia glabella | Forb |
| 1582 | Stenanthemum stipulosum | Shrub |
| 1583 | Stenopetalum anfractum | Forb |
| 1584 | Stenopetalum lineare | Forb |
| 1585 | Stenopetalum nutans | Forb |
| 1586 | Stipa scabra | Tussock grass |
| 1587 | Stipa stuposa | Tussock grass |
| 1588 | Streptoglossa adscendens | Forb |
| 1589 | Streptoglossa bubakii | Forb |
| 1590 | Stylidium arenicola | Shrub |
| 1591 | Stylidium dielsianum | Forb |
| 1592 | Stylidium humphreysii | Forb |
| 1593 | Stylidium repens | Forb |
| 1594 | Stylobasium spathulatum | Shrub |
| 1595 | Stylosanthes hamata | Forb |
| 1596 | Stylosanthes scabra | Forb |
| 1597 | Swainsona affinis | Forb |
| 1598 | Swainsona burkei | Forb |
| 1599 | Swainsona campylantha | Forb |
| 1600 | Swainsona flavicarinata | Forb |
| 1601 | Swainsona laxa | Forb |
| 1602 | Swainsona oligophylla | Forb |
| 1603 | Swainsona phacoides | Forb |
| 1604 | Swainsona stipularis | Forb |
| 1605 | Swainsona tenuis | Forb |
| 1606 | Synaphea spinulosa | Shrub |
| 1607 | Synaptantha tillaeacea | Forb |
| 1608 | Syzygium suborbiculare | Tree |
| 1609 | Tacca leontopetaloides | Forb |
| 1610 | Tecticornia cupuliformis | Chenopod |
| 1611 | Tecticornia disarticulata | Chenopod |
| 1612 | Tecticornia halocnemoides subsp. tenuis | Chenopod |
| 1613 | Tecticornia indica | Chenopod |
| 1614 | Tecticornia indica subsp. bidens | Chenopod |
| 1615 | Tecticornia indica subsp. indica | Chenopod |
| 1616 | Tecticornia lylei | Chenopod |
| 1617 | Tecticornia pergranulata subsp. queenslandica | Chenopod |
| 1618 | Tecticornia sp. | Chenopod |
| 1619 | Tecticornia tenuis | Chenopod |
| 1620 | Templetonia aculeata | Shrub |
| 1621 | Templetonia ceracea | Shrub |
| 1622 | Templetonia egena | Shrub |
| 1623 | Templetonia rossii | Shrub |
| 1624 | Tephrosia coriacea | Forb |
| 1625 | Tephrosia delestangii | Forb |
| 1626 | Tephrosia filipes | Forb |
| 1627 | Tephrosia leptoclada | Forb |
| 1628 | Tephrosia remotiflora | Forb |
| 1629 | Tephrosia sp. | Forb |
| 1630 | Tephrosia sphaerospora | Forb |
| 1631 | Tephrosia supina | Forb |
| 1632 | Terminalia aridicola | Tree |
| 1633 | Terminalia aridicola subsp. aridicola | Tree |
| 1634 | Terminalia bursarina | Shrub |
| 1635 | Terminalia canescens | Shrub |
| 1636 | Terminalia carpentariae | Tree |
| 1637 | Terminalia ferdinandiana | Tree |
| 1638 | Terminalia oblongata subsp. volucris | Tree |
| 1639 | Terminalia platyphylla | Tree |
| 1640 | Terminalia platyptera | Tree |
| 1641 | Terminalia pterocarya | Shrub |
| 1642 | Terminalia subacroptera | Tree |
| 1643 | Terminalia volucris | Tree |
| 1644 | Tetragonia eremaea | Forb |
| 1645 | Tetragonia implexicoma | Forb |
| 1646 | Tetragonia moorei | Forb |
| 1647 | Tetragonia sp. | Forb |
| 1648 | Tetratheca efoliata | Shrub |
| 1649 | Tetratheca pilosa subsp. pilosa | Shrub |
| 1650 | Tetratheca pilosa var. pilosa | Shrub |
| 1651 | Teucrium racemosum | Forb |
| 1652 | Thaumastochloa brassii | Tussock grass |
| 1653 | Thaumastochloa major | Tussock grass |
| 1654 | Thaumastochloa pubescens | Tussock grass |
| 1655 | Thelymitra antennifera | Forb |
| 1656 | Themeda avenacea | Tussock grass |
| 1657 | Themeda quadrivalvis | Tussock grass |
| 1658 | Themeda triandra | Tussock grass |
| 1659 | Thomasia petalocalyx | Tussock grass |
| 1660 | Threlkeldia diffusa | Tussock grass |
| 1661 | Thryptomene kochii | Shrub |
| 1662 | Thryptomene urceolaris | Shrub |
| 1663 | Thyridolepis mitchelliana | Tussock grass |
| 1664 | Thyridolepis xerophila | Tussock grass |
| 1665 | Thysanotus sp. | Forb |
| 1666 | Tinospora smilacina | Vine |
| 1667 | Trachymene glaucifolia | Forb |
| 1668 | Tragus australianus | Tussock grass |
| 1669 | Trianthema pilosa | Forb |
| 1670 | Trianthema rhynchocalyptra | Forb |
| 1671 | Trianthema triquetra | Forb |
| 1672 | Tribulopis pentandra | Forb |
| 1673 | Tribulus eichlerianus | Forb |
| 1674 | Tribulus hystrix | Forb |
| 1675 | Tribulus minutus | Forb |
| 1676 | Tribulus sp. | Forb |
| 1677 | Trichanthodium skirrophorum | Forb |
| 1678 | Trichodesma zeylanicum | Forb |
| 1679 | Trifolium angustifolium | Forb |
| 1680 | Trifolium striatum | Forb |
| 1681 | Trigonella suavissima | Forb |
| 1682 | Triodia rigidissima | Hummock grass |
| 1683 | Triodia basedowii | Hummock grass |
| 1684 | Triodia bitextura | Hummock grass |
| 1685 | Triodia brizoides | Hummock grass |
| 1686 | Triodia irritans | Hummock grass |
| 1687 | Triodia pungens | Hummock grass |
| 1688 | Triodia rigidissima | Hummock grass |
| 1689 | Triodia scariosa | Hummock grass |
| 1690 | Triodia schinzii | Hummock grass |
| 1691 | Triodia sp. | Hummock grass |
| 1692 | Triodia tomentosa | Hummock grass |
| 1693 | Tripogon loliiformis | Tussock grass |
| 1694 | Triraphis mollis | Tussock grass |
| 1695 | Triumfetta plumigera | Forb |
| 1696 | Triumfetta sp. | Forb |
| 1697 | Ulex europaeus | Shrub |
| 1698 | Uranthoecium truncatum | Tussock grass |
| 1699 | Uraria lagopodioides | Forb |
| 1700 | Urochloa holosericea | Tussock grass |
| 1701 | Urochloa ramosa | Tussock grass |
| 1702 | Urochloa subquadripara | Tussock grass |
| 1703 | Vachellia farnesiana | Shrub |
| 1704 | Vachellia sutherlandii | Shrub |
| 1705 | Ventilago viminalis | Tree |
| 1706 | Verbena officinalis | Forb |
| 1707 | Verticordia chrysantha | Shrub |
| 1708 | Verticordia eriocephala | Shrub |
| 1709 | Verticordia helmsii | Shrub |
| 1710 | Verticordia inclusa | Shrub |
| 1711 | Verticordia picta | Shrub |
| 1712 | Verticordia pritzelii | Shrub |
| 1713 | Vigna lanceolata | Forb |
| 1714 | Vigna sp. (McDonald Downs Station R.A.Perry 3416) | Vine |
| 1715 | Vigna vexillata var. angustifolia | Vine |
| 1716 | Vittadinia cervicularis | Forb |
| 1717 | Vittadinia cuneata subsp. cuneata | Forb |
| 1718 | Vittadinia cuneata | Forb |
| 1719 | Vittadinia eremaea | Forb |
| 1720 | Vittadinia gracilis | Forb |
| 1721 | Vittadinia nullarborensis | Forb |
| 1722 | Vittadinia sp. | Forb |
| 1723 | Vittadinia sulcata | Forb |
| 1724 | Vulpia bromoides | Forb |
| 1725 | Vulpia muralis | Forb |
| 1726 | Vulpia myuros f. myuros | Forb |
| 1727 | Wahlenbergia sp. | Forb |
| 1728 | Wahlenbergia tumidifructa | Forb |
| 1729 | Waitzia acuminata var. acuminata | Forb |
| 1730 | Waltheria indica | Forb |
| 1731 | Westringia cephalantha | Forb |
| 1732 | Westringia rigida | Shrub |
| 1733 | Wrightia saligna | Shrub |
| 1734 | Wurmbea biglandulosa subsp. flindersii | Forb |
| 1735 | Wurmbea sp. Great Victoria Desert | Forb |
| 1736 | Xanthium occidentale | Forb |
| 1737 | Xanthoparmelia semiviridis | Forb |
| 1738 | Xanthorrhoea quadrangulata | Shrub |
| 1739 | Xanthorrhoea semiplana | Shrub |
| 1740 | Xanthorrhoea semiplana subsp. semiplana | Shrub |
| 1741 | Xanthorrhoea sp. | Shrub |
| 1742 | Xanthostemon paradoxus | Tree |
| 1743 | Xerochloa imberbis | Tussock grass |
| 1744 | Xerochrysum bracteatum | Forb |
| 1745 | Yakirra australiensis | Tussock grass |
| 1746 | Yakirra majuscula | Tussock grass |
| 1747 | Ziziphus mauritiana | Tree |
| 1748 | Zornia adenophora | Forb |
| 1749 | Zornia albiflora | Forb |
| 1750 | Zornia muriculata | Forb |
| 1751 | Zornia muriculata subsp. angustata | Forb |
| 1752 | Zornia prostrata | Forb |
| 1753 | Zygochloa paradoxa | Hummock grass |
| 1754 | Zygophyllum apiculatum | Forb |
| 1755 | Zygophyllum aurantiacum | Forb |
| 1756 | Zygophyllum aurantiacum subsp. aurantiacum | Forb |
| 1757 | Zygophyllum billardierei | Forb |
| 1758 | Zygophyllum emarginatum | Forb |
| 1759 | Zygophyllum eremaeum | Forb |
| 1760 | Zygophyllum glaucum | Forb |
| 1761 | Zygophyllum howittii | Forb |
| 1762 | Zygophyllum iodocarpum | Forb |
| 1763 | Zygophyllum iodocarpum | Forb |
| 1764 | Zygophyllum ovatum | Forb |
| 1765 | Zygophyllum prismatothecum | Forb |
| 1766 | Zygophyllum simile | Forb |
| 1767 | Zygophyllum sp. | Forb |
|  |  |  |
