# Supplementary material for: Floristic and structural assessment of Australian rangeland vegetation with standardized plot-based surveys
Source: PLoS One. 2018 Sep 7;13(9):e0202073. doi: 10.1371/journal.pone.0202073 (PMC6128463; doi:10.1371/journal.pone.0202073)
Supplement: S6 Table — The entire data set and for each supercluster. Correlation coefficients > 0.700 are highlighted. (DOCX) [file pone.0202073.s006.docx]

S6 Table- Pearson correlation coefficients between the CCA ordination scores and the environmental variables.

Highlighted in bold are correlation coefficients > 0.700

|  | **ALL SUPERCLUSTERS** | | **MEDITERRANEAN SUPERCLUSTER** | | **SAVANNA SUPERCLUSTER** | | **DESERT SUPERCLUSTER** | |
| --- | --- | --- | --- | --- | --- | --- | --- | --- |
| Variables | Axis 1 | Axis 2 | Axis 1 | Axis 2 | Axis 1 | Axis 2 | Axis 1 | Axis 2 |
|  |  |  |  |  |  |  |  |  |
| ARIDITY | 0.540 | **0.776** | **0.958** | -0.031 | -0.475 | **-0.736** | **-0.713** | -0.301 |
| RAIN SEASON. | -0.600 | **0.750** | **0.869** | -0.273 | 0.600 | -0.370 | 0.396 | 0.420 |
| MAP | -0.125 | **0.881** | **0.953** | -0.056 | -0.346 | **-0.730** | -0.112 | -0.169 |
| MAT | **-0.889** | 0.400 | **-0.894** | 0.026 | 0.337 | -0.056 | **0.715** | 0.504 |
| PHOSPHORUS | -0.174 | 0.028 | -0.536 | 0.266 | -0.172 | -0.122 | 0.629 | -0.206 |
| NITROGEN | **0.704** | 0.504 | **0.881** | 0.043 | -0.489 | -0.344 | -0.348 | -0.529 |
| CARBON | **0.716** | 0.511 | **0.865** | 0.032 | **-0.766** | -0.452 | **-0.739** | -0.227 |
| CEC | -0.113 | -0.229 | **-0.727** | 0.206 | 0.418 | 0.503 | 0.315 | 0.026 |
| pH | 0.079 | -0.698 | **-0.955** | 0.057 | 0.329 | 0.464 | 0.224 | -0.439 |
| AWC | -0.581 | 0.300 | 0.061 | 0.399 | 0.156 | 0.401 | 0.566 | 0.321 |
| SAND | 0.168 | -0.157 | -0.093 | -0.398 | -0.484 | 0.076 | -0.283 | 0.278 |
| CLAY | -0.180 | 0.080 | -0.170 | 0.494 | 0.458 | -0.163 | 0.185 | -0.138 |
| BULK DENSITY | -0.468 | -0.068 | -0.272 | -0.168 | 0.279 | 0.478 | -0.025 | **0.761** |
| BARE SUBSTR. | -0.225 | -0.365 | -0.672 | -0.107 | 0.562 | -0.110 | 0.450 | 0.262 |
|  |  |  |  |  |  |  |  |  |

Aridity Index: represented in an inverse scale (high values indicate low aridity); Rain Seasonality: Coefficient of variation of mean annual rainfall. MAP: Mean annual precipitation; MAT: Mean annual temperature; Total Nitrogen and Phosphorus: Mass fraction of total in the soil by weight; CEC: Effective cation exchange capacity; AWC: Available water capacity; Clay & Sand percent in soil; Carbon, Organic: mass fraction by weight; Bare substrate: Number of PIs uncovered by vegetation.
