# Supplementary figures and images for: Floristic and structural assessment of Australian rangeland vegetation with standardized plot-based surveys
Source: PLoS One. 2018 Sep 7;13(9):e0202073. doi: 10.1371/journal.pone.0202073 (PMC6128463; doi:10.1371/journal.pone.0202073)

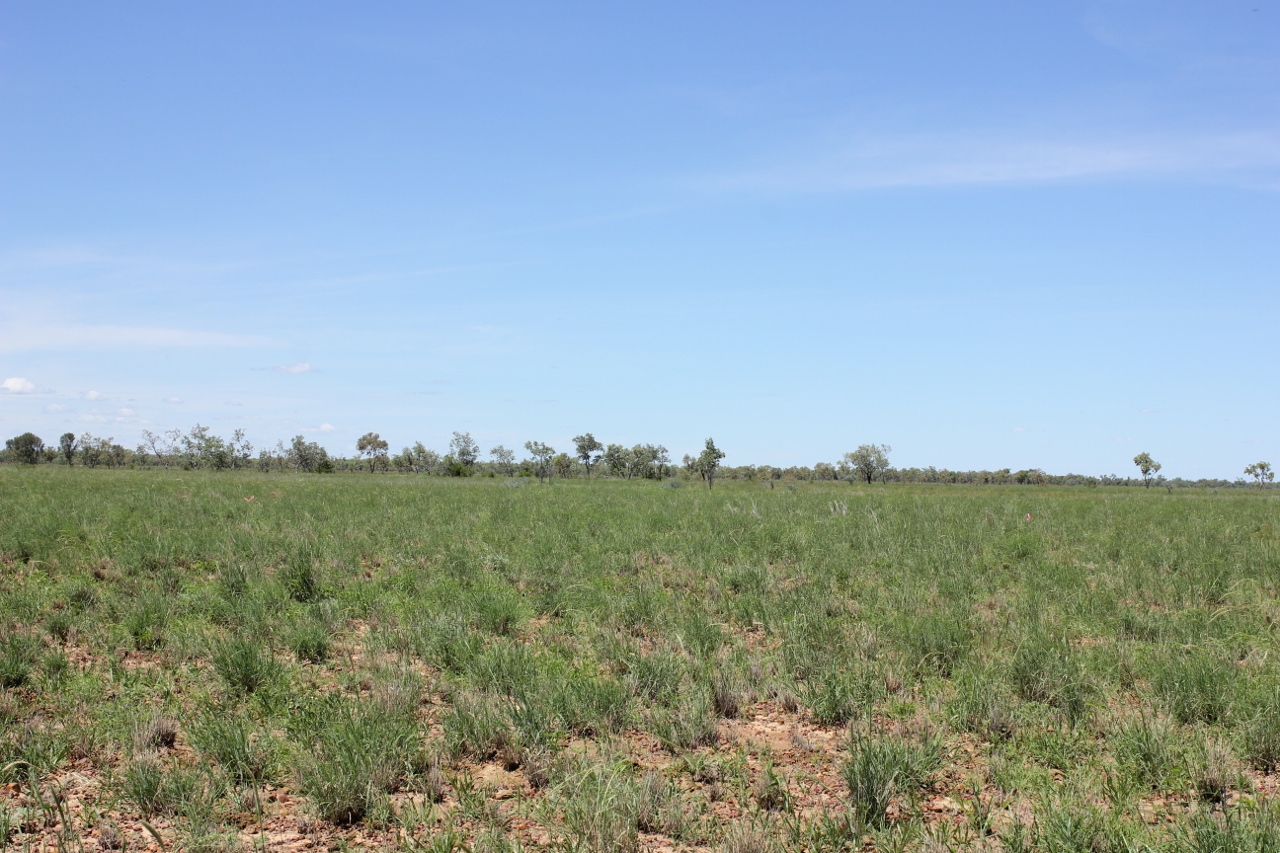

Supplement: S4 File — Mediterranean supercluster (clusters M1-M2); Savanna supercluster (clusters S1-S4); Desert supercluster (clusters D1-D5). Plot provenance is also displayed. (ZIP) [file pone.0202073.s010.zip › Cluster S2 QDAMGD0027.jpg]

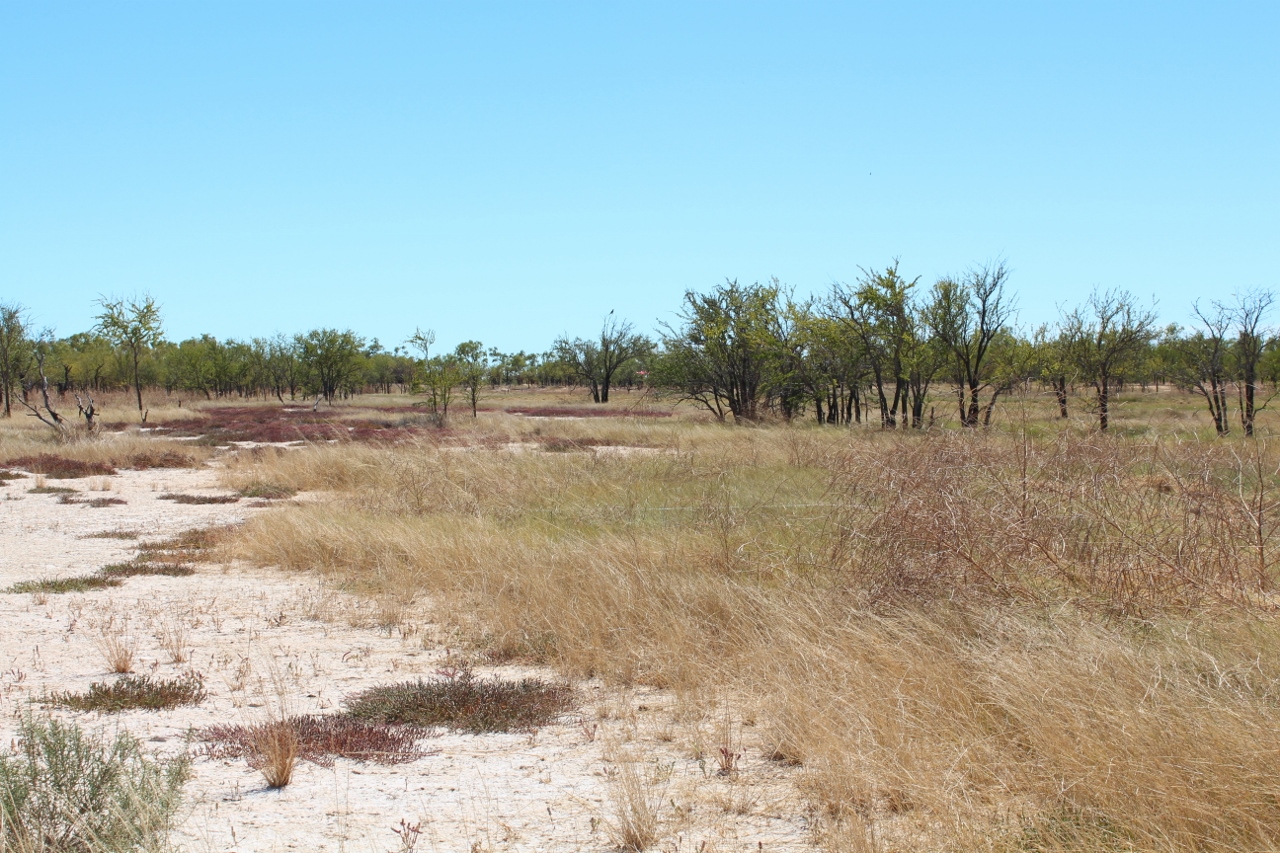

Supplement: S4 File — Mediterranean supercluster (clusters M1-M2); Savanna supercluster (clusters S1-S4); Desert supercluster (clusters D1-D5). Plot provenance is also displayed. (ZIP) [file pone.0202073.s010.zip › Cluster S3 QDAGUP0017.jpg]

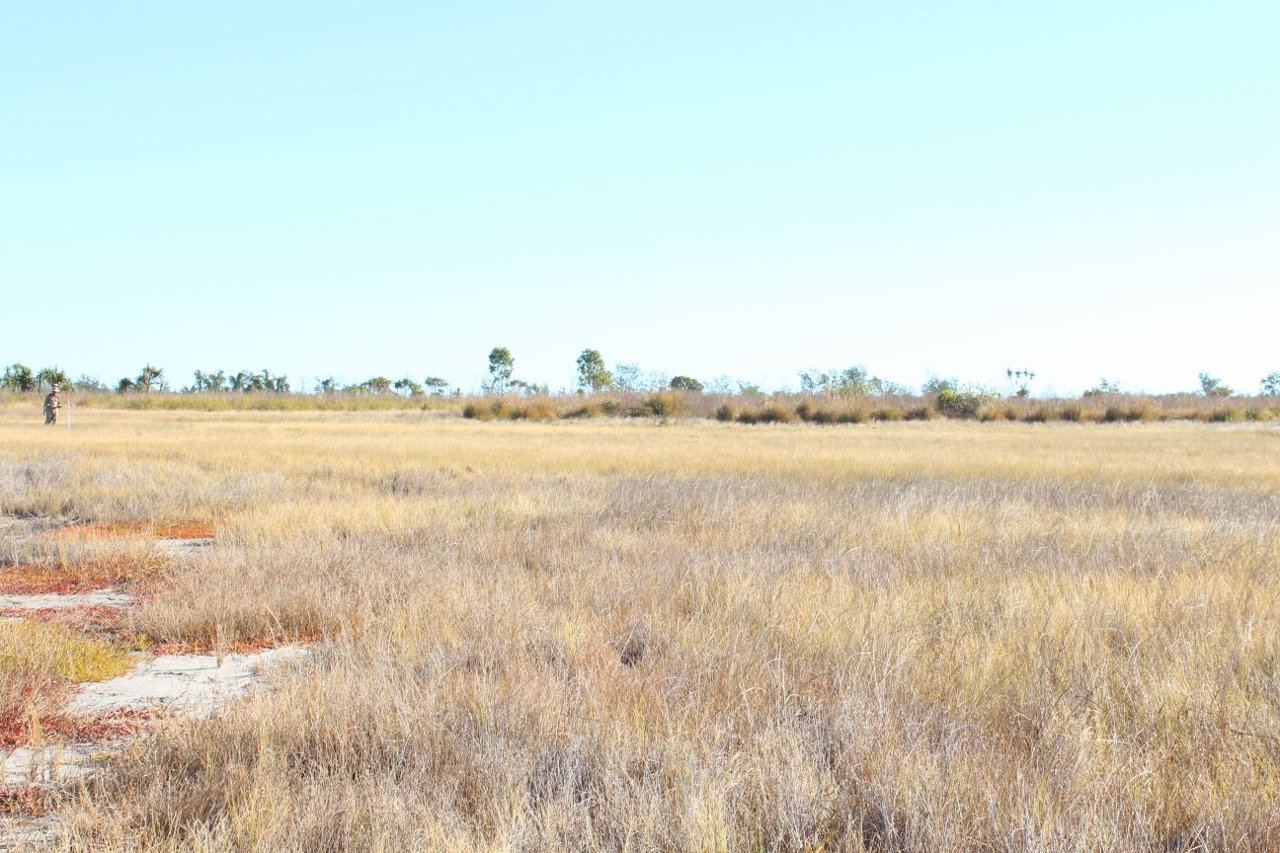

Supplement: S4 File — Mediterranean supercluster (clusters M1-M2); Savanna supercluster (clusters S1-S4); Desert supercluster (clusters D1-D5). Plot provenance is also displayed. (ZIP) [file pone.0202073.s010.zip › Cluster S3 QDAGUP0020.jpg]

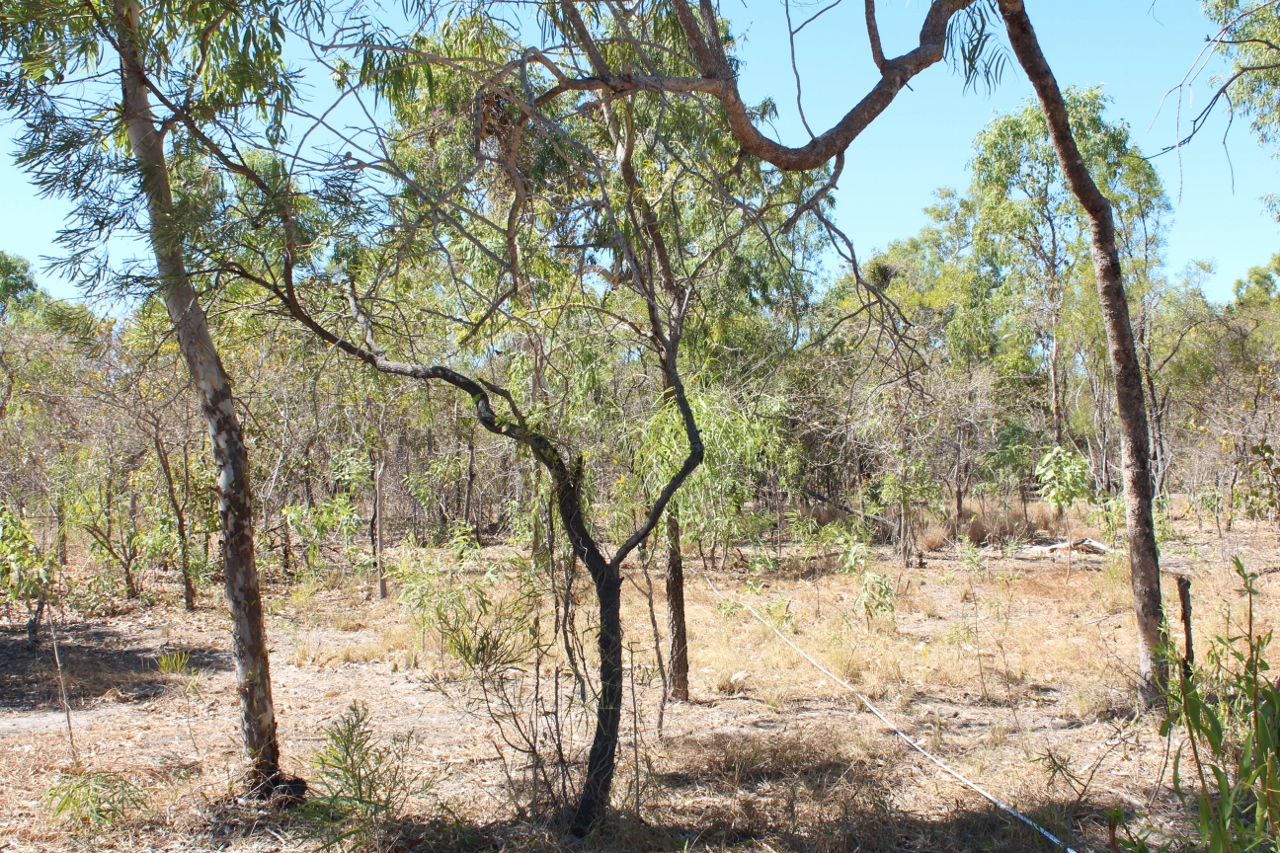

Supplement: S4 File — Mediterranean supercluster (clusters M1-M2); Savanna supercluster (clusters S1-S4); Desert supercluster (clusters D1-D5). Plot provenance is also displayed. (ZIP) [file pone.0202073.s010.zip › Cluster S3 QDAGUP0021.jpg]

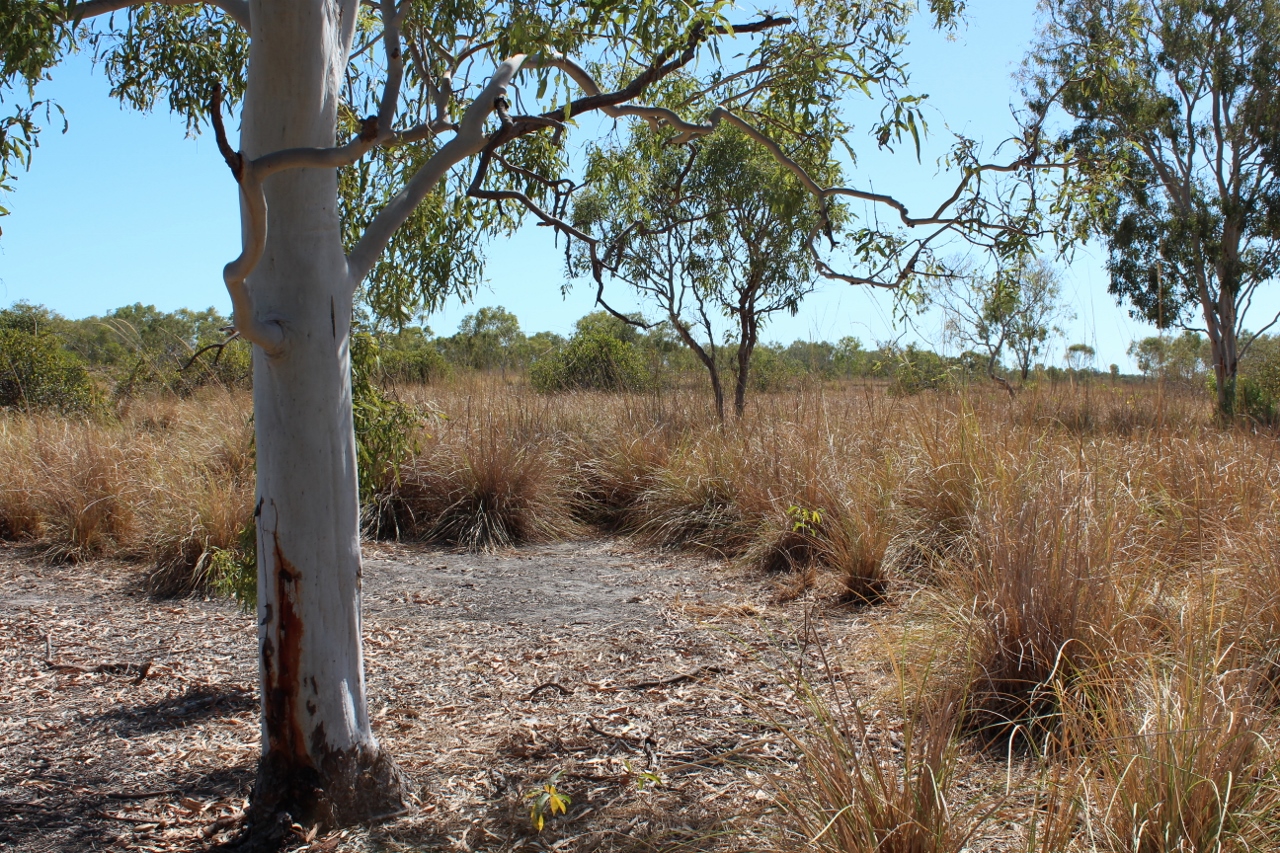

Supplement: S4 File — Mediterranean supercluster (clusters M1-M2); Savanna supercluster (clusters S1-S4); Desert supercluster (clusters D1-D5). Plot provenance is also displayed. (ZIP) [file pone.0202073.s010.zip › Cluster S3 QDAGUP0025.jpg]

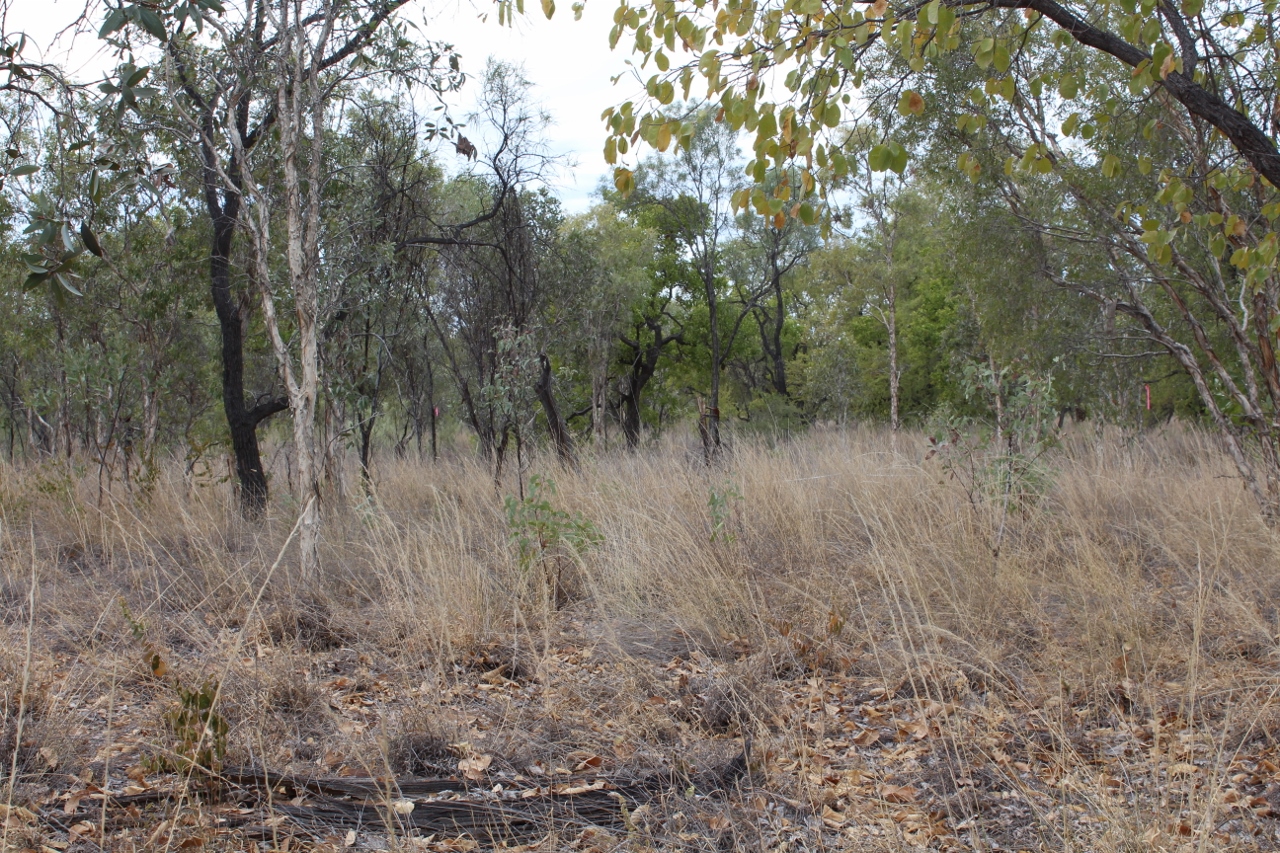

Supplement: S4 File — Mediterranean supercluster (clusters M1-M2); Savanna supercluster (clusters S1-S4); Desert supercluster (clusters D1-D5). Plot provenance is also displayed. (ZIP) [file pone.0202073.s010.zip › Cluster S4 QDAGUP0008.jpg]

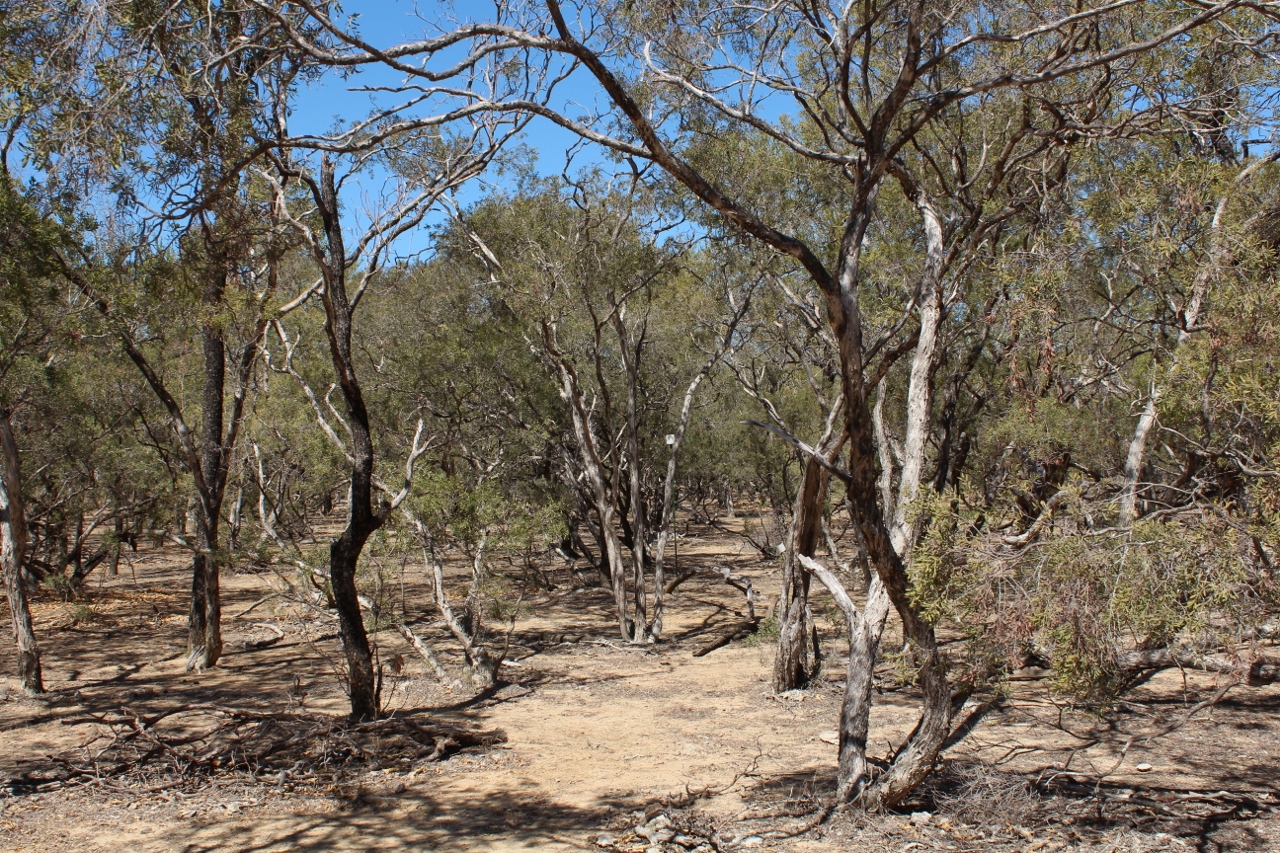

Supplement: S4 File — Mediterranean supercluster (clusters M1-M2); Savanna supercluster (clusters S1-S4); Desert supercluster (clusters D1-D5). Plot provenance is also displayed. (ZIP) [file pone.0202073.s010.zip › Cluster S4 QDAGUP0027.jpg]

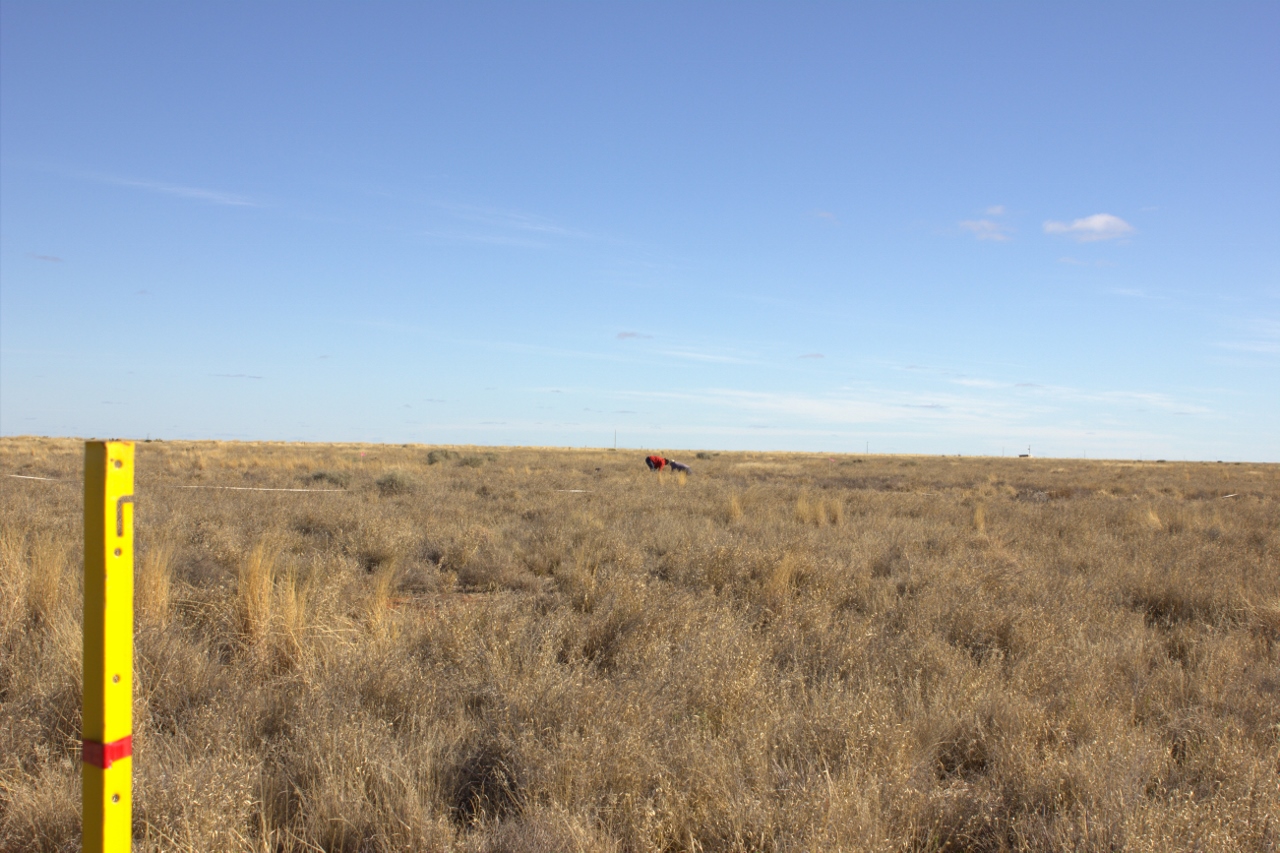

Supplement: S4 File — Mediterranean supercluster (clusters M1-M2); Savanna supercluster (clusters S1-S4); Desert supercluster (clusters D1-D5). Plot provenance is also displayed. (ZIP) [file pone.0202073.s010.zip › Cluster D1 NSABHC0005.jpg]

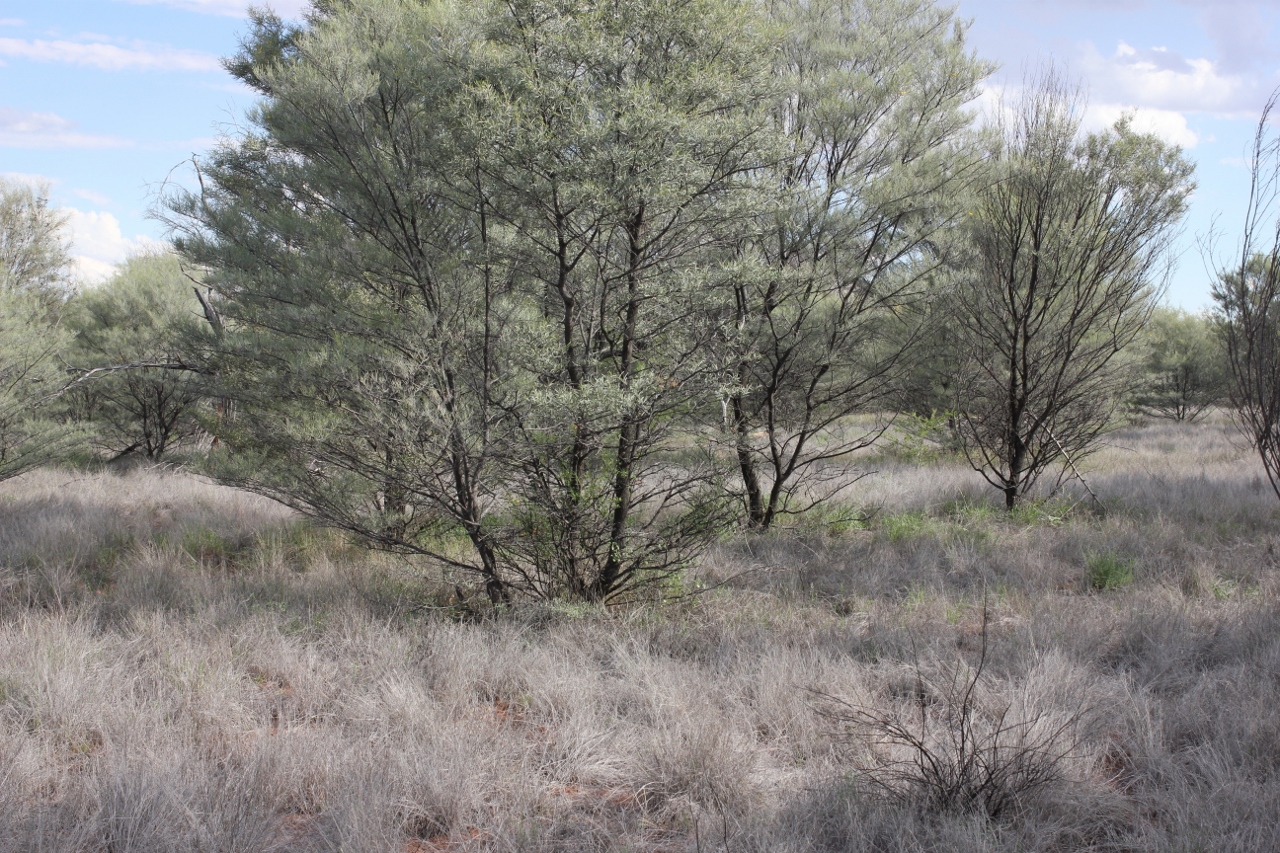

Supplement: S4 File — Mediterranean supercluster (clusters M1-M2); Savanna supercluster (clusters S1-S4); Desert supercluster (clusters D1-D5). Plot provenance is also displayed. (ZIP) [file pone.0202073.s010.zip › Cluster D1 NTAFIN0026.jpg]

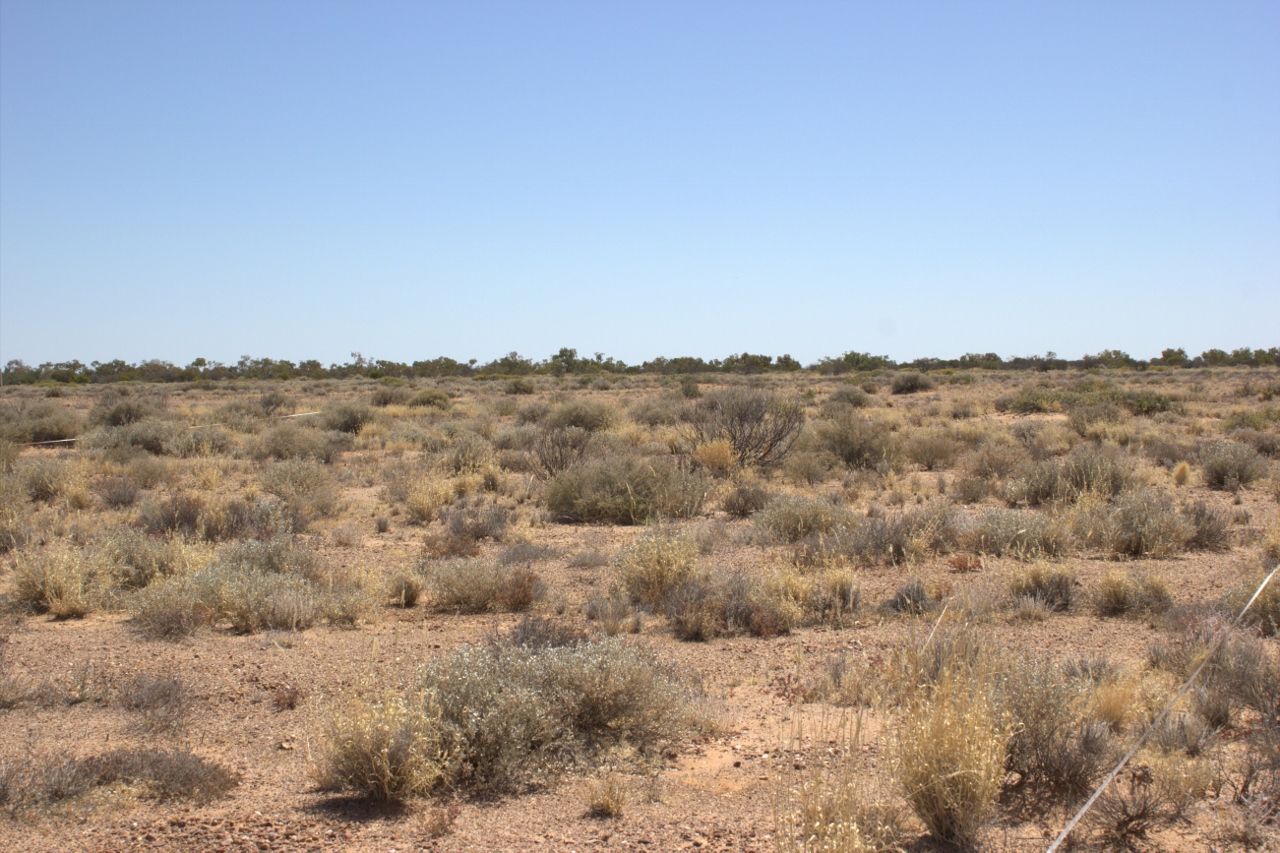

Supplement: S4 File — Mediterranean supercluster (clusters M1-M2); Savanna supercluster (clusters S1-S4); Desert supercluster (clusters D1-D5). Plot provenance is also displayed. (ZIP) [file pone.0202073.s010.zip › Cluster D1 SAASTP0019.jpg]

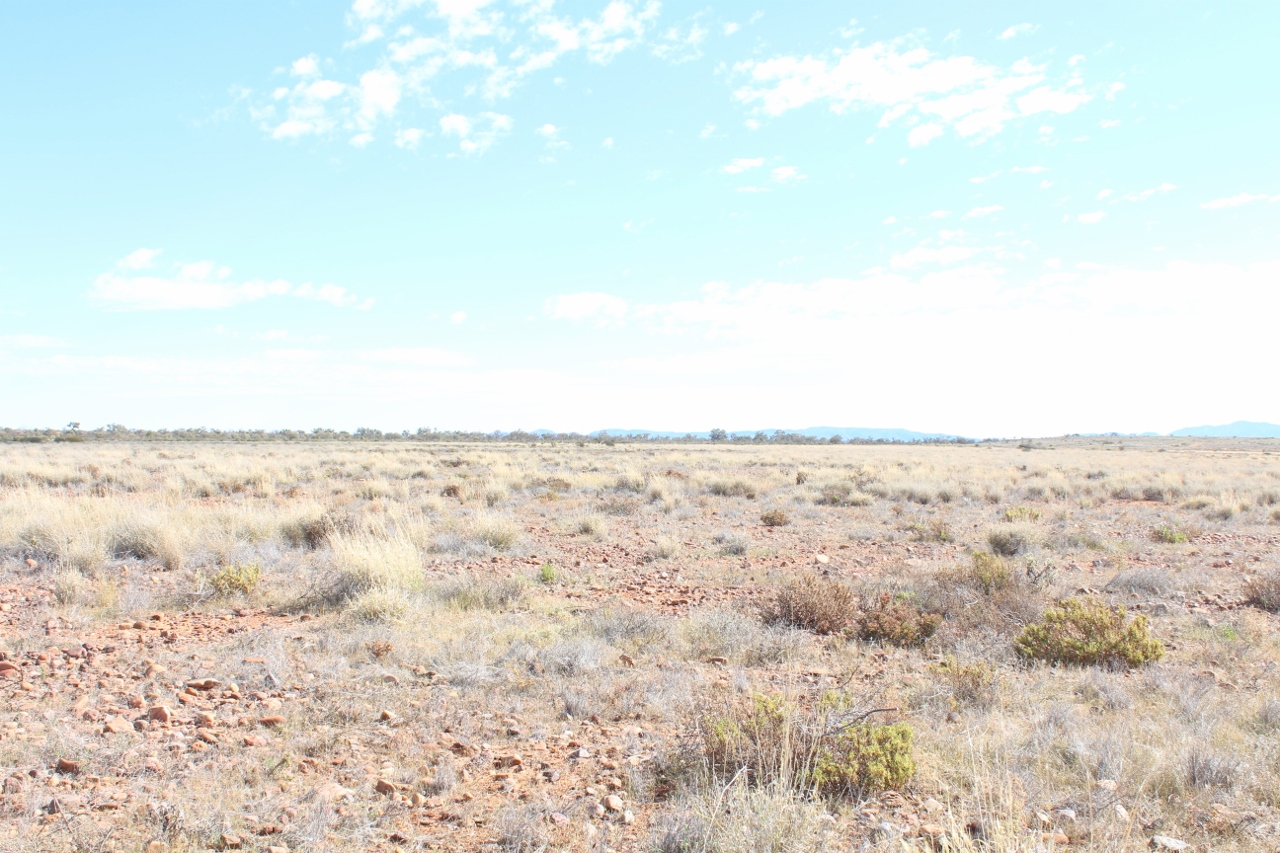

Supplement: S4 File — Mediterranean supercluster (clusters M1-M2); Savanna supercluster (clusters S1-S4); Desert supercluster (clusters D1-D5). Plot provenance is also displayed. (ZIP) [file pone.0202073.s010.zip › Cluster D1 SATSTP0001.jpg]

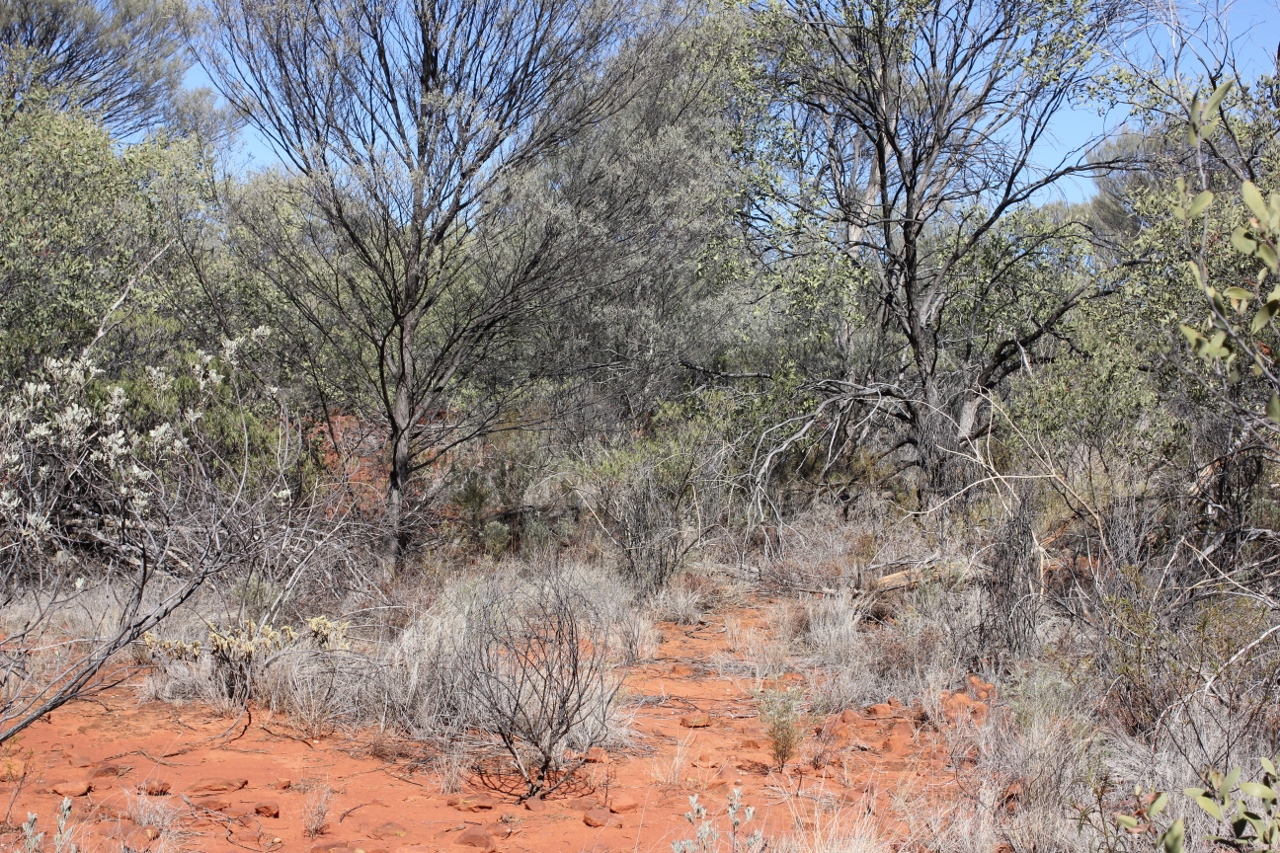

Supplement: S4 File — Mediterranean supercluster (clusters M1-M2); Savanna supercluster (clusters S1-S4); Desert supercluster (clusters D1-D5). Plot provenance is also displayed. (ZIP) [file pone.0202073.s010.zip › Cluster D2 NTAFIN0004.jpg]

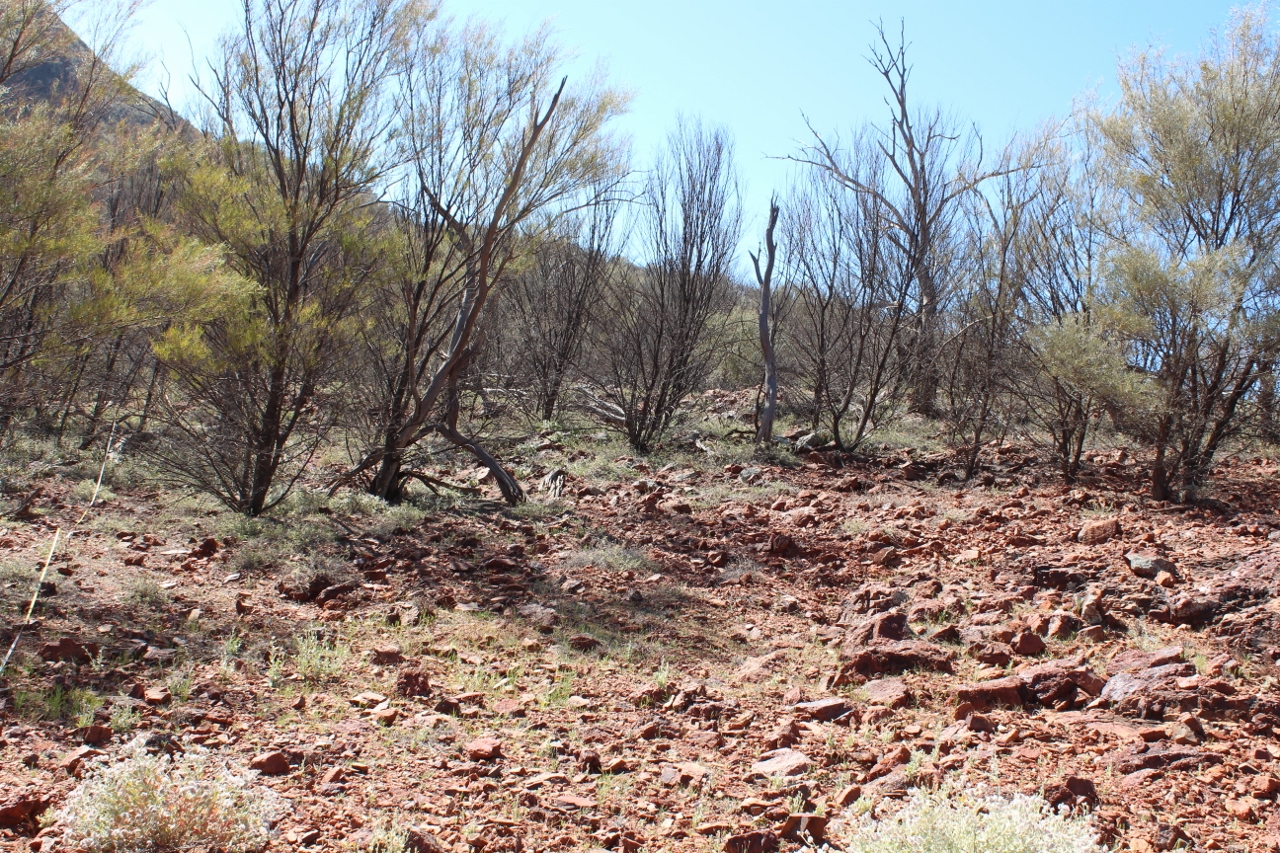

Supplement: S4 File — Mediterranean supercluster (clusters M1-M2); Savanna supercluster (clusters S1-S4); Desert supercluster (clusters D1-D5). Plot provenance is also displayed. (ZIP) [file pone.0202073.s010.zip › Cluster D2 SATFLB0017.jpg]

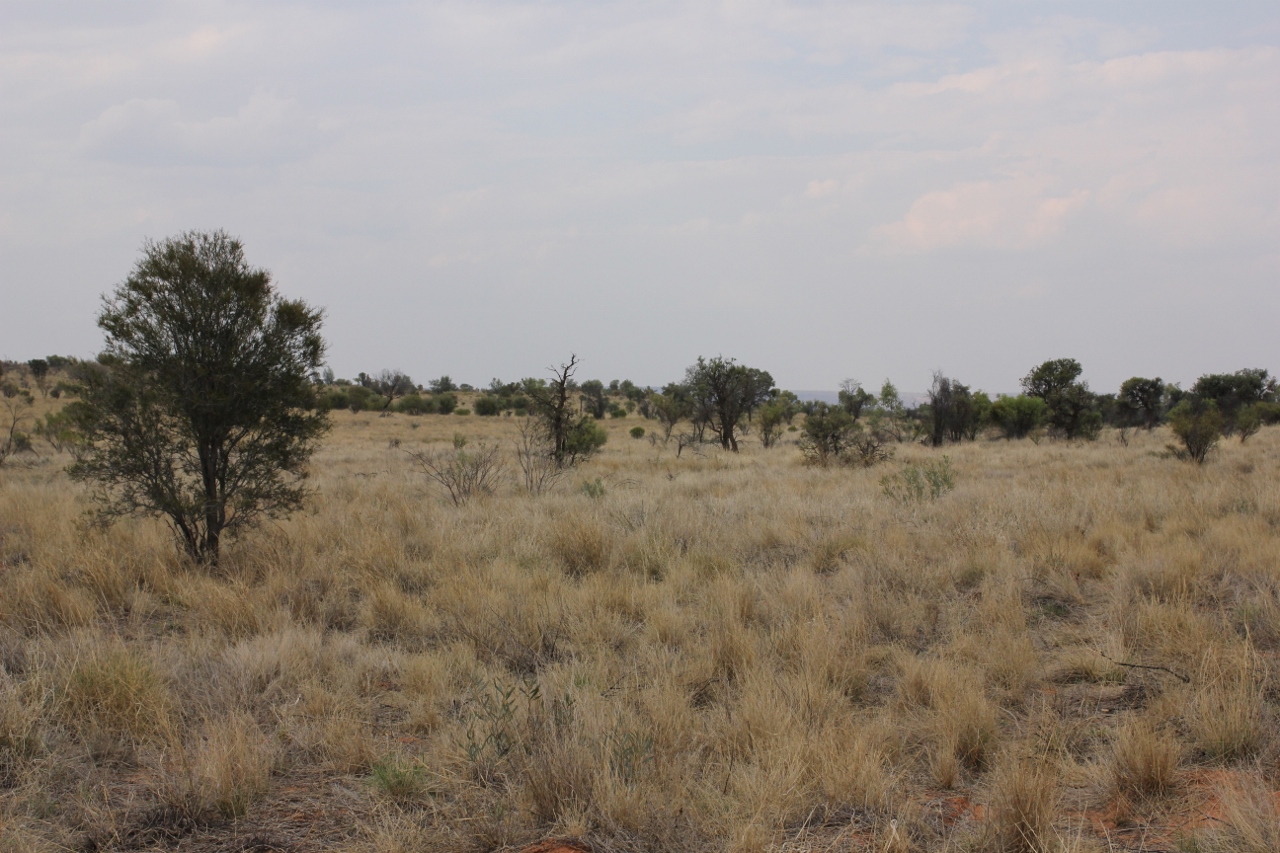

Supplement: S4 File — Mediterranean supercluster (clusters M1-M2); Savanna supercluster (clusters S1-S4); Desert supercluster (clusters D1-D5). Plot provenance is also displayed. (ZIP) [file pone.0202073.s010.zip › Cluster D3 NTAFIN0001.jpg]

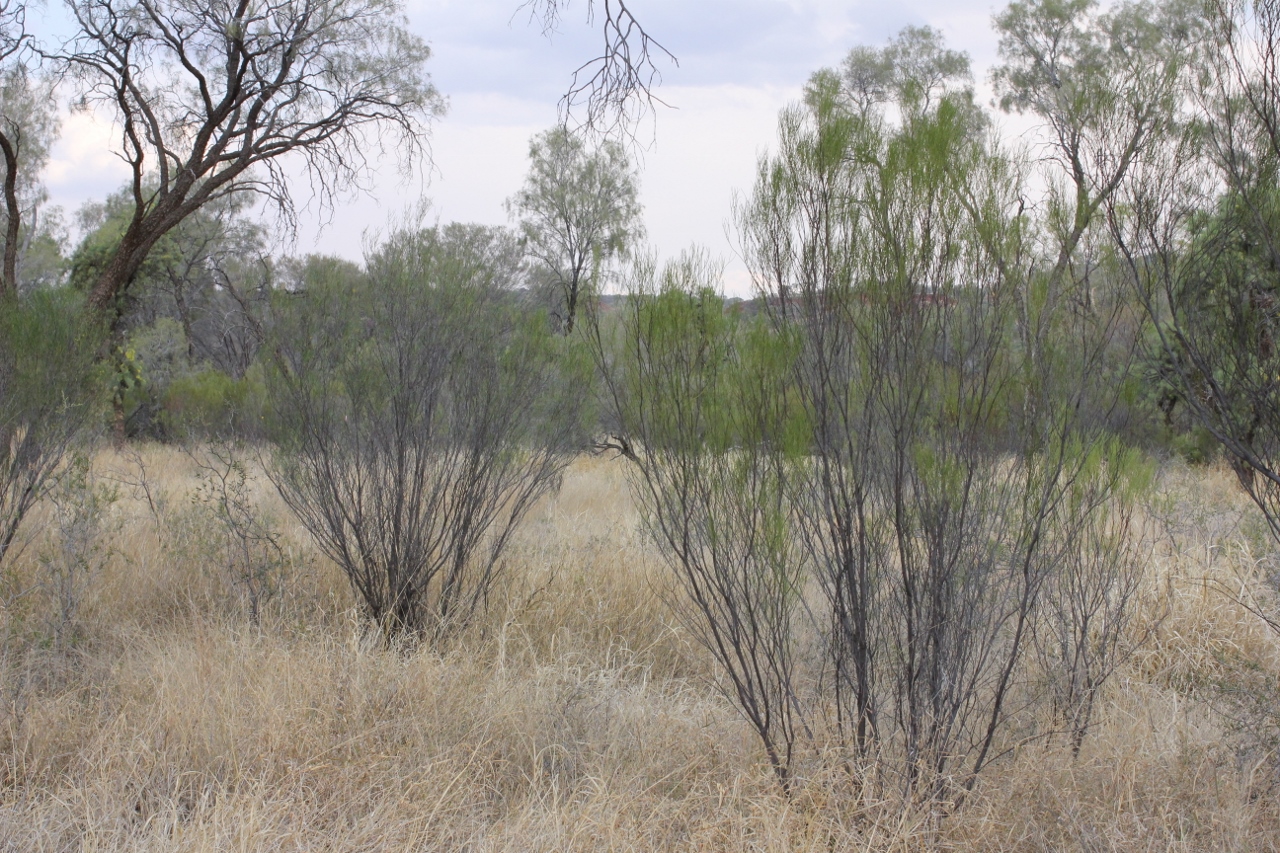

Supplement: S4 File — Mediterranean supercluster (clusters M1-M2); Savanna supercluster (clusters S1-S4); Desert supercluster (clusters D1-D5). Plot provenance is also displayed. (ZIP) [file pone.0202073.s010.zip › Cluster D3 NTAFIN0002.jpg]

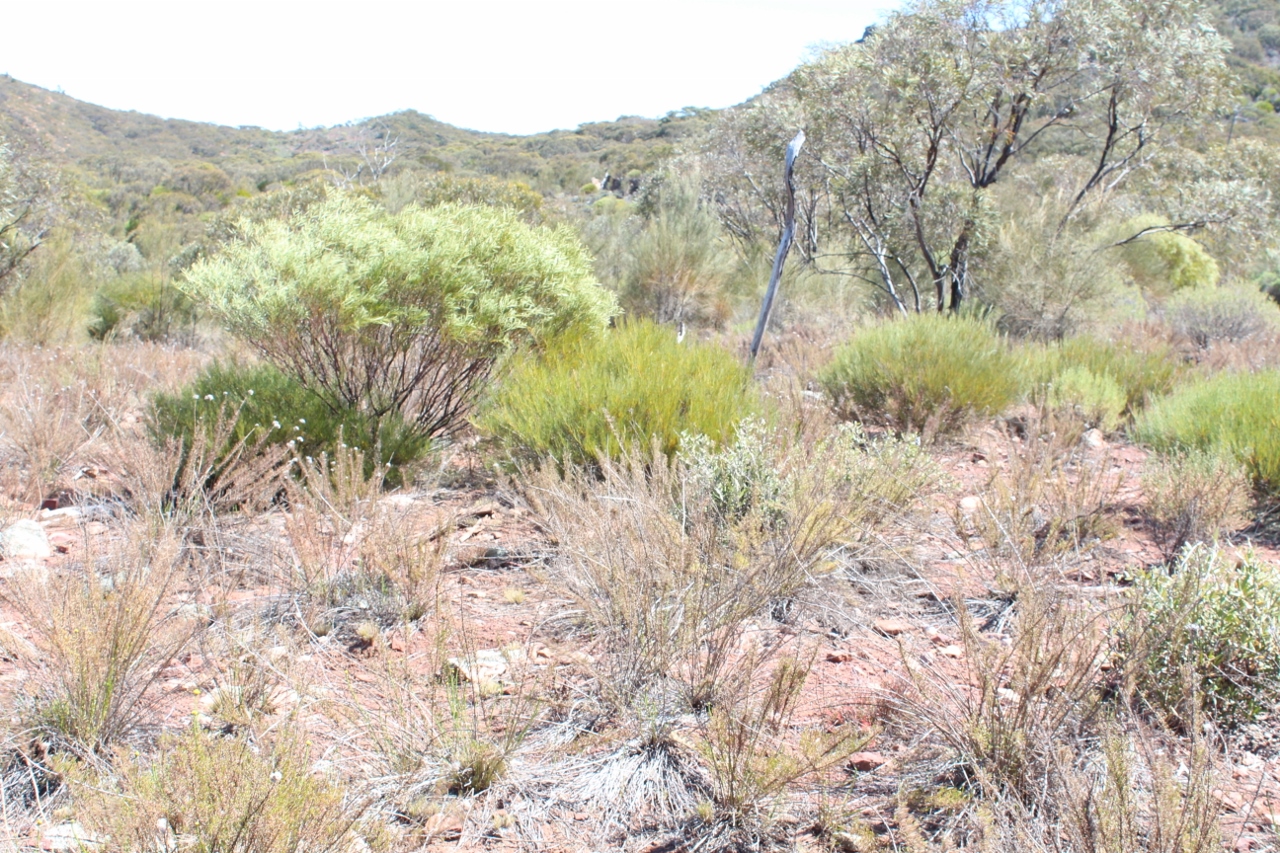

Supplement: S4 File — Mediterranean supercluster (clusters M1-M2); Savanna supercluster (clusters S1-S4); Desert supercluster (clusters D1-D5). Plot provenance is also displayed. (ZIP) [file pone.0202073.s010.zip › Cluster D4 SATFLB0006.jpg]

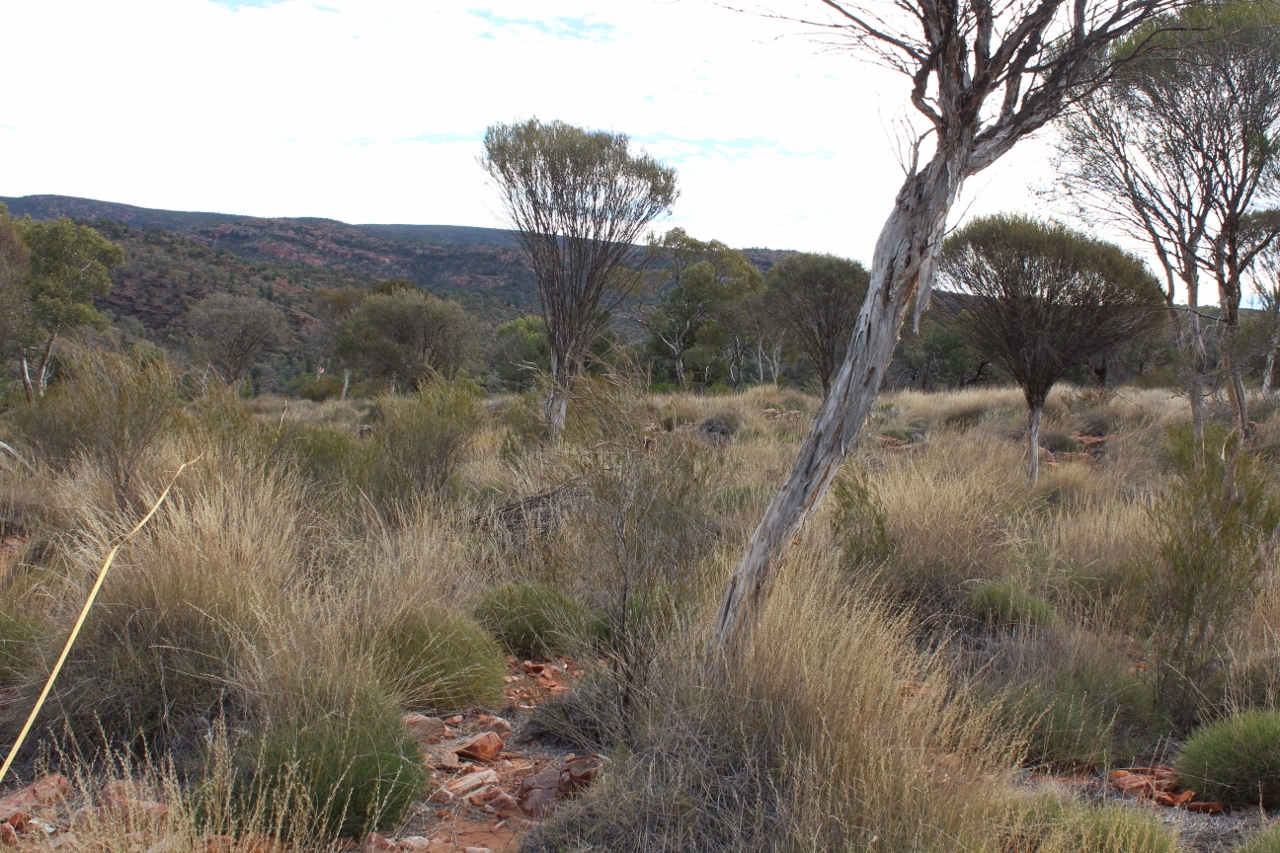

Supplement: S4 File — Mediterranean supercluster (clusters M1-M2); Savanna supercluster (clusters S1-S4); Desert supercluster (clusters D1-D5). Plot provenance is also displayed. (ZIP) [file pone.0202073.s010.zip › Cluster D4 SATFLB0021.jpg]

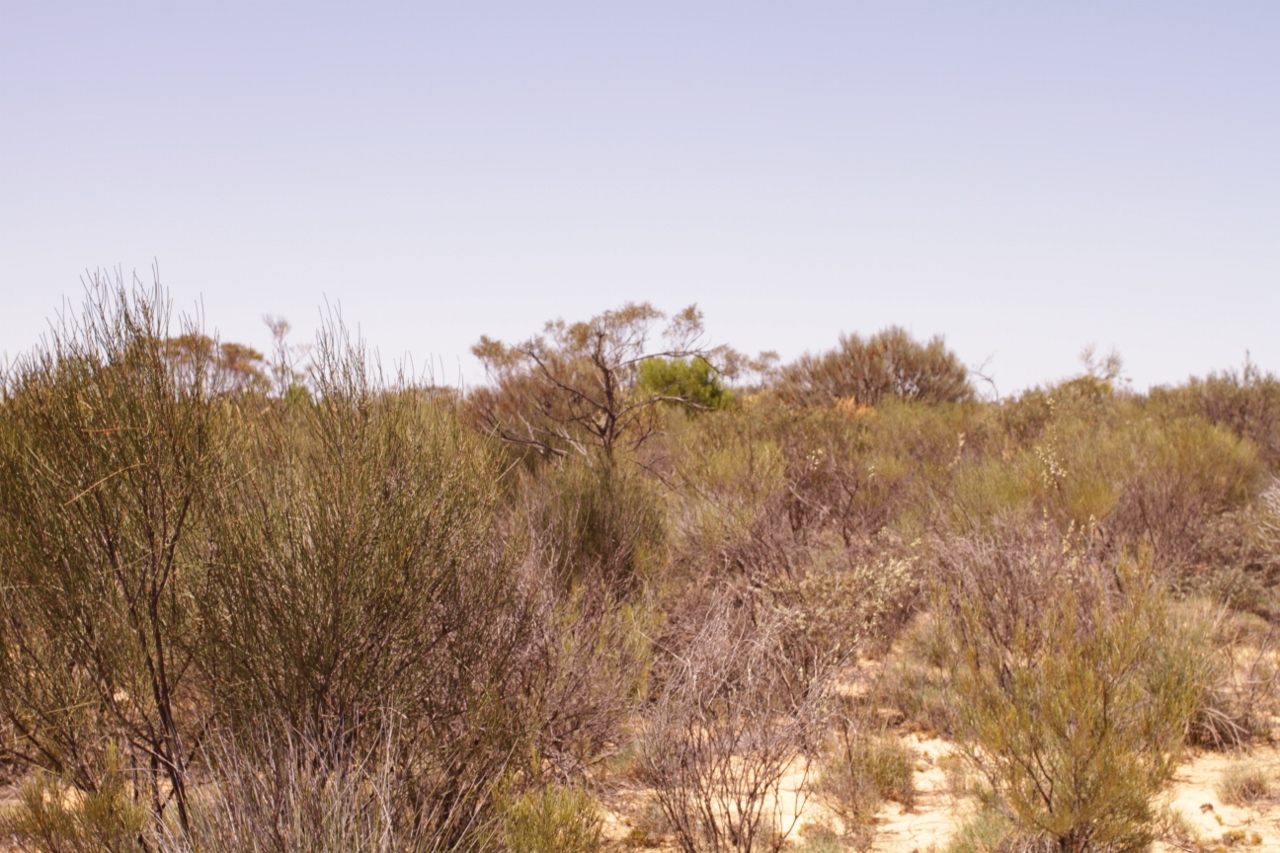

Supplement: S4 File — Mediterranean supercluster (clusters M1-M2); Savanna supercluster (clusters S1-S4); Desert supercluster (clusters D1-D5). Plot provenance is also displayed. (ZIP) [file pone.0202073.s010.zip › Cluster D5 WAACOO0017.jpg]

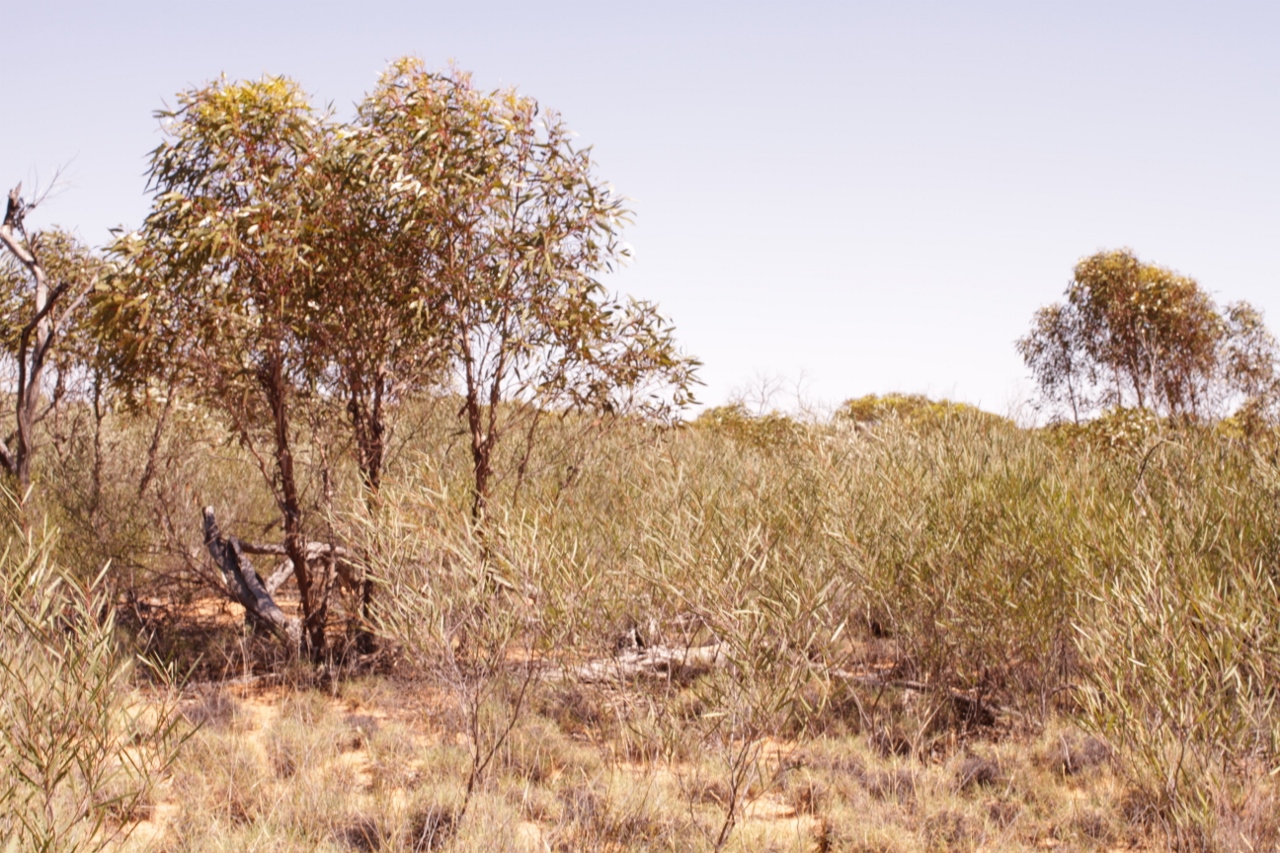

Supplement: S4 File — Mediterranean supercluster (clusters M1-M2); Savanna supercluster (clusters S1-S4); Desert supercluster (clusters D1-D5). Plot provenance is also displayed. (ZIP) [file pone.0202073.s010.zip › Cluster D5 WAACOO0029.jpg]

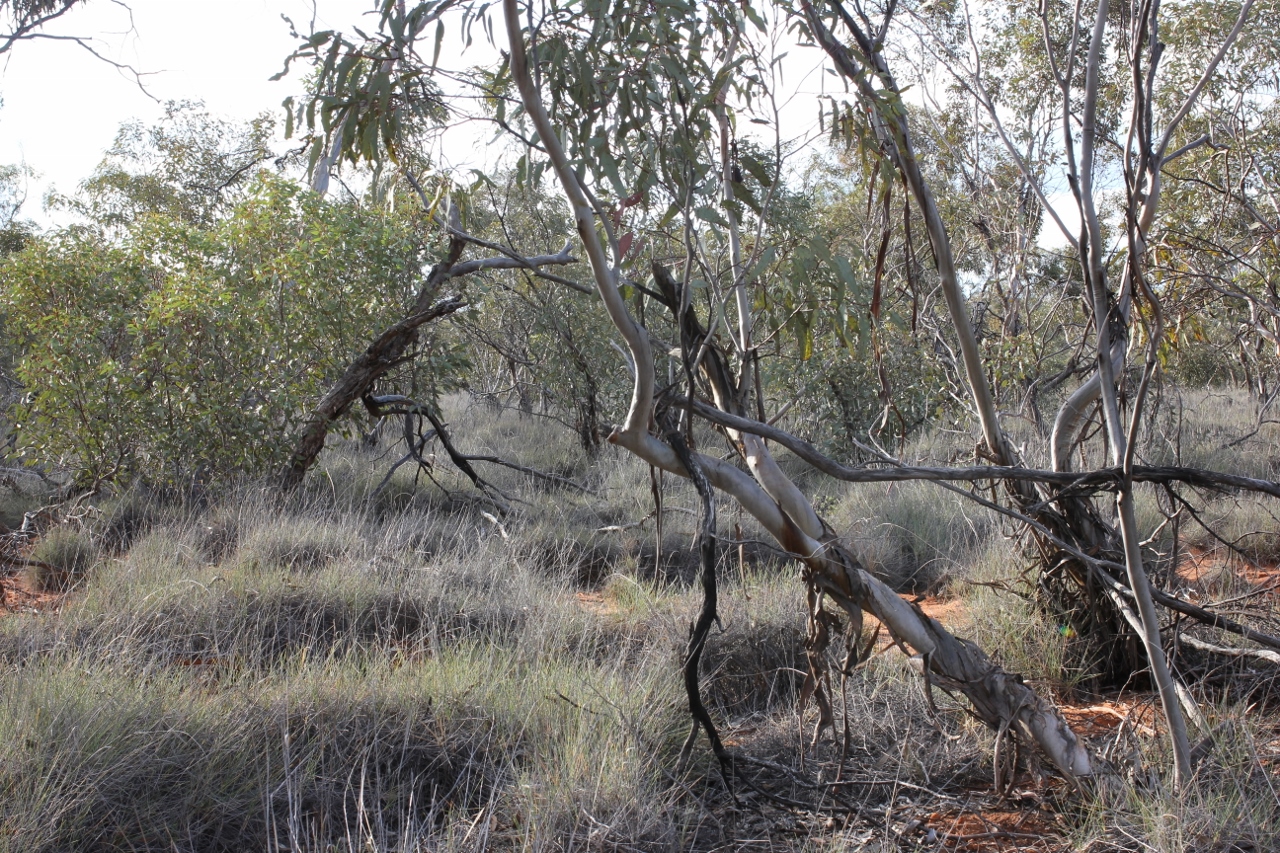

Supplement: S4 File — Mediterranean supercluster (clusters M1-M2); Savanna supercluster (clusters S1-S4); Desert supercluster (clusters D1-D5). Plot provenance is also displayed. (ZIP) [file pone.0202073.s010.zip › Cluster M1 NSAMDD0014.jpg]

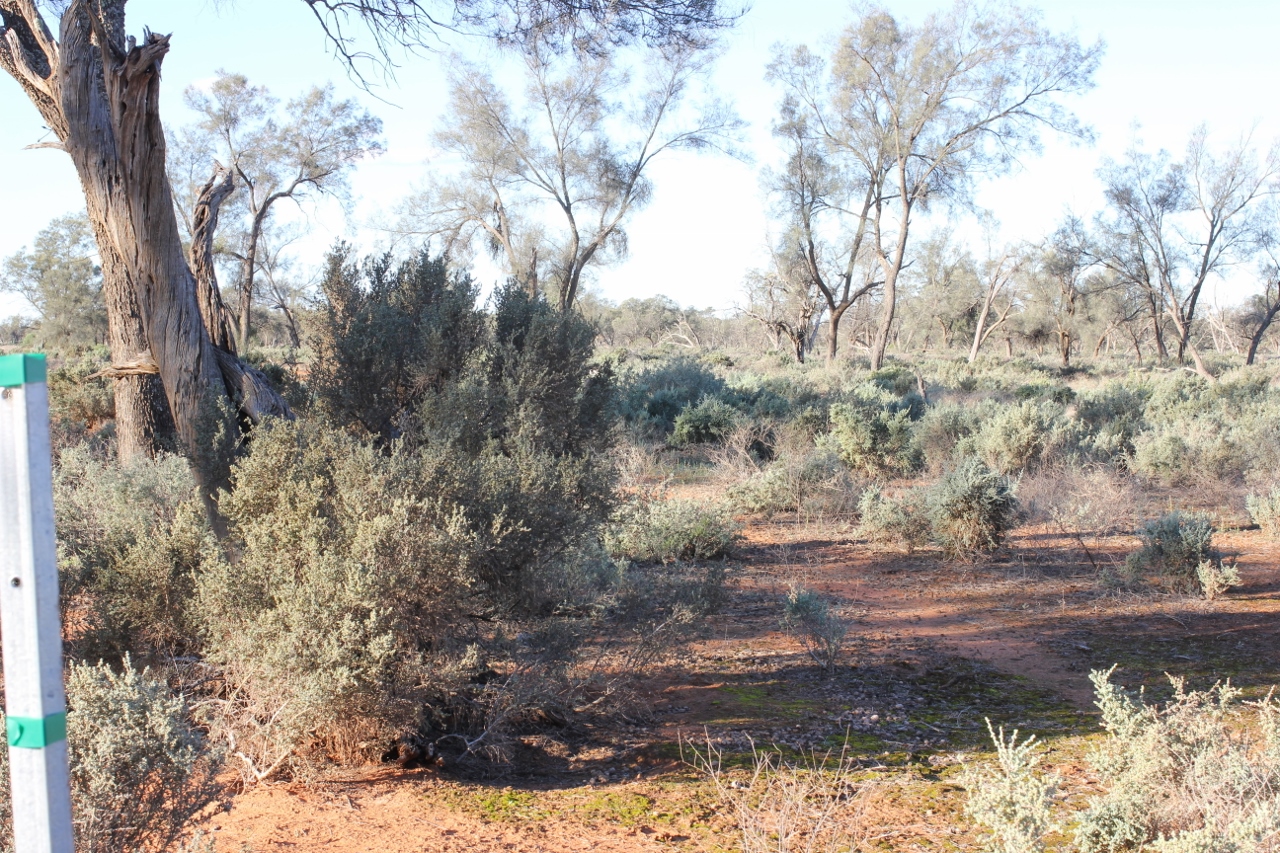

Supplement: S4 File — Mediterranean supercluster (clusters M1-M2); Savanna supercluster (clusters S1-S4); Desert supercluster (clusters D1-D5). Plot provenance is also displayed. (ZIP) [file pone.0202073.s010.zip › Cluster M1NSAMDD0006.jpg]

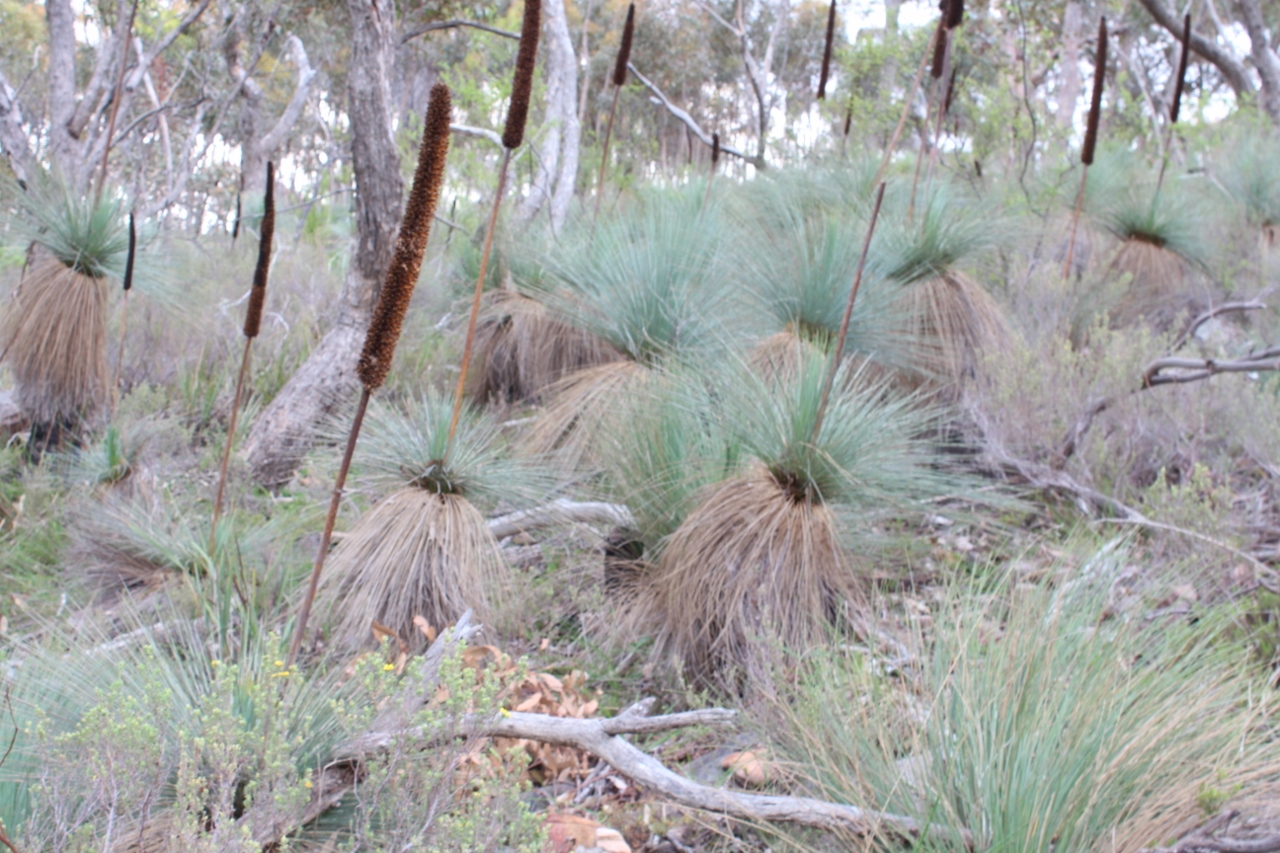

Supplement: S4 File — Mediterranean supercluster (clusters M1-M2); Savanna supercluster (clusters S1-S4); Desert supercluster (clusters D1-D5). Plot provenance is also displayed. (ZIP) [file pone.0202073.s010.zip › Cluster M2 SATFLB0011.jpg]

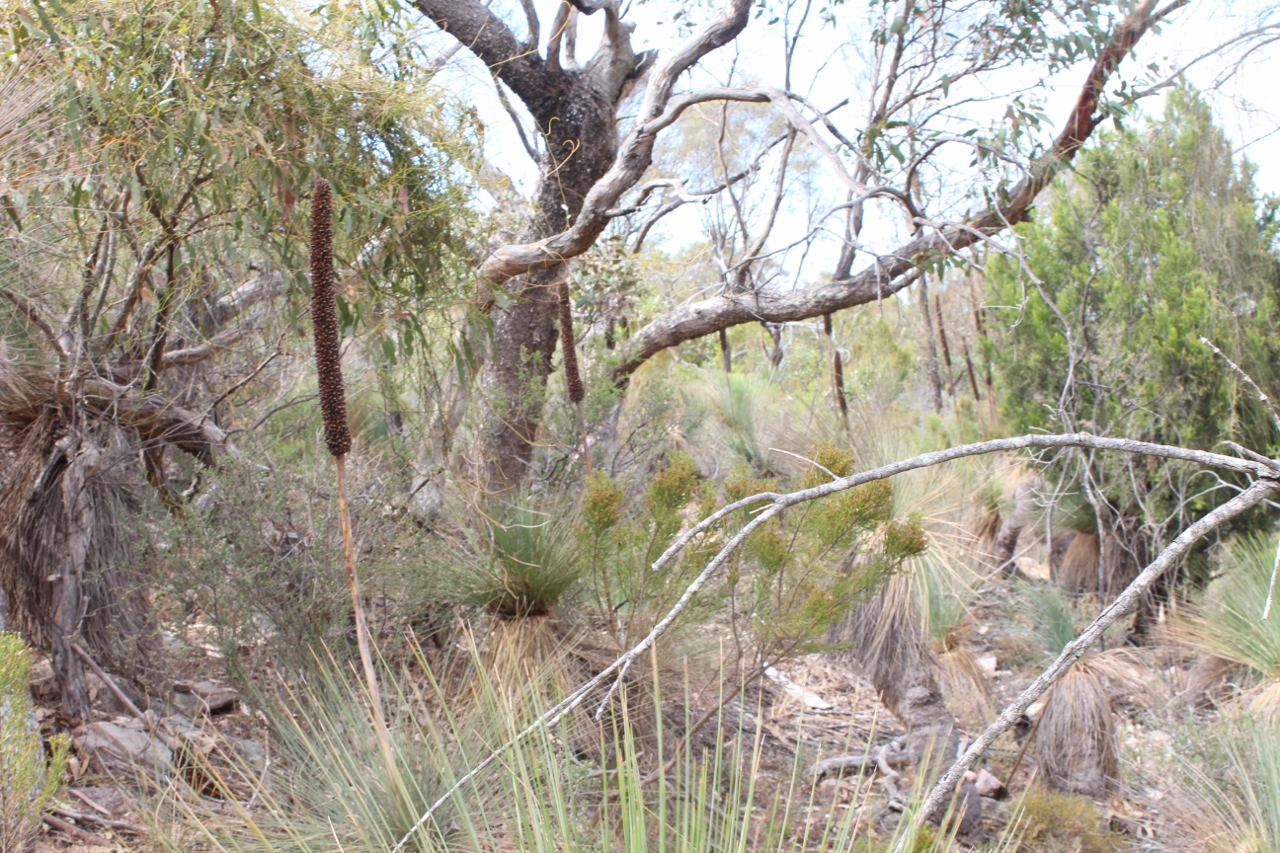

Supplement: S4 File — Mediterranean supercluster (clusters M1-M2); Savanna supercluster (clusters S1-S4); Desert supercluster (clusters D1-D5). Plot provenance is also displayed. (ZIP) [file pone.0202073.s010.zip › Cluster M2 SATFLB0014.jpg]

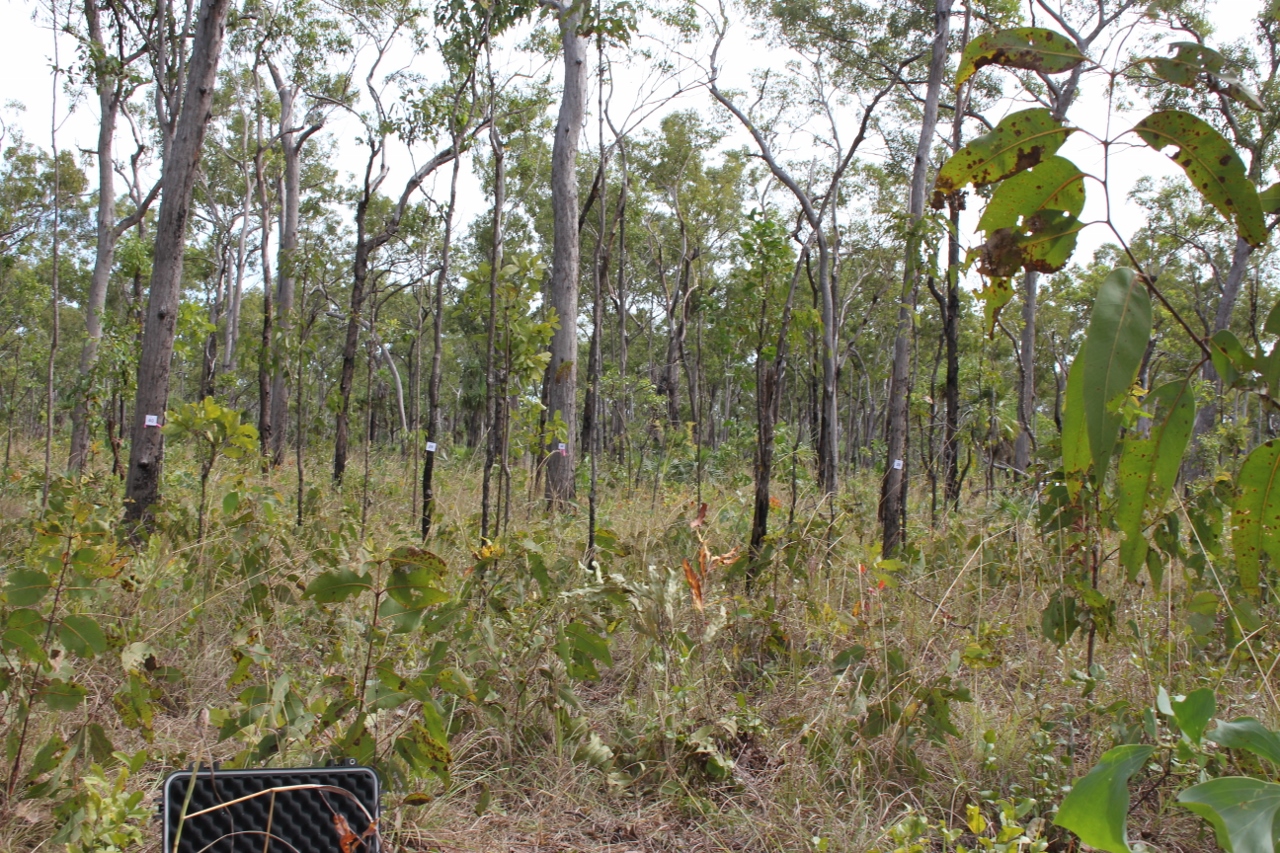

Supplement: S4 File — Mediterranean supercluster (clusters M1-M2); Savanna supercluster (clusters S1-S4); Desert supercluster (clusters D1-D5). Plot provenance is also displayed. (ZIP) [file pone.0202073.s010.zip › Cluster S1 NTADAC0001.jpg]

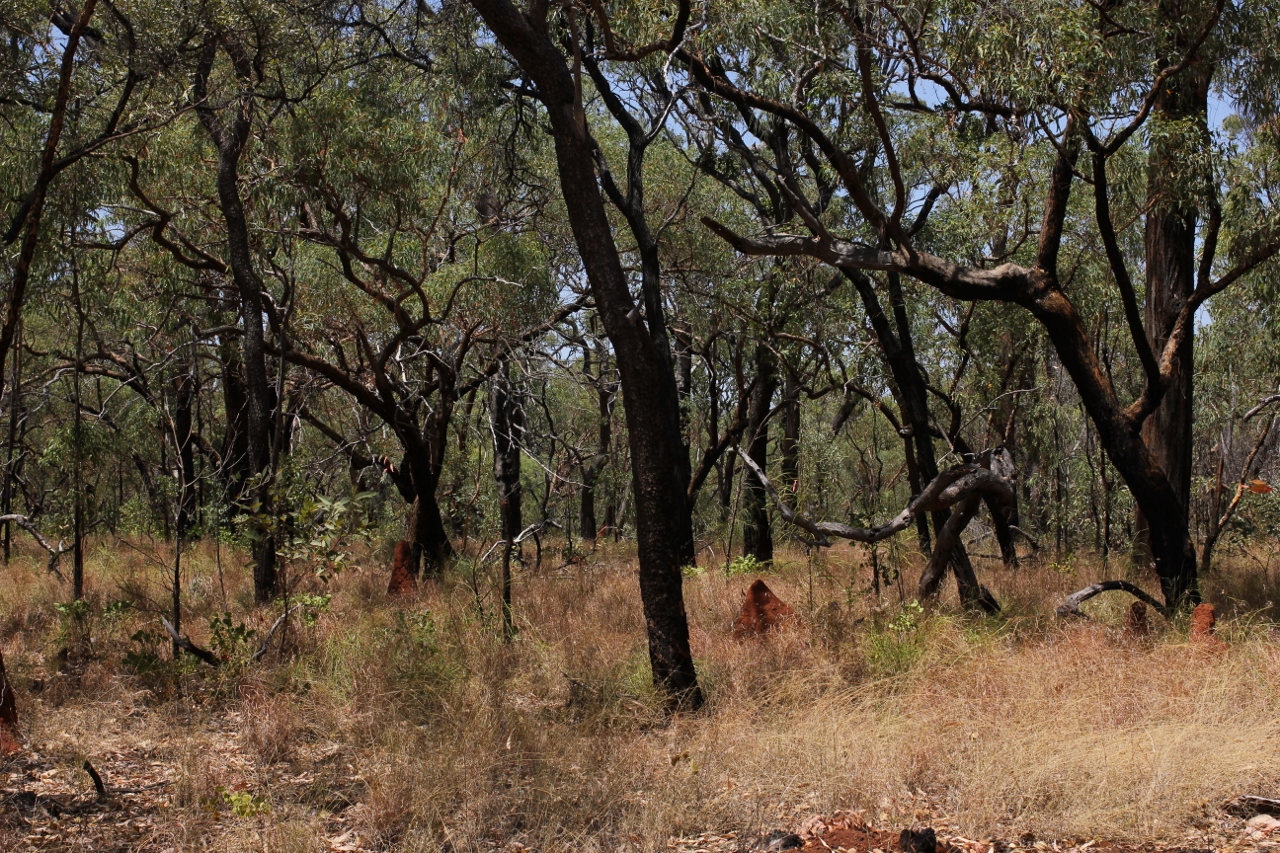

Supplement: S4 File — Mediterranean supercluster (clusters M1-M2); Savanna supercluster (clusters S1-S4); Desert supercluster (clusters D1-D5). Plot provenance is also displayed. (ZIP) [file pone.0202073.s010.zip › CLUSTER S1 QDAEIU0006.jpg]

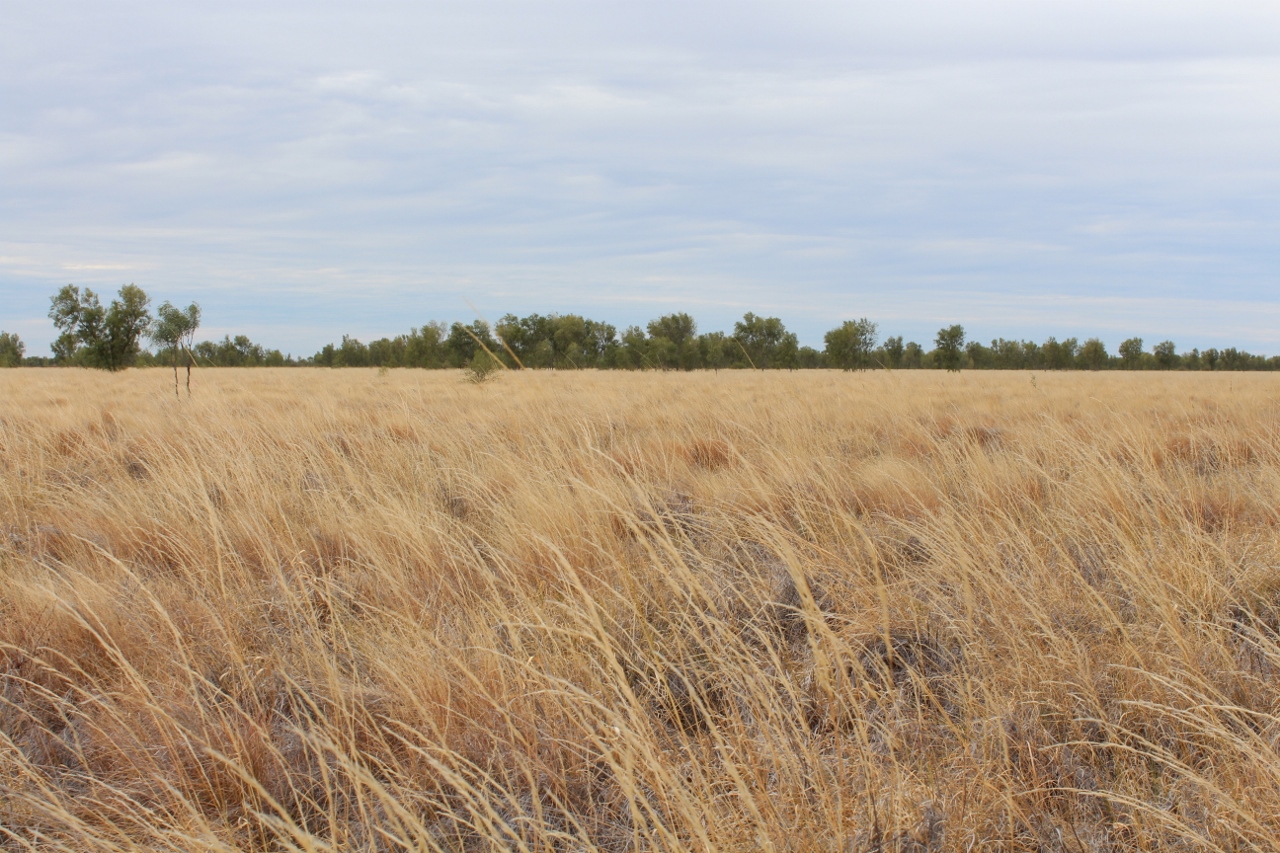

Supplement: S4 File — Mediterranean supercluster (clusters M1-M2); Savanna supercluster (clusters S1-S4); Desert supercluster (clusters D1-D5). Plot provenance is also displayed. (ZIP) [file pone.0202073.s010.zip › Cluster S2 QDAGUP0009.jpg]
